# Exploring Nanoscale Perovskite Materials for Next-Generation Photodetectors: A Comprehensive Review and Future Directions

**DOI:** 10.1007/s40820-024-01501-6

**Published:** 2024-09-30

**Authors:** Xin Li, Sikandar Aftab, Maria Mukhtar, Fahmid Kabir, Muhammad Farooq Khan, Hosameldin Helmy Hegazy, Erdi Akman

**Affiliations:** 1 State Key Laboratory of Pulsed Power Laser Technology, National University of Defense Technology, Hefei, 230037 Anhui People’s Republic of China; 2 Anhui Laboratory of Advanced Laser Technology, Hefei, 230037 Anhui People’s Republic of China; 3 Nanhu Laser Laboratory, Changsha, 410015 Hunan People’s Republic of China; 4 Department of Semiconductor Systems Engineering and Clean Energy, Sejong University, Seoul, 05006 Republic of Korea; 5 Department of Artificial Intelligence and Robotics, Sejong University, Seoul, 05006 Republic of Korea; 6 School of Engineering Science, Simon Fraser University, Burnaby, BC V5A 1S6 Canada; 7 Department of Electrical Engineering, Sejong University, Seoul, 05006 South Korea; 8 Department of Physics, Faculty of Science, King Khalid University, P.O. Box 9004, Abha, Saudi Arabia; 9 Central Labs, King Khalid University, AlQura’a, P.O. Box 960, 61413 Abha, Saudi Arabia; 10 Scientific and Technological Research and Application Center, Karamanoglu Mehmetbey University, 70100 Karaman, Turkey

**Keywords:** Nanoscale perovskites, Photodetectors, Nanosheets, Nanorods, Nanowires, Quantum dots, Nanocrystals

## Abstract

The rapid advancement of nanotechnology has sparked much interest in applying nanoscale perovskite materials for photodetection applications. These materials are promising candidates for next-generation photodetectors (PDs) due to their unique optoelectronic properties and flexible synthesis routes. This review explores the approaches used in the development and use of optoelectronic devices made of different nanoscale perovskite architectures, including quantum dots, nanosheets, nanorods, nanowires, and nanocrystals. Through a thorough analysis of recent literature, the review also addresses common issues like the mechanisms underlying the degradation of perovskite PDs and offers perspectives on potential solutions to improve stability and scalability that impede widespread implementation. In addition, it highlights that photodetection encompasses the detection of light fields in dimensions other than light intensity and suggests potential avenues for future research to overcome these obstacles and fully realize the potential of nanoscale perovskite materials in state-of-the-art photodetection systems. This review provides a comprehensive overview of nanoscale perovskite PDs and guides future research efforts towards improved performance and wider applicability, making it a valuable resource for researchers.

## Highlights

- Innovative synthesis method for nanoscale-based perovskites with enhanced stability and efficiency.
- Novel application of nanoscale-based perovskites in optoelectronics with superior performance metrics.
- Comprehensive analysis of the structure–property relationships in perovskite nanomaterials.

## Graphical abstract

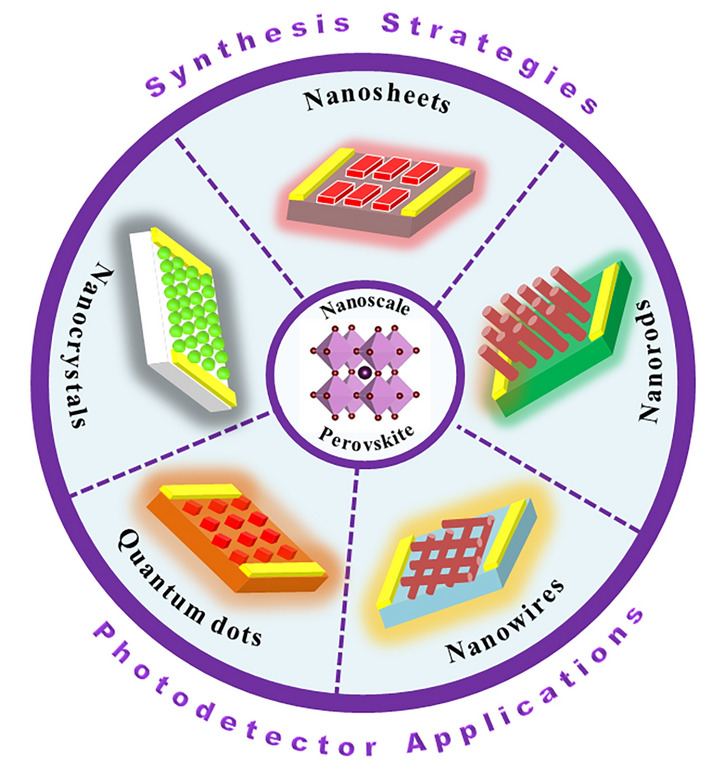

## Introduction

### Overview of Photodetectors

Electronic devices known as photodetectors (PDs) sense and measure light (photons) and convert it into an electrical signal using a variety of types and unique operating principles [1]. When light is absorbed in their depletion regions, for instance, photodiodes use semiconductor structures like p–n junctions or p–i–n configurations to produce a photocurrent [2]. Avalanche photodiodes are perfect for low-light applications because they increase sensitivity through internal avalanche multiplication. Metal–semiconductor–metal PDs offer higher bandwidth capabilities up to hundreds of gigahertz because they use Schottky contacts instead of p–n junctions. Although they are less widely used than photodiodes, phototransistors internally amplify photocurrent. Long-wavelength infrared light can be detected by photoconductive detectors, such as those based on cadmium sulphide, which are less expensive but have slower response times and nonlinear characteristics. Photomultiplier tubes use electron multiplication to achieve high sensitivity, and charge-coupled devices are arrays that convert light into electric charge and are widely used in imaging. Researchers are working on quantum dot PDs for infrared detection, which use quantum dots as sensitive materials. A PD’s wavelength range, sensitivity, speed, and cost are important considerations for various applications, from optical communications to astronomical observations. Optimizing performance in various applications requires an understanding of and approach to mitigating background noise in PDs [1].

**Fig. 1:**
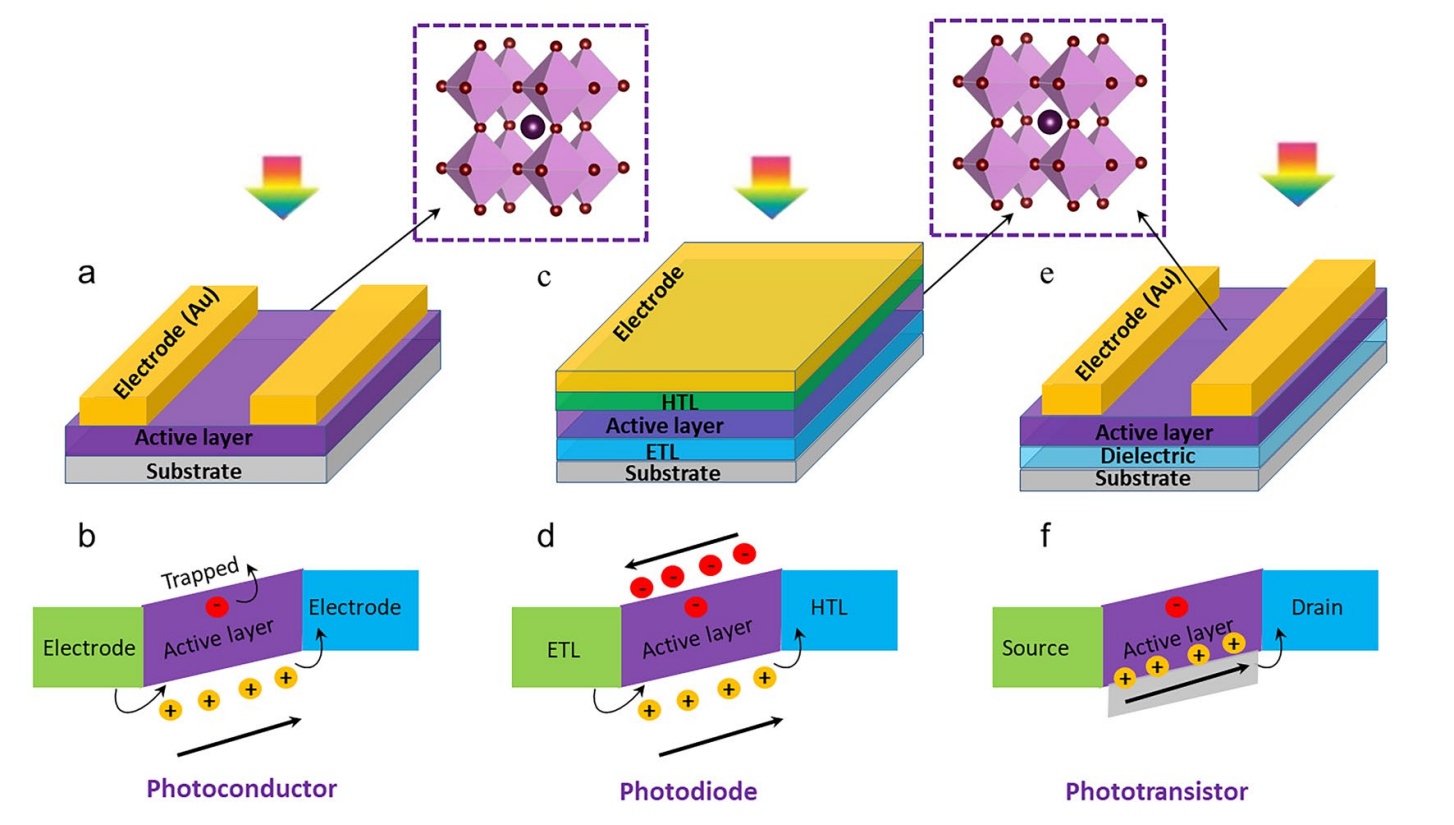
Representative exhibition of classification of perovskite PDs and operational principles of the photoconductors (**a**, **b**), photodiodes (**c**, **d**), and phototransistors (**e**, **f**)

Perovskite-based PDs have attracted much attention because of their remarkable photoelectric properties, including their better capacity to harvest light, adaptable band gap, and carrier migration behaviour [3–11]. Ongoing study in this area focuses on materials synthesis, device structure design, and interface engineering to enhance device properties such as stability, sensitivity, and response speed [4]. According to the different device architectures or mechanisms, perovskite PDs are available in three primary types: photoconductor, photodiode, and phototransistor [2, 12, 13]. Among these types, the photoconductor has garnered special attention because of its integration and ease of use advantages. A semiconductor with two Ohmic metal contacts is a photoconductor (Fig. 1a). The devices become more conductive when a bias voltage is provided to separate the photogenerated charge carriers, then directly dissociated into holes and electrons, and collected by the electrodes (Fig. 1b). The high photoconductive gain in photoconductors results in high responsivity and external quantum efficiency (EQE) when the photoexcited electron (hole) loops through an external circuit multiple times before recombination with a hole (electron) [14, 15]. The photoconductor’s constant slow response time leads to a low specific detection rate. Some outstanding strategies have been developed to promote the performance of PDs, such as novel topographical templates for more quality perovskite materials, enhanced quality of perovskite film with trend synthesis techniques, and effectively integrated device architectures promote the performance of PDs [7]. For example, in an interesting report [16], by manipulating the dewetting dynamics of precursor solution over an asymmetric wettability topographical template, stable α-CsPbI_3_ perovskite nanowire arrays with large grain size, high crystallinity, regulated alignment, and position are demonstrated. Stable α-CsPbI_3_ perovskite nanowire arrays can produce photoconductor-type PD with substantially higher responsivity (1294 A W^−1^), detectivity (2.6 × 10^14^ Jones), and longer-term stability than thin-film devices. In another report, methylamine was applied to the MAPbI_3_/PbI_2_ perovskite layer by Li et al. [17]. The MAPbI_3_/PbI_2_ hybrid film was treated with methylamine, which resulted in a pure MAPbI_3_ perovskite film with better film quality. The film demonstrated a very high responsivity of 3.6 A W^−1^ and a detectivity of 5.4 × 10^12^ Jones when exposed to 0.5 mW cm^−2^ of white light. Furthermore, the film demonstrated its maximum responsivity and detectivity of 30 A W^−1^ and 2.4 × 10^14^ Jones, respectively.

Another significant form of PDs is photodiode, which commonly relies on p–n, p–i–n, or Schottky junction (Fig. 1c). Under light illumination, the perovskite created electron–hole pairs. The electrons and holes are then separated by the junction’s inherent electric field and collected by the electrodes (Fig. 1d). In opposite paths towards the collecting electrodes of photoconductors, the electrons and holes are driven to move, and the electrodes have fast response times and high specific detection rates [18, 19]. Thus, when the devices operate at zero bias, self-powered PDs can be realized efficiently by separating photogenerated electron–hole pairs. However, they display limited EQE and responsiveness, two main drawbacks of photodiodes. Most work on photodiodes has gone towards improving detectivity by lowering device noise, improving response speed by raising carrier mobility, and expanding/contracting the photon response spectrum. Dou et al. [20] used the outstanding optical and electrical properties of organic/inorganic hybrid halide perovskite materials and the distinctive interface design to create a photodiode sensitive in the UV–visible range. The PDs have a large detectivity (the capacity to detect weak signals) of about 10^14^ Jones operating at room temperature. They also have a linear dynamic range of more than 100 dB and a quick photoresponse with a 3-dB bandwidth up to 3 MHz. More strikingly, with PEDOT: PSS and PCBM as the interface materials, the device’s response time was approximately 30 ms, 10^5^ times slower than the inverted device. Very recently, efficient p-type doping of MoS_2_ is achieved through straightforward van der Waals interactions with a 2D perovskite layer, and a high-performance photodiode based on a homogeneous MoS_2_ p–n junction is built [21]. In device performance, the ideality factor is 1.3, and the dark current can be as low as 10^–12^ A. When illuminated, the device’s open circuit voltage can be as high as 0.7 V. Furthermore, the device demonstrates a quick response time of 105/109 µs and a high responsivity of 529 mA W^−1^ under zero bias.

As for the phototransistor, compared to a photoconductor or a photodiode, it is a three-terminal device (Fig. 1e), meaning a more complex fabrication process. In the dark, the phototransistor’s off-state current is often quite low. The perovskite produces electron–hole pairs in response to light; only one kind of carrier can be altered and take part in channel conduction (Fig. 1f). However, because of the transistors’ built-in amplification function, which results in high internal photocurrent gain, phototransistor responsivity and detectivity may be about three orders of magnitude higher than those of photoconductors and photodiodes [8, 12]. Additionally, phototransistors can significantly reduce the dark current and increase the photocurrent by using the grating effect and gate modulation. This helps achieve high photoresponsivity/sensitivity and good detection performance.

Perovskite PDs can be used in optical communications, artificial intelligence, sensing, medical imaging, and night vision because of these characteristics [22]. The current efforts include approaches to electrically modulating perovskite-based PDs to enhance their light-sensing performance for applications such as wearable health monitoring and low-light detection [23]. Numerous PD applications, including polarized light applications, biological detection, optical communication, and imaging, demonstrate potential for these devices [24–26]. Perovskite-based PDs promise to advance the next generation of artificial intelligence and neuromorphic computing is further demonstrated by research into them for synthetic photonic synapses [27–29].

### Nanoscale Perovskite-Based Materials for Photodetection

Due to its exceptional photophysical and photoelectric qualities, including its long carrier diffusion length, substantial absorption coefficient, relatively low defect density, and adjustable optical properties, halide perovskite has been the subject of intensive research for a lot of potential optoelectronic device applications from solar cells to PDs. The typical unit cell of a perovskite crystal is ABX_3_, where X can be a halogen anion or other halogen doping centres (Cl^−^, Br^−^, I^−^), A can be an organic or inorganic cationic substance (Cs^+^, Rb^+^, MA^+^, FA^+^), and B might be a divalent metal cation (Pb^2+^, Sn^2+^, Bi^2+^). The A cations occupy eight corners; the body centre is by the B ions, and the six-face centre is by the X anions. In most cases, the B ion works in tandem with six X anions to create the (BX_6_)^4−^octahedron, which is extended to 3D perovskites and joined by common corners [5, 30, 31]. High-quality perovskite films for PD applications have been produced through the effective use of several common techniques, including inkjet-printed, antisolvent vapour-assisted, vacuum flash-assisted solution processing, atmospheric control, microchannel-confined crystallization thermal annealing, and solvent engineering [3, 32, 33]. However, conventional 3D perovskites are inherently unstable against ambient moisture, which significantly limits their application in high-performance PDs since they are unable to transduce the signal repeatedly over extended periods. Moreover, it is imperative to address the instability of 3D perovskites before using them in real optoelectronic applications. To solve this situation, perovskite single crystals could present low trap density, low intrinsic carrier concentration, high mobility, and long diffusion length over polycrystalline films with morphological disorder. These characteristics make the material ideal for simultaneously realizing rapid and sensitive photodetection [18, 34]. Another effective alternative to this situation is to adjust the morphology of the nanoscale-based perovskites, and these materials can be categorized as zero-dimensional (0D), one-dimensional (1D), and two-dimensional (2D) at the low-dimensional level. The standard formula for 2D perovskite is (LOC)_*m*_(A)_*n*−1_B_*n*_X_3*n*+1_, where n is the layer number of the 2D perovskites, and LOC is the larger organic cation. 2D perovskite is created by inserting LOC layers into the octahedral network of 3D perovskite. LOCs envelop the extremely deformed coplanar octahedra [BX_6_]^4−^ in 1D perovskite. 0D perovskite is produced by further decreasing the connectivity of the octahedral [BX_6_]^4−^ network [35]. The spatial dimensions of perovskite nanomaterials are used to categorize them: 0D nanomaterials, including quantum dots and nanoparticles, contain all dimensions within the nanoscale (1–100 nm) [35, 36]. 1D nanomaterials, which include nanorods, nanowires, and nanotubes, enlarge one dimension beyond the nanoscale while containing the others. 2D nanomaterials, such as nanosheets, have two dimensions that extend two dimensions beyond the nanoscale and thickness at the nanometre level [36]. These materials differ from bulk materials due to their distinctive qualities, which include high surface-to-volume ratios and quantum confinement effects. They are extremely valuable in many fields because of their unique physical, chemical, and electronic properties [36–43]. This dimensional classification framework makes it easier to comprehend and create nanomaterials that are suited to particular uses.

Preparing nanosheet/plate perovskite crystals for use in PDs involves a combination of solution-phase growth, vapour-phase conversion, and one-step chemical vapour deposition (CVD) [44–46]. On the other hand, centimetre-sized free-standing perovskite NSs made from a solution comprising a mixture of solvents exhibit atomically thin 2D structures [47]. The superior characteristics of perovskite single crystals, such as their longer carrier diffusion lengths and lower trap-state densities, provide an understanding of the potential benefits of these materials [48]. Furthermore, a strategy to improve structural stability and overcome difficulties in synthesizing organic–inorganic lead halide perovskite (LHP) single crystals has been suggested: the growth of thin single crystals at micro-/nanosizes [49]. This suggests that properties may be improved. These contrasts draw attention to the various properties and possible advantages of perovskite crystalline NSs produced by various methods. Compared to conventional semiconductors, using perovskite NSs in PDs has several benefits. These advantages include easy integration into PD applications, extended carrier lifetimes, low cost compared to conventional materials, and straightforward preparation procedures [50–52]. Perovskite NSs, like CsPb_2_Br_5_ NSs, have improved PDs’ performance in optoelectronic devices by showcasing their stability and developmental potential [53]. Perovskite NSs’ unique qualities make them extremely well suited for photodetection applications, demonstrating their potential to advance this technology.

While the solution-phase synthesis approach is primarily responsible for the halide perovskite NWs reported in photodetection applications, it is well-known that many techniques are employed to produce NWs. Fabricating NWs through modified evaporation and the nanoengineering template strategy have been manufactured [54]. Using simple fabrication techniques that provide high performance and economy makes perovskite materials favourable for the production of PDs [22]. Perovskite-based PDs are divided into photovoltaic and photoconductive devices according to their spatially arranged; these devices can be used as phototransistors, photodiodes, and photoconductors [51]. Nanostructured perovskites, which include 0D, 1D, and 2D structures, are used to improve photodetection efficiency and obtain high detectivity, large gain, high photoresponsivity, and fast response times [51]. Cl-type perovskite devices have the potential to achieve high stability and performance through the integration of CsPbI_3_ NRs as an interfacial layer to enhance perovskite PDs’ performance [55]. The methods that are currently being used to grow perovskite NWs include effectively aligning free-standing NWs and heterojunction arrays vertically [56]; using inorganic perovskite NWs to passivate defects at grain boundaries [57]; utilizing fabrication techniques like inkjet printing and nanopore-confined growth for precise alignment in flexible patterning for large-scale lighting applications inducing controlled growth for particular morphologies like CsPbBr_3_ NWs [58]. Despite these developments, patterning, aligning, and transferring perovskite NWs for lighting and nanophotonics still present difficulties [58].

Perovskite quantum dots (QDs) are very interesting because of their unique properties and possible applications. These microscopic crystals are created by intricate processes that regulate their formation and growth rates, yielding homogeneous QDs with sizes ranging from 3 to > 13 nm [59]. Their absorption spectra can reveal up to four excitonic transitions, and their size and shape dictate their properties [59]. These QDs have variable electrical and optical properties, making them useful for applications such as solar cells, LEDs, catalysis, sensing, imaging, and lasers [60]. Hot injection [61] and ligand-assisted reprecipitation [62] are the primary techniques suggested for producing the extensively researched perovskite QDs. Also, their synthesis is carried out at room temperature, which enables exact control over the size and content of the formed spherical QDs. These dots release a wide range of colours depending on their size, making for efficient emissions. Furthermore, the emission wavelength may be precisely adjusted by adjusting the halogen ratios and doping the B-site [60, 63].

Perovskite nanocrystals (NCs) have become a popular substitute in PD research due to their exceptional photoelectric properties. These NCs feature a higher light harvesting capability, an effective carrier migration behaviour, and an adjustable band gap, among other advantages [64]. Recent research has focused on various subjects to enhance devices’ stability, sensitivity, and response speed: materials synthesis, interface engineering, device design, and physical mechanisms [64]. Perovskite-based PDs exhibit a spectrum of molecular structures from 0 to 3D and are available in multiple forms, including perovskite photoconductors, photodiodes, and phototransistors [64]. These tips are used in many industries, such as self-powered PDs, imaging, biological detection, and optical communication [64]. Perovskite NCs possess unique optical and electrical properties, including a high absorption coefficient and an extended exciton diffusion length [65]. Their composition engineering allows them to be tuned throughout various wavelengths, from ultraviolet to near-infrared [65]. This makes them incredibly purposive. Perovskite NCs are also popular for high-performance PDs because of their ability to process solutions [65]. The ability to detect optical data such as spectrum, polarization, and incidence angle has been made possible by notable advances in the study of multifunctional perovskite-based PDs [35]. In order to maximize light absorption and charge transport inside the films, perovskite crystal structure design is essential. High-performance PDs that operate in the near-infrared to X-ray range frequently employ perovskite materials [35]. In summary, perovskite NCs have enormous potential for future advancements because they may be used to create high-performance PDs for a wide range of uses.

### Multidimensional Photodetection Capabilities of Nanoscale Perovskite-Based Materials

Perovskite PDs at the nanoscale have proven remarkably adept at identifying multidimensional features in light fields beyond simple intensity. One of the main features is the ability to detect polarization, which can be done using nanostructured designs that show polarization-dependent absorption, charge transport, or engineered anisotropic crystal structures [35]. Perovskite composition and band gap tuning enable selective sensing across ultraviolet, visible, and infrared wavelengths and spectral discrimination within a single device, making spectral detection possible [35]. Thanks to specialized geometries or anisotropic material properties, some PD architectures can respond differently depending on the angle of incident light, facilitating angle-sensing detection [35]. The photovoltaic effect allows for self-powered operation and independent light sensing without external power sources. The solution processability of perovskite makes flexible PDs possible. These PDs can withstand bending and deformation and thus enable conformal sensing on curved surfaces [66]. Additionally, capable of multifunctional detection, advanced perovskite PDs can record parameters like intensity, wavelength, polarization, and incident angle simultaneously in one device. Additionally, capable of multifunctional detection, advanced perovskite PDs can record parameters like intensity, wavelength, polarization, and incident angle simultaneously in one device [66]. These varied detection capabilities use tunable optoelectronic characteristics, adaptable crystal structures, and controllable nanoscale morphology provided by perovskite materials. Researchers can create miniature perovskite PDs that extract detailed information from incident light fields, going beyond conventional intensity-based measurements, by carefully planning the composition, structure, and device architecture [35].

Perovskite PDs use polarized light detection to exploit the materials’ distinct anisotropic crystal structure and controllable orientation growth. These characteristics are essential for polarization detection because they allow the material to react to light waves differently depending on which way they are blowing [67, 68]. The PDs can be optimized for polarization detection by adjusting the growth orientation of the perovskite crystals during the fabrication process [50, 51]. To detect differences in light polarization, perovskites’ strong light absorption and high carrier mobility improve the efficiency of converting light into electrical signals [50, 51]. The PD reacts to light waves with a fixed polarization direction for linearly polarized light and uses helical 1D perovskite structures to recognize the rotary electric field vector for circularly polarized light [50, 51]. These polarization-sensitive PDs find use in optical communication, biomedical sensing, remote sensing, and military imaging systems [35]. Therefore, the excellent photovoltaic performance of perovskite PDs, their anisotropic properties, and their controlled growth orientation allows for the sensitive and effective detection of polarized light for advanced optical technologies.

This review paper provides a more comprehensive and up-to-date examination of growing methodologies, applications, and possible prospects, building on previous research in nanoscale perovskite-based PDs. While previous studies have concentrated on certain aspects of nanoscale perovskite-based PDs, such as synthesis techniques or device applications, this review combines multiple points of view to offer a thorough overview of the subject. It also covers recent advances and fresh approaches in the field, including examining unique nanoscale perovskite structures and discussing challenges and opportunities. This review summarizes the most recent research findings and provides insights into prospects to contribute to the ongoing discussion and encourage further breakthroughs in nanoscale perovskite photodetection technology systems.

## Wide-Band Gap Semiconductors and Nanoscale Perovskite-Based Photodetectors

Modern optoelectronics depends on precise UV light detection, and the majority of UV photodetectors on the market today use wide-band gap semiconductors (WBSs) [19]. Promising solutions to overcome integration and flexibility constraints are provided by low-dimensional WBSs, opening up new opportunities for advancements in various UV applications such as imaging, wearable electronics, and communication. Semiconductors like silicon carbide (SiC) and gallium nitride (GaN) have a notable energy band gap and display unique electrical properties and capabilities compared to other semiconductors. These materials have a large band gap, which means they are highly resistant to breakdown or performance loss when exposed to high temperatures and voltages. As a result, they provide exceptional performance in power electronics and RF amplifiers that necessitate high power and frequency. Moreover, it has exceptional heat transfer properties, facilitating swift heat dissipation. This improves their stability and reliability in challenging settings [19, 69, 70]. Nanoscale perovskites have unique structural traits and electrical conduction capabilities that differentiate them from wide-band gap semiconductors. Modifying certain components of the perovskite crystal structure allows for attaining a wide range of electrical properties. These materials offer significant benefits for electronic applications due to their ability to be easily scaled and adjusted, thanks to their manufacturing using cost-effective solution-based processes [71–73]. Perovskites demonstrate exceptional electrical conductivity and carrier mobility. Nevertheless, their capacity to maintain stability at high temperatures is lower than wide-band gap semiconductors. This suggests they may gradually deteriorate if exposed to high temperatures and humid environments [74, 75]. Perovskites’ and WBSs’ unique physical and electrical features determine their usage. Wide-band semiconductors surpass perovskites in terms of both high power and high temperatures. On the other hand, perovskites show potential for diverse and affordable uses in electronics [51, 76]. The advantages and disadvantages of nanoscale perovskite-based photodetectors and WBSs are displayed in the Table 1. Table 2 displays the different approaches for creating nanoscale perovskite-based materials. Table 3 lists the advantages and disadvantages of materials based on nanoscale perovskite technology. Table 4 displays the different parameters for perovskite photodetectors based on nanoscale technology.

**Table 1:** Advantages and disadvantages between wide-band gap semiconductors and nanoscale perovskite-based photodetectors

Criteria	Wide-band gap semiconductor-based photodetector	Nanoscale perovskite-based photodetector
Thermal stability	Specifically engineered for utilization in environments with elevated temperatures, it is very suitable for such circumstances [77]	Displays a moderate level of stability but may see a decline in quality when subjected to high temperatures [78]
Responsivity	The responsivity can vary from modest to high, contingent upon the substance [19]	The responsivity is significantly elevated in the visible spectrum [79]
UV sensitivity	Ideal for detecting UV light [19]	Variable, less effective in ultraviolet detection [79]
Manufacturing		
Cost	The exorbitant expense is ascribed to complex manufacturing techniques	More easily available, capable of being transformed into a solution, and less complex
Material Abundance	Limited, contingent upon finite resources	Employs components that are abundant and less detrimental
Efficiency	High, exhibiting a significant level of quantum efficiency	High, having the potential to attain even higher levels of efficiency
Lifetime and Stability	Durable and stable [19]	The mild condition may worsen with time, especially when exposed to UV light [80]
Application Suitability	Ideal for complex tasks requiring substantial power, elevated temperatures, and exposure to ultraviolet light [19]	Ideal for cost-efficient, versatile applications that function within the visible range [79]

**Table 2:** Various methods for nanoscale perovskite-based materials

Nanostructure	Synthesis Method	Advantages	Challenges	Device performance	Unique Properties	References
Quantum Dots (QDs)	Hot-Injection Technique	High luminescence Controlled size and shape Good crystalline quality	Complex set-up Batch variability	High sensitivity and responsivity Stability and reproducibility	High quantum yield Narrow size distribution	[83]
	Ligand-Assisted Reprecipitation (LARP) Synthesis	Simplicity and scalability Ambient condition fabrication Using versatile solvent	Shape and size inconsistency Solvent residue	Broad spectral response Cost-effective	Flexible composition Low-cost production	[84–88]
	Solvothermal Synthesis	High crystallinity and purity Controlled morphology Tailored optical properties	High-pressure and high-temperature requirement Scalability issue	Improved efficiency Long-term stability	High purity Enhanced stability	[89–92]
	Microwave-Assisted Synthesis	Rapid synthesis Uniform particle size Energy efficient process	Uniform heating challenges Specialized Equipment needed	Consistent performance	Fast reaction time High throughput	[93–96]
	Ultrasonic-Assisted Synthesis	Enhanced mixing Controlled size distribution Room-temperature process	Uniform sonication challenges Potential degradation	Consistent light absorption due to controlled size distribution	Improved homogeneity Simple and scalable	[97–101]
Nanosheets (NSs)	Solution-phase exfoliation	Scalable Simple Procedure Versatile solution for tailoring properties	Variable quality Solvent residue	High sensitivity Enhanced responsivity	Thin uniform sheet Adaptable to different perovskite composition	[92, 102–105]
	Top-down exfoliation	High-quality nanosheets No chemical residue Control over thickness	Low yield Labour-intensive	High efficiency Reliable performance	Defect free nanosheet Thickness control	[9, 102, 106–108]
	Solvothermal	High crystallinity Uniform thickness Tailored properties	Complex process Scalability issue	Enhanced photodetection Stability and durability	High purity and crystallinity Tunable properties	[100, 109–112]
	CVD	High-quality film Scalable Precise thickness control	High cost Complex process	High responsivity Scalable manufacturing	Large area uniformity Controlled area growth	[3, 113–116]
	Ligand-Assisted Reprecipitation (LARP) Synthesis	Simple and low cost Ambient condition fabrication process Versatile perovskite composition	Inconsistency in thickness Solvent residue	Cost-effective Broad spectral response	Flexible composition Ease of synthesis	[92, 117–120]
Nanorods (NRs)	Hot-Injection Technique	High aspect ratio control High crystallinity Uniform size distribution	Complex set-up Batch Variability	High sensitivity and responsivity Stable performance	Aspect ratio control High crystallinity	[51, 121–124]
	Ligand-Assisted Reprecipitation (LARP) Synthesis	Simple and low cost Ambient condition fabrication process Versatile perovskite composition and solutions	Inconsistence morphology Solvent residue	Cost-effective Broad spectral response	Low-cost production Flexible composition	[76, 119, 125–127]
	Solvothermal	High crystallinity and purity Controlled morphology Scalable	High-pressure and high-temperature requirement Long reaction time	Enhanced Efficiency Long-term stability	High quality Tailored properties	[122, 124, 128–131]
	Colloidal synthesis	Controlled growth High purity Reproducibility	Surface ligand Complex purification	Enhance performance Reproducible results	Size and shape control High optical quality	[89, 132]
	Ultrasonic-Assisted Synthesis	Enhanced mixing Room-temperature process Simple and low cost	Inconsistent sonication Potential material degradation	Consistent light absorption Cost-effective	Uniform nanorod Eco-friendly process	[100, 101, 127, 132, 133]
Nanowires (NWs)	Solution-phase synthesis	Simple procedure Scalable Ambient condition fabrication process	Inconsistent quality solvent residue	Cost-effective Broad spectral response	Low production cost Versatile	[33, 89, 134–136]
	Vapour–liquid–solid	High crystallinity Controlled growth High aspect ratio	High temperature Complex set-up	High sensitivity Stable performance	High-quality nanowire Controlled dimension	[137–141]
	Electrochemical deposition	Precise control Low temperature Scalable	Complex process Potential contamination	Enhanced responsivity Reproducible result	Controlled growth Low energy consumption	[142–146]
	Template-assisted growth	Uniform nanowire High aspect ratio Versatile	Template removal Limited scalability	High sensitivity and uniformity Reliable performance	High uniformity Customizable	[147–151]
	Hydrothermal synthesis	High crystallinity Low cost Eco-friendly	Long reaction time Temperature control	Enhanced Efficiency Stable and durable device	High-quality nanowire Environment-friendly process	[152–155]
Nanocrystals (NCs)	Hot-Injection Technique	High crystallinity Controlled size and shape High quantum yield	Complex set-up Batch variability	High sensitivity and responsivity Stable performance	High quantum yield Controlled morphology	[156–158]
	Ligand-Assisted Reprecipitation (LARP) Synthesis	Simple and scalable Ambient fabrication process Versatile	Inconsistent morphology Solvent residue	Cost-effective Broad spectral response	Low cost Flexible composition	[124, 126, 159, 160]
	Solvothermal Synthesis	High crystallinity and purity Growth control Scalable	High-pressure and high-temperature requirement Long reaction time	Enhanced efficiency Long-term stability	High-quality nanocrystals Tailored properties	[129, 161, 162]
	Microwave-Assisted Synthesis	Rapid synthesis Uniform particle size Energy efficiency	Uniform heating challenges Specialized equipment	Consistent performance High throughput	Fast reaction time Low energy consumption	[163–166]
	Ultrasonic-Assisted Synthesis	Enhanced mixing Room-temperature process Simple and low cost	Inconsistent sonication Potential material degradation	Consistent light absorption Broadband spectral response	Uniform nanocrystal Eco-friendly	[97, 167–169]

**Table 3:** Advantages and disadvantages of nanoscale perovskite-based materials

Nanostructure	Advantages	Disadvantages	References
Nanosheets	• Enhances the ability to absorb and detect light • This phenomenon improves the capacity to absorb and perceive light • The system enables tailored spectral functionality across a wide spectrum of wavelengths • The system can support and integrate flexible and wearable electrical devices • This technology can be utilized with flexible substrates, leading to a reduction in costs • Enhances the velocity and effectiveness of the response • Adjusts optical absorbance following the material’s thickness • PDs composed of materials with wide band gaps exhibit similarities • Facilitates seamless production • Sustains optimal performance over extended periods of air exposure	• Susceptible to deterioration when exposed to moisture and oxygen • Fabrication can pose difficulties, which can impact the ability to reproduce results • Problems related to NSs that impact the reliability of the device. Careful handling is necessary for hazardous items • Advanced procedures are required for the inclusion of complex devices • It is necessary to achieve a balance in performance characteristics • Certain devices possess a limited range of near-infrared (NIR) detection • The use of lithography and metallization complicates production	[107, 170–174]
Nanorods	• The material exhibits exceptional optoelectronic properties, which can be readily achieved through solution processing • Enhanced performance is achieved by extending diffusion length, heightened absorption, and reduced flaws • The material exhibits high light absorption, excellent air stability, and optimal functionality • Detects a broad spectrum of wavelengths • Scalable fabrication techniques and cost-effective materials are employed. Additionally, this approach demonstrates adaptability to various substrates and topologies • The quick transit and separation of charges enhance the signal detection process	• Stoichiometry, humidity, and temperature must be accurately managed during fabrication, complicating it • High carrier diffusion distance and trap density reduce efficiency • Due to heat, light, moisture, and oxygen damage, lifespan and reliability are reduced • Energy-consuming operation requires external bias. Lead misuse endangers public health, safety, and the environment • The annealing atmosphere may restrict detecting region flexibility, compromising device functioning • Manufacturing consistency and volume scaling are difficult, affecting commercial viability	[175–183]
Nanowires	• Enhanced light absorption enhances sensitivity • The rapid charge transport facilitates swift response times • Unimpeded pathways contribute to increased efficiency • Personalized attributes according to individual needs • The system can accommodate diverse architectures, hence promoting adaptability • Optoelectronic applications can benefit from improved sensitivity and efficiency • Superior materials enhance performance • Highly photosensitive, enhancing detection capabilities • Optimal light consumption resulting from minimum transitions caused by impurities • Supports self-powered detectors	• Moisture, oxygen, and light-induced degradation • Difficulties in producing consistent, superior NWs • The operational stability is less predictable due to structural and chemical instability • Worries regarding detrimental substances such as lead • Elaborate and demanding growing process • Challenges in regulating the precise characteristics of NWs • The presence of uncertain growth specifics hinders the issue of reproducibility • Demands expensive oxides or intricate techniques for production • Diminished durability and reliability as a result of susceptibility to environmental factors	[136, 147, 176, 184–187]
Quantum dots	• Improves quantum efficiency by enabling the effective conversion of light into electrical signals • Capable of providing broad-spectrum responsiveness and sensing a broad range of wavelengths, from UV to NIR • Customizable band gaps are possible by tuning the quantum dot size, which affects optical characteristics • Speeds up reaction times by facilitating quick electron–hole pair separation and recombination • Can be manufactured at a lower cost of fabrication by employing techniques that can be solved • Possess superior optical and electrical qualities that make them appropriate for optoelectronic devices • Improved wideband spectrum response (between 300 and 630 nm) through manipulation of the spacer thickness between the membrane of the nanoparticle and the QDs • Long diffusion lengths provide excellent carrier mobility, low operation voltage, and high on/off ratios • Enhanced photoelectric performance, including high detectivity and responsivity, when paired with materials such as graphene • Because of their distinct advantages, they show promise for use in UV light detectors and optoelectronic devices	• Sensitive to external elements such as moisture and UV light, which can cause stability problems • Synthesis becomes more complex due to the difficulty in precisely controlling the size and distribution of QDs • Some are toxic due to the presence of hazardous ingredients • It might be challenging to achieve consistent performance between batches, which can cause problems with repeatability • Operational instability can result from performance variations under various operational settings • Rapid electron–hole recombination rate and relatively low-light-absorption cross section cause quick exciton annihilation and little light gain • Low charge transfer efficiency and light absorption result in limited photoresponsivity • Their performance is frequently poorer when comparing lead-free perovskite QDs to their lead-based counterparts • Nevertheless, restrictions can be lessened by combining graphene for enhanced photoelectrical performance or by striking a balance between plasmonic near-field amplification and surface energy quenching	[66, 188–194]
Nanocrystals	• Improves the absorption of light, raising sensitivity • Detects a wide range of light, from UV to NIR • Rapid charge dynamics are facilitated by small size, which results in prompt response • Appropriate for a range of device topologies, including flexible electronic systems • Synthesized by procedures based on solutions, allowing for large-scale production • Enhances photoresponsivity by extending the response spectrum to include the UV band • Increases carrier collection and electromagnetic field, making it appropriate for gas detection • Promising for quick response times in high-performance PDs	• Sensitive to the environment, which causes deterioration • Complicated to manufacture since exact control over NC size is needed • Some contain harmful elements, raising safety concerns • The difficulty of getting consistent performance impacts scalability • Performance variations under various circumstances influence reliability • The "dead zone" effect makes it difficult to detect UV–visible and infrared bands concurrently • Introduces defects caused by problems with phase segregation and crystallization that impact performance • It is challenging to attain high responsivity and speed at the same time	[172, 175, 195–199]

**Table 4:** Various parameters for nanoscale-based perovskite photodetectors

Photodetector Detection Material	Responsivity (mA/W)	Detectivity (Jones)	Rise/Fall time	On/Off ratio	Detected wavelength (nm)	Applied Bias Voltage (V)	Year	References
*Nanosheet*								
FAPbBr_3_	1.033 × 10^6^	1.2 × 10^13^	25 ms/-	10^4^	545	3.0	2024	[44]
Sr_2_Nb_3_O_10_	3 × 10^8^	–	–	3 × 10^2^	280	5.0	2024	[200]
CsPb_2_Cl_5_	87.4	7.73 × 10^10^	17 ms/32 ms	2.7 × 10^2^	365	1.0	2024	[201]
Cs_2_PbI_2_Cl_2_	698	8.6 × 10^12^	–	–	405	0.0	2024	[202]
Ca_2_Nb_2.5_Ta_0.5_O_10_	60	< 10^13^	0.7 ms/8.5 ms	2.2 × 10^3^	300	2.0	2024	[203]
NbWO_6_	378 × 10^3^	–	1.05/88 ms	8.84 × 10^3^	290	1.0	2024	[204]
MAPbBr_3_	5.04 × 10^3^	5.37 × 10^12^	80/110 µs	–	405	1.0	2023	[205]
CsPbBr_3_	120	9.36 × 10^12^	–	–	405	3.0	2024	[206]
CsPbBr_3_	85	4.05 × 10^11^	3.40 ms/10.20 ms	400	265	3.0	2022	[207]
NdNb_2_O_7_	6.2 × 10^4^	6.7 × 10^12^	0.1 ms/7.8 ms	1250	260	3.0	2023	[103]
Pb_2_Nb_3_O_10_	2.8 × 10^3^	1.1 × 10^12^	0.2 ms/1.2 ms	90	350	0.0	2023	[104]
MAPbBr_3_	27.2 × 10^3^	6.38 × 10^8^	0.103 s/0.087 s	–	300	–	2022	[170]
CsPbCl_x_Br_3−x_	1.96 × 10^3^ 0.12	5 × 10^12^ 2.15 × 10^9^	–	–	440 980	0.5 0.5	2022	[208]
CsPbBr_3_	–	–	–	–	410	3.0	2022	[209]
MAPbBr_3_	24 × 10^3^		3.3 μs/4.0 μs		325	-1.0	2021	[210]
CsPbBr_3_	2.89 × 10^5^	1.28 × 10^14^	0.53 s/0.62 s	1.36 × 10^5^	350	3.0	2021	[211]
Ca_2_Nb_2.5_Ta_0.5_O_10_	469.5 × 10^3^	7.65 × 10^13^	0.4 ms/40 ms	5.6 × 10^4^	295	1.0	2021	[212]
CsPbBr_3_	0.9671 × 10^3^	–	0.122 ms/0.138 ms	5 × 10^3^ 8.47 × 10^3^	520	1.0 5.0	2021	[213]
CsPbBr_1.5_I_1.5_	3313 × 10^3^ 3946 × 10^3^	1.6 × 10^11^	116 ms/147 ms	–	410	1.5 2.0	2021	[115]
Ca_2_Nb_3_O_10_	14.94 × 10^3^	8.7 × 10^13^	0.08 ms/5.6 ms	3.4 × 10^4^	280	3.0	2021	[108]
CH_3_NH_3_PbBr_3_	1.93 × 10^3^	1.04 × 10^12^	24 μs/103 μs		405	2.35	2020	[109]
CsPb_2_Br_5_	75.4	1.33 × 10^10^	43 ms/83 ms	9 × 10^2^	405	5.0	2020	[53]
(PPA)_3_Pb_2_I_7_	–	1.2 × 10^10^	850 μs/780 μs	–	515	–	2019	[102]
CsPbBr_3_	608	–	1.55 ms/1.77 ms	–	520	10.0	2019	[173]
(PEA)_2_SnI_4_	3.29 × 10^6^	2.06 × 10^11^	0.37 s/3.05 s	∼10	470	-5.0	2019	[214]
(C_4_H_9_NH_3_)_*n*_(CH_3_NH_3_)_*n*−1_Pb_*n*_I_3*n*+1_	38 × 10^3^	1.6 × 10^13^	∼1.7 μs/3.9 μs	∼10^4^	–	3.0	2018	[215]
CH_3_NH_3_PbI_3_	36	–	320 ms/330 ms	–	635	0.5	2017	[113]
CH_3_NH_3_PbI_3_	410	3.1 × 10^11^	86 ms/150 ms	42	405	1.0	2017	[216]
*Nanorods*								
MAPbI_3_	12.2	2.67 × 10^11^	18 ms/25 ms	800	405	4.0	2021	[217]
(CH_3_)_3_SPbI_3_	0.6	–	–	–	425	15	2021	[218]
CsPbI_3_	11.12	4.5 × 10^13^	0.57 s/0.41 s	10^5^	White light	0.0	2021	[55]
CsPbBr_3_	–	–	0.36 s/0.24 s	–	453	20.0	2021	[219]
CH_3_NH_3_PbBr_3_	1.93	1.04 × 10^12^	24 μs/103 μs	–	405	2.35	2020	[109]
FAPbI_3_	29	–	0.67 s/0.14 s	–	365	–	2019	[220]
LiCl: FAPbI_3_	167	–	0.31 s/0.14 s	–	365	–	2019	[220]
CsPbI_3_	2.92 × 10^3^	5.17 × 10^13^	0.05 ms/15 ms	–	405	2.0	2018	[123]
CsPbI_3_	4300 × 10^3^	2.2 × 10^6^		–	–	–	2018	[221]
*Nanowires*								
CsPb_2_Br_5_	206	4.07 × 10^9^	18.24/18.27 ms	–	325	1.0	2024	[222]
MAPbI_3_	(125.2 ± 2.5) × 10^3^	(2.8 ± 0.8) × 10^13^	–	< 100	650	5.0	2023	[223]
CsPbBr_3_	1.03 × 10^3^	3.53 × 10^9^	–	–	370	–	2024	[224]
(PEA)_2_PbI_4_	2.098 × 10^3^	1.752 × 10^12^	69.6/69.5 ms	–	520	-5.0	2023	[225]
(PEA)_2_PbI_4_	1.998 × 10^3^	1.669 × 10^12^	–	–	365	-5.0	2023	[225]
MAPbX_3_	35.01 × 10^3^	6.85 × 10^13^	172/114 μs		532	1.0	2024	[226]
CsPbCl_3_	49 × 10^3^	1.51 × 10^13^	–	10^4^	–	–	2024	[227]
MAPbI_3_	58.5 × 10^3^	1.96 × 10^13^	–	–	532	0.0	2024	[228]
Hexylamine/hexanoic acid- CsPbBr_3_	0.83	1.30 × 10^12^	–	–	520	1.0	2022	[128]
oleylamine/oleic acid- CsPbBr_3_	2.36	6.17 × 10^12^	–	–	520	1.0	2022	[128]
MAPbBr_3_/MAPbBr_3−*x*_I_*x*_	2.65 × 10^5^	–	170.5 ms/91.3 ms	2.4 × 10^5^	532	5.0	2022	[229]
MAPbI_3_ NWs with BMIMBF_4_	6.15 × 10^3^	4.8 × 10^12^	109 μs/502 μs	1.29 × 10^4^	320	5.0	2022	[230]
MAPbI_3_	–	–	81 ms/165 ms 13 ms/6.7 ms	–	770 730	1.0 0.0	2022	[231]
CsSnI_3_	0.237 × 10^3^	1.18 × 10^12^		–	405	–	2022	[232]
CH_3_NH_3_PbI_3_	558 × 10^3^	2.3 × 10^12^	19 ms/20 ms	10^3^	532	2.0	2022	[233]
(MTEA)_2_(MA)_2_Pb_3_I_10_/2D	7.3 × 10^6^	3.9 × 10^15^	40 μs/52.2 μs	–	530	5.0	2022	[234]
Chiral 2D perovskite	47.1 × 10^3^	1.24 × 10^13^	267 μs/258 μs	–	505	5.0	2021	[235]
(ThMA)_2_(MA)_2_Pb_3_I_10_/2D	1.1 × 10^7^	9.1 × 10^15^	36.2 μs/31.5 μs	–	530	5.0	2020	[236]
MAPbI_3_	8.52 × 10^6^	1.2 × 10^14^	350 μs/670 μs	–	660	1.0	2020	[237]
CsSnX_3_	54	3.85 × 10^–5^	83.8 ms/243.4 ms	–	940	0.1	2019	[238]
MAPbI_3_ NW arrays	12	7.3 × 10^12^	–	4.2 × 10^3^	650	0.0	2019	[239]
MAPbI_3-x_(SCN)_x_ NW	620	7.3 × 10^12^	–	–	White light	10.0	2019	[240]
(BA)_2_(MA)_3_Pb_4_I_13_/2D	1.5 × 10^7^	7 × 10^15^	27.6 μs/24.5 μs	–	515	5.0	2018	[241]
CH_3_NH_3_PbI_3_	4.95	2 × 10^13^	< 0.1 ms	–	530	1.0	2016	[242]
MAPbI_3_/3D	13.57	5.25 × 10^12^	80 μs/240 μs	–	365	- 5.0	2016	[243]
CH_3_NH_3_PbI_3_ (V_d_ = 0.2)	5	–	–	–	520	0.0	2014	[244]
*Quantum dots*								
CsPbBr_3_	304	1.17 × 10^13^	75/137 ms	–	300–1000	0.0	2024	[245]
CsPbBr_3_	1.92 × 10^7^	10^14^	–	–	405	− 0.0001	2024	[246]
CsPbI_3_	–	2.4 × 10^12^	5.2/4.1 ms	–	940	3.0	2024	[247]
MAPbBr_3_	0.19	1.58 × 10^8^	290/510 ms	40	Solar light	0.0	2024	[248]
CsPb_0.95_Zn_0.05_Br_3_	240	6.19 × 10^13^		10^7^	300–500	0.0	2024	[249]
CsPbI_3_	370	4.7 × 10^12^	43.7 ms/44.7 ms	–	605	0.0	2023	[250]
CsPbI_3_/MoS_2_	4.1 × 10^12^	4.7 × 10^6^	4.8 s/6.9 s	–	638	1.0	2023	[251]
Cs_*x*_FA_x-1_PbI_3_/MoS_2_	3.14 × 10^14^	1.6 × 10^6^	2.88 s/5.3 s	–	638	1.0	2023	[251]
CsPbI_3_/MoS_2_	2.2 × 10^13^	1.5 × 10^6^	1.25 s/1.5 s	–	638	1.0	2023	[251]
CsSnBr_3_		1.27 × 10^11^		–	365	3.0	2023	[252]
CsPbBr_3_	450 × 10 ^−3^	5.8 × 10^12^	–	–	550	0.0	2023	[253]
CsPbI_3_	–	> 10^12^	2.8 µs/-	–	300–950	3.0	2023	[254]
CsPbI_3_		10^12^	2.8 µs/-	–	638	3.0	2023	[254]
La^3+^/Yb^3+^ co-doped CsPbCl_3_	140	–		–	200–400	–	2022	[82]
CsPbBrI_2_/MoS_2_	55.81	5.20	7.7 s/-	–	254	20.0	2022	[255]
CsPbBrI_2_/MoS_2_	47.23	3.12	7.4 s/-	–	365	20.0	2022	[255]
CsPbBrI_2_/MoS_2_	1.05	1.18	0.085 s/-	–	532	20.0	2022	[255]
CsPbBr_3_	1.64 × 10^4^	3.17 × 10^12^	43.5 ms/65.9 ms	–	450	0.0	2022	[256]
CsPbBr_3_/ZnO	320	1.75 × 10^13^	–	2443	365	10.0	2022	[257]
Ti_3_C_2_T_X_ NSs/CsPbBr_3_	97 × 10^–3^	–	50 ms/20 ms	–	490	1.0	2022	[258]
Gr-CsPbBr_3_	22.8 × 10^3^	1.89 × 10^10^	–	–	266	–	2021	[259]
CH_3_NH_3_PbI_3_/Ta_2_NiSe_5_	2.4 × 10^5^	6.0 × 10^12^	12 ms/-	–	800	1.0	2021	[260]
CH_3_NH_3_PbBr_3_-rGO	1.07 × 10 ^6^	1 × 10^13^	0.3 s/0.3 s	–	442	1.0	2020	[261]
CsPbBr_3_		2.25 × 10^11^	62 ms/82 ms	–	405	0.0	2020	[262]
AgBi_2_I_7_	0.15			–	390	–	2020	[86]
CsPbBr_3_	19	10^13^	0.48 s/0.32 s	–	405	1.5	2020	[263]
CsPbBr_3_	10.1	9.35 × 10^13^	–	–	530	-1.5	2020	[264]
CsPbI_3_	105	5 × 10 ^13^	–	–	450	0.0	2019	[265]
CsPbI_3_	–	3 × 10 ^13^	–	–	700	0.0	2019	[265]
CsPbBr_3_	1.4	1 × 10^12^	48 ms/46 ms	–	520	-1.0	2019	[266]
MoS_2_/CsPbBr_3_	8	–	–	–	488	–	2019	[267]
FAPbBr_3_ QDs/Gr	1.15 × 10^8^		–	–	520	5.0	2018	[268]
CsPbI_3−*x*_Br_*x*_/MoS_2_	7.7 × 10^7^	5.6 × 10^11^	0.59 s/0.32 s	–	645	1.0	2018	[269]
MoS_2_/MAPbBr_3_	3.72 × 10^6^	≈10^11^	–	–	532	–	2018	[270]
CsPbI_3_	110 × 10^3^	2.9 × 10^13^	–	–	685	-10.0	2018	[271]
MAPbI_3_: TiO_2_ nanotubes	21 × 10 ^3^	7.8 × 10^10^	2 s/1 s	–	700	1.0	2017	[272]
*Nanocrystals*								
CsPbBr_3_	3 × 10^3^	1.2 × 10^13^		7.9 × 10^4^	405	0.0	2024	[273]
Bi_2_S_3_/CsPbBr_3_	380 × 10^–3^ 90 × 10^–3^	1.02 × 10^5^ 0.06 × 10^5^	132/65 ms 380/343 ms		532 1064	5.0	2024	[274]
CsPbBr_3_	44.5 × 10^3^	9 × 10^13^	5.0/4.6 ms	0.9 × 10^4^	470	5.0	2024	[167]
CsPbBr_3_	–	5.2 × 10^11^	180 ms/110 ms	10^4^	–	–	2024	[275]
CsPbBr_3_	2.21	2.84 × 10^9^	–	–	405	–	2023	[276]
CsPbCl_3_/MoSe_2_	117	4.9 × 10^11^	–	10^3.8^	1064	5.0	2023	[277]
MAPbBr_3_	3.06 × 10 ^3^ 1700	2.28 × 10^11^	43.6 ms/33.7 ms	10^5^ 10^6^	Solar Irradiation	- 3.0	2023	[278]
CsPbI_3_	370	4.7 × 10^12^	43.7 ms/44.8 ms	–	605	0.0	2023	[250]
MAPbBr_3_	97.5	10^12^	28 ms/32 ms	10^4^	520	0.0	2022	[279]
PbSe:CsPbBr_1.5_I_1.5_	616	5.96 × 10^13^	260 ms/289 ms	10^5^	405	–	2022	[81]
CsPbBr_1.5_I_1.5_	6160	–	350 ms/375 ms	–	532	–	2022	[81]
MAPb(I_1–x_Br_x_)_3_	331	4.27 × 10^10^	180 µs/200 µs/	–	743	0.0	2021	[280]
CsPbBr_3_/Cs_4_PbBr_6_	94	4.2 × 10^12^	10.85 ms/2.25 ms	10^5^	375	1.0	2021	[281]
CsPbBr_3_	100	4.2 × 10^12^	13 µs/28 µs	10^6^	473	0.01	2021	[282]
MAPbI_3_	160	7.34 × 10^11^	150 ms/50 ms	10^4^	400	0.0	2021	[283]
Cs_3_Bi_2_I_9-x_Br_x_	15	4.6 × 10^11^	40.7 ms/27.1 ms	10^4^	375	0.0	2020	[284]
MAPbBr_3_	85,000	≈10^12^	0.09 s/0.11 s	10^2^	Solar irradiation	–	2020	[285]
Cs_3_Bi_2_I_9-x_Br_x_	15	4.6 × 10^11^	40.7 ms/27.1 ms	10^4^	375	0.0	2020	[284]
MAPbI_3_	451	1.1 × 10^11^	200 ms/200 ms	–	720	0.5	2019	[286]
EA_4_Pb_3_C_l10_	0.0262	3.06 × 10^9^	0.8 s/0.22 s	10^4^	266	0.0	2019	[287]
CsPbCl_3_	1890	–	41 ms/43 ms	10^3^	356	5.0	2017	[288]
MAPbBr_3_/MAPbI_x_Br_3-x_	11.5	–	2.3 s/2.76 s	–	450	0.0	2016	[289]
CsPbBr_3_	180	6.1 × 10^10^	1.8 ms/1.0 ms	10^3^	442	10.0	2016	[290]
CsPbI_3_	–	–	24 ms/29 ms	10^5^	405	1.0	2016	[291]
MAPbI_3_	46.9	1.2 × 10^10^	24 ms/62 ms	10^3^	356	15.0	2015	[292]

## Perovskite Photodetectors: Degradation Mechanisms and Long-Term Stability Solutions

**Fig. 2:**
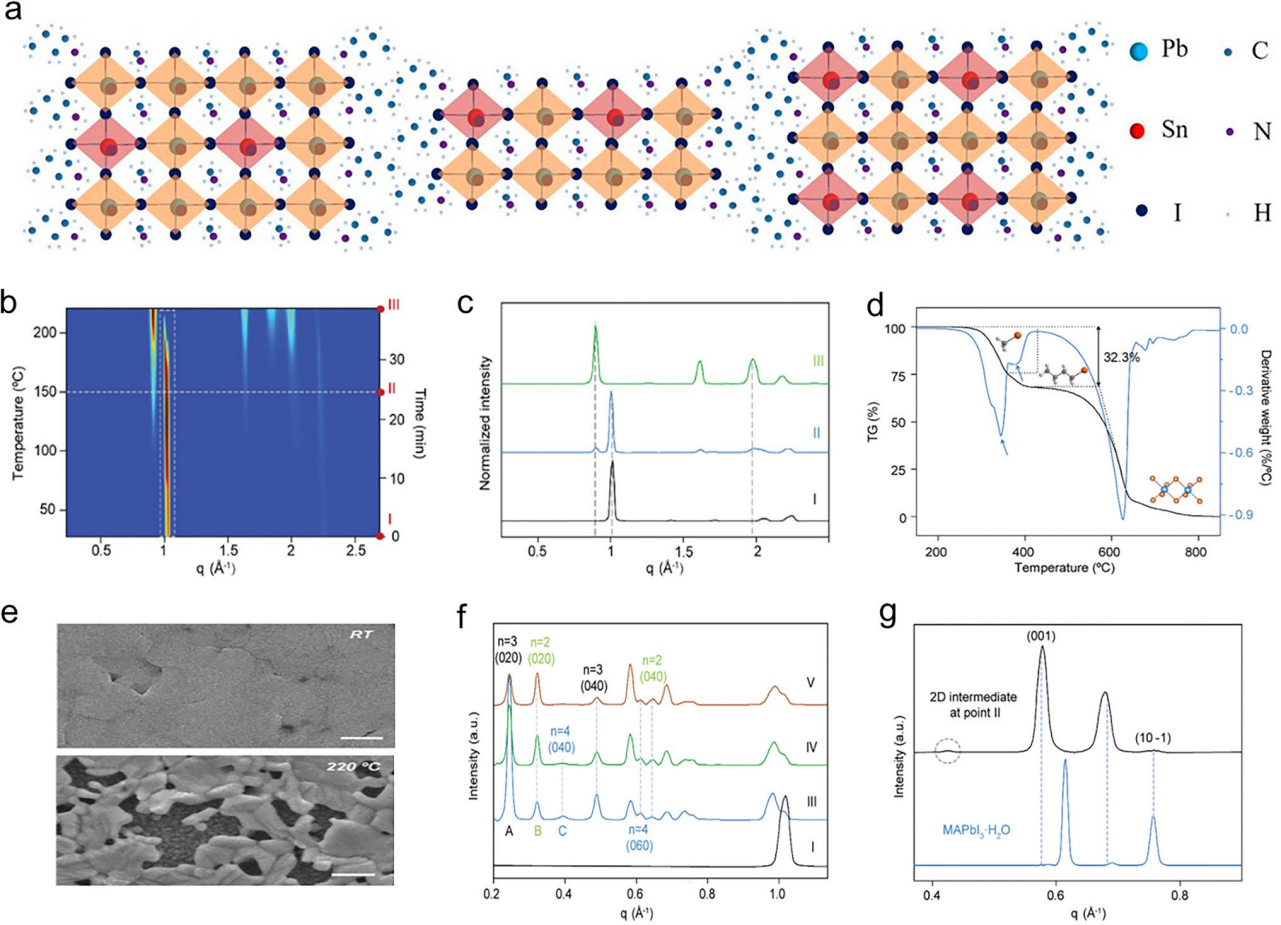
**a** Crystal structures of the low-dimensional perovskite. Reproduce with permission [294]. Copyright 2018, ACS. **b** Temperature ramping profile-based 2D intensity–time colour mapping that tracks the low-dimensional perovskite film degrading process. **c** Low-dimensional film’s typical 1D XRD patterns. **d** Blue line represents the derivative of the TG curve in the low-dimensional perovskite powder’s TG analysis and MS results. **e** Tracking of morphology with SEM analysis at RT and 220 °C, respectively (the scale bars represent 500 nm). **f** Low-dimensional perovskite film’s 1D GI-XRD patterns were obtained at positions. **g** The MAPbI_3_-H_2_O (blue curve) and the sample’s 1D XRD patterns. **b-g**. Reproduce with permission [295]. Copyright 2021, RSC

The type of perovskite material used determines the degradation mechanisms of nanoscale perovskite PDs. Lead-based 3D perovskites have good optoelectronic characteristics but are very sensitive to light, moisture, heat instability, and oxygen, which can cause oxidation and structural breakdown [293]. Even though lead-free perovskites were created to lessen toxicity concerns, they perform worse and are more susceptible to temperature, moisture, and oxygen. Low-dimensional perovskites (0D, 1D, and 2D) can provide better stability; for example, 2D perovskites have an advantage in moisture resistance due to hydrophobic organic spacers; however, 0D perovskites are still in the early stages of research and may present unique degradation challenges. The general chemical formula of the low-dimensional perovskites species is (*LOC*)_*m*_(A)_*n*−1_B_*n*_X_3*n*+1_, where n is the layer thickness of corresponding perovskites, and *LOC* is the larger organic cation. Crystal structures of the low-dimensional Pb–Sn-based halide perovskites were displayed in Fig. 2a [294]. The exceptional environmental stability of long-chain organic molecules and the excellent optoelectronic properties of perovskite materials are combined in an alternate stacking arrangement of the octahedral perovskite slabs and the long-chain organic cations. The ideal shape and crystal orientation of low-dimensional perovskites can be achieved by various fabrication methods/techniques; however, the perovskites’ degrading behaviour is still debatable. Meng et al. [295] thoroughly analysed the low-dimensional perovskites to clarify the thermal and humidity degradation mechanism of low-dimensional perovskites. Using the in situ grazing-incidence X-ray diffraction (GI-XRD) technique, the thermal degradation process of the low-dimensional perovskite is observed under heat stress. The 2D intensity–time colour mapping is produced by integrating the 2D GI-XRD images taken after deterioration and plotting in the time (temperature) domain. This naturally illustrates how the low-dimensional film’s structure changed during annealing. The typical 1D XRD curves taken at the sites I–III (denoted in Fig. 2b) are presented in Fig. 2c. The steady lowering of the major perovskite diffraction peak at *q* approximately 1 Å^−1^ demonstrates the degradation of the low-dimensional layer. Around 75 °C is when the PbI_2_ diffraction peaks appear, and around 210 °C, the transition from perovskite to PbI_2_ is complete. The primary peak becomes a single peak pattern above 200 °C, signifying the complete breakdown of the 3D-like perovskite species. For the thermogravimetric (TG) measurement, the powder scraped off the low-dimensional films (about 20 mg) is utilized, and the liberated gases are gathered for the mass spectrometry analysis. The TG curve obtained during the low-dimensional perovskite powder’s thermal degrading process is displayed in Fig. 2d. The breakdown of the organic component related to MA/BA and the inorganic PbI_2_ may be linked to two apparent mass-loss stages. The stoichiometric fraction of the MA/BA halides in the nominal composition of BA_2_MA_3_Pb_4_I_13_ shows a nice correlation with the 32.3% weight loss in the first phase. Using this method, earlier research showed that the reverse Menshutkin reaction between the MA and iodide ions produced the thermodynamically favourable pathway for the MAPbI_3_ degradation, releasing CH_3_I and NH_3_ [296, 297]. These peaks’ appearance suggests that the low-dimensional perovskite powder produces CH_3_I and NH_3_ during thermal decomposition. The morphological evolution is further examined using scanning electron microscopy (SEM) to trace the low-dimensional perovskite heat breakdown channels, as shown in Fig. 2e. At 220 °C, the low-dimensional film breaks down into separate PbI_2_ phases. GI-XRD and TG also confirmed this result. Furthermore, they examined how the humidity causes the low-dimensional perovskite film to deteriorate. In order to provide information under high-humidity conditions, the film was mounted in a specially designed cell that was alternately purged with water vapour (relative humidity (RH) > 95%). Figure 2f displays the typical XRD pattern obtained with *n* values of 2, 3, and 4. The principal diffraction peaks may be independently indexed into the pure-phase 2D perovskite species. The film’s orientation also drastically alters in contrast to the original low-dimensional perovskite crystals, which were oriented vertically. The regenerated perovskite species had a random orientation, with most of their crystals aligned parallel to the substrate. Four distinct diffraction peaks, with respective centres at q of 0.42, 0.58, 0.68, and 0.76 Å^−1^, are observed in the 2D intermediate (Fig. 2g). Since the experimental XRD pattern of MAPbI_3_**·**H_2_O is acquired by tracking the hydration process of the MAPbI_3_ film, it is possible to assign the peaks at 0.58, 0.68, and 0.76 Å^−1^ to MAPbI_3_**·**H_2_O, indicating that the 2D intermediate is related to a hydrated species. One can attribute the undesignated peak at 0.42 Å^−1^ to either BAI or its hydrate. Moreover, single-crystal perovskites generally exhibit superior stability compared to their polycrystalline counterparts due to their lower defect densities and increased carrier mobility. However, producing high-performing single-crystal arrays is still a major challenge. New thin-film materials are often used in modern technologies, such as integrated circuits and optoelectronic devices. This precision makes using different materials in integrated photonics, high-efficiency photovoltaics (PV), LEDs, and PDs possible [298]. Even so, perovskite-based materials have many obstacles in their path to long-term sustainability and durability [76, 299]. Perovskite-based devices must endure harsh environmental factors like high moisture, oxygen, light, and temperature to be used sustainably. They must also overcome intrinsic problems like non-radiative recombination, hysteresis, and grain boundaries. One drawback of using low-dimensional perovskites in optoelectronic devices is that their enormous surface area results in a high density of electronic surface trap states despite low-dimensional perovskites lowered grain boundaries and interfaces for efficient photocarrier transport. Furthermore, when exposed to moisture or light, the dangling connections may serve as sites for material degradation reactions, considerably reducing the stability of the device [27].

**Fig. 3:**
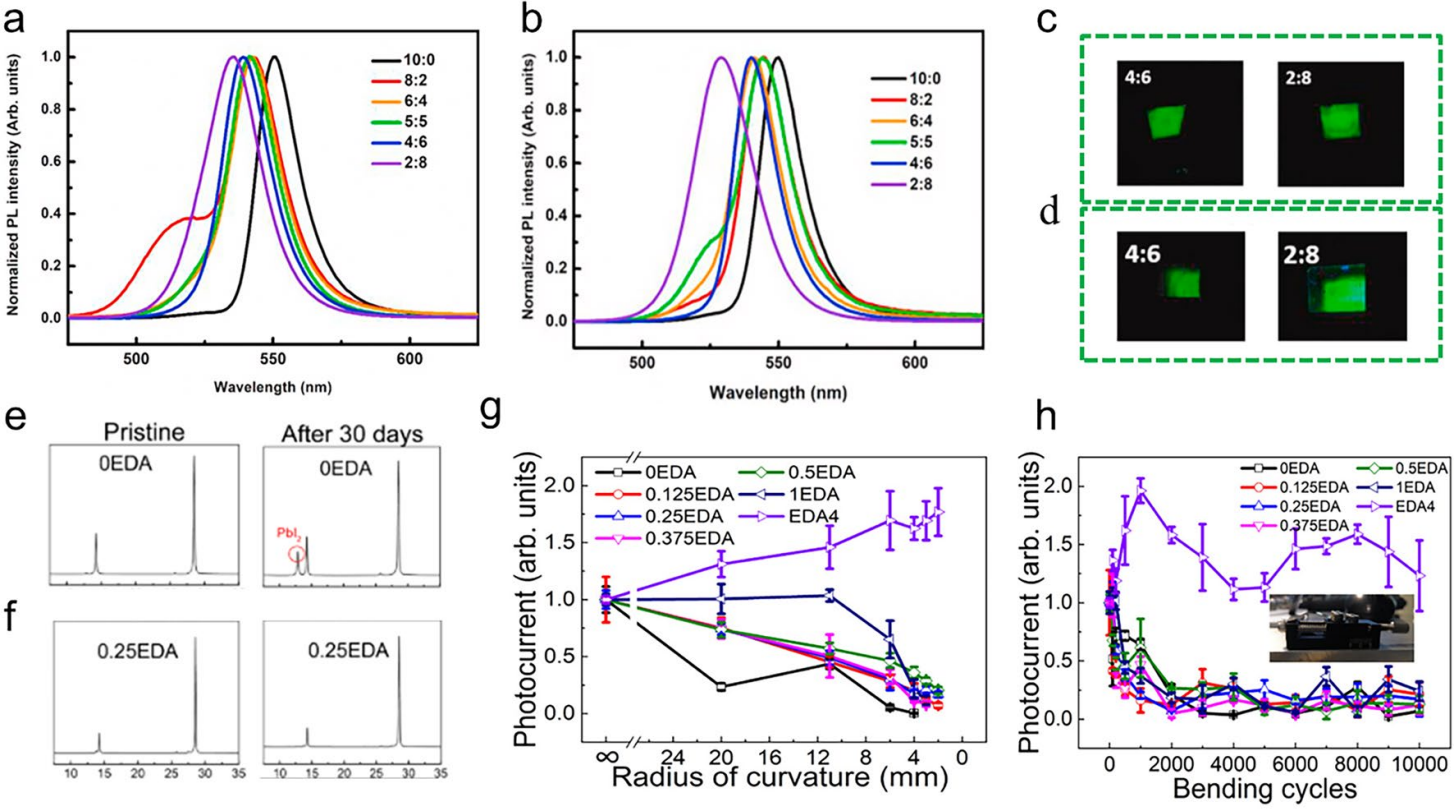
**a** PL emission spectra of films produced with varying lead-to-tin ratios. **b** Three months later, the same samples’ PL spectra. **c** Under ultraviolet light, images of as-prepared films with a lead-to-tin ratio of 4:6 and 2:8. **d** Film images following a three-month prep period. **a**–**d** Reproduce with permission [300]. Copyright 2021, Elsevier. XRD patterns of **e** control perovskite and **f** EDA-doped perovskite following ambient storage for 30 days at 60% humidity. **g** Normalized photocurrent of flexible devices—which includes the EDA4 and other devices—depends on their bending curvature. **h** How the normalized photocurrent varies with each flexible device’s bending cycle. The optical view of the measuring set-up during the bending test is displayed in the inset. **e–h** Reproduce with permission [305]. Copyright 2020, ACS

Several approaches have been proposed to address the degradation mechanisms in perovskite-based PDs [293]. These include compositional engineering, which aims to improve the inherent stability of materials; hydrophobic barrier packaging, which shields against environmental factors; and surface passivation. The low-dimensional perovskites may be replaced by layered perovskites, which can address device stability concerns. Through compositional engineering, Vijila et al. [300] presented the synthesis of ultrastable and highly luminous mixed lead–tin (Pb–Sn) 2D and NW perovskite structures. The as-prepared mixed Pb–Sn bromide perovskites’ PL spectra were displayed in Fig. 3a, where an increase in Sn content causes the emission peak to shift blue. Oleylamine, introduced as a long-chain ligand to control the size in this specific synthesis procedure, can address the rise in the band gap of mixed Pb–Sn perovskites. After three months, samples like 2:8 have weaker emission from the phase with the lowest band gap, making the shoulder peak more prominent. Remarkably stable and bright were the 4:6 and 2:8 (Fig. 3b). Also, images of the films 4:6 and 2:8 immediately after processing and three months later were shown in Fig. 3c, d. While there is no serious loss of brightness in the film with a ratio of 4:6, there are brightness losses in the samples with other ratios under UV illumination. More importantly, after three months, the photoluminescence quantum yield (PLQY) for the 4:6 ratio decreased by only 3.6%, while the PLQY ratio for all films in other ratios decreased by up to 90%. More durable lead-free substitutes are also being developed, though their functionality needs improvement. Ensuring long-term durability is still a difficult challenge for commercial applications, even with advancements in stability. Current research efforts to address these degradation problems focus on improving fabrication processes, refining materials, and optimizing device structures.

Reduced non-radiative recombination losses are essential for perovskite-based devices to function at their best and remain stable over time. These losses come from various sources, including optical losses in the perovskite layer, energy-level mismatches, intrinsic defect-assisted recombination, and interface-induced recombination. These losses affect charge extraction efficiency and change the characteristics of the device [171]. Interface engineering is a useful strategy to reduce interfacial losses while maintaining bulk layer properties [301]. This technique modifies interface strategies to improve optoelectronic properties, which increases overall device stability and boosts device performance. Applying interfacial layers within the structure acts as a barrier to protect perovskite films, affecting the rate at which water diffuses and the transportation of degradation by-products [302].

Considerable attention has been focused on improving perovskites for devices using novel dopant materials. In order to produce films and devices with better performance and stability than reference materials, doping strategies entail adding additives to the device structure [303, 304]. The nucleation and growth processes of perovskite can be carefully controlled to produce larger grains, more uniform surfaces, and higher crystallinity, which significantly impact device performance. Additionally, by reducing grain boundaries, these advancements increase carrier mobility, lengthen carrier diffusion lengths, and prolong charge lifetimes in perovskite-based devices. With the doping strategy of the special bication ethylenediammonium (EDA), high-quality quasi-2D halide perovskite thin films are effectively produced using a one-step spin coating technique [305]. By replacing the conventionally large and weakly van der Waals-interacted organic bilayer spacer cations that produce the unique Dion–Jacobson phase, these EDA molecules, which have a short alkyl chain length, can improve the phase stability and mechanical flexibility of low-dimensional perovskite. Structural analyses revealed the benefits of doping engineering on the long-term stability behaviour of perovskite films under high humidity (Fig. 3e, f). The relatively strong moisture stability of the manufactured low-dimensional perovskite was demonstrated by the XRD spectra of all samples, which show no discernible changes after 7 days of ambient storage at 60% humidity. However, the control XRD pattern exhibited that the samples degraded somewhat after 30 days. At this point, a clear PbI_2_ peak was seen. On the other hand, the XRD spectra of the EDA-doped perovskite sample stay the same, indicating that EDA can stabilize the crystal structure. Their study also involves the fabrication of EDA-based low-dimensional perovskites with *n* = 4 on polyimide substrates to create flexible PDs. Analysing the variation in photocurrent with each manufactured device’s bending radius is crucial for the performance evaluation (Fig. 3g). An optical image of the measurement set-up is shown in Fig. 3h, inset to show precise control of the bending radius. When the control device was bent to a radius of 20 mm, the photocurrent rapidly dropped to 23.5% of its starting value, as observed by the normalized photocurrent. However, the devices with low EDA content (i.e. 0.125 ≤  *x* ≤ 0.5) stay at over 73.5%, and the photocurrent of the 1EDA device even stays at 100% with the same bending magnitude. More surprisingly, it displayed that the photocurrent of EDA4 progressively rises to 177% once the devices are finally bent down to a radius of 2 mm. The doping strategy must further enhance the moisture stability of the produced low-dimensional perovskite films. All of these findings can offer a useful framework for creating the best low-dimensional perovskite films for mechanical, flexible, high-performing, air-stable, next-generation optoelectronic devices. All these benefits work together to improve perovskite-based devices’ overall efficiency and stability. Therefore, stable and highly efficient perovskite-based devices depend on minimizing grain boundaries and optimizing perovskite crystal size.

## Ligand Selection and Morphology’s Impact on Nanoscale Perovskites

In order to control the morphology of nanoscale perovskites, which greatly affects their optical and electrical properties, ligand selection and experimental set-up are critical. For instance, choosing the right ligand is crucial because different ligands can produce different shapes for the nanoscale perovskites [306]. For instance, the morphology of CsPbBr_3_ NCs can be controlled to produce unique shapes using alkene-derived zwitterionic ligands, and the nucleation, growth, and stability of perovskite NCs can be influenced by oleic acid and oleylamine [307]. Morphology is also determined by the solvent used; different solvents can form diverse nanoscale perovskites (NWs, NRs, or QDs) [308]. Another important consideration is reaction time, which affects how various NC shapes evolve sequentially [308]. Furthermore, the reaction temperature influences the self-assembly and photoluminescence of caesium lead halide perovskite NCs [178]. Finally, the form and thickness of the NCs in flow synthesis can be controlled by the type and degree of mixing during the growth and nucleation stages.

**Fig. 4:**
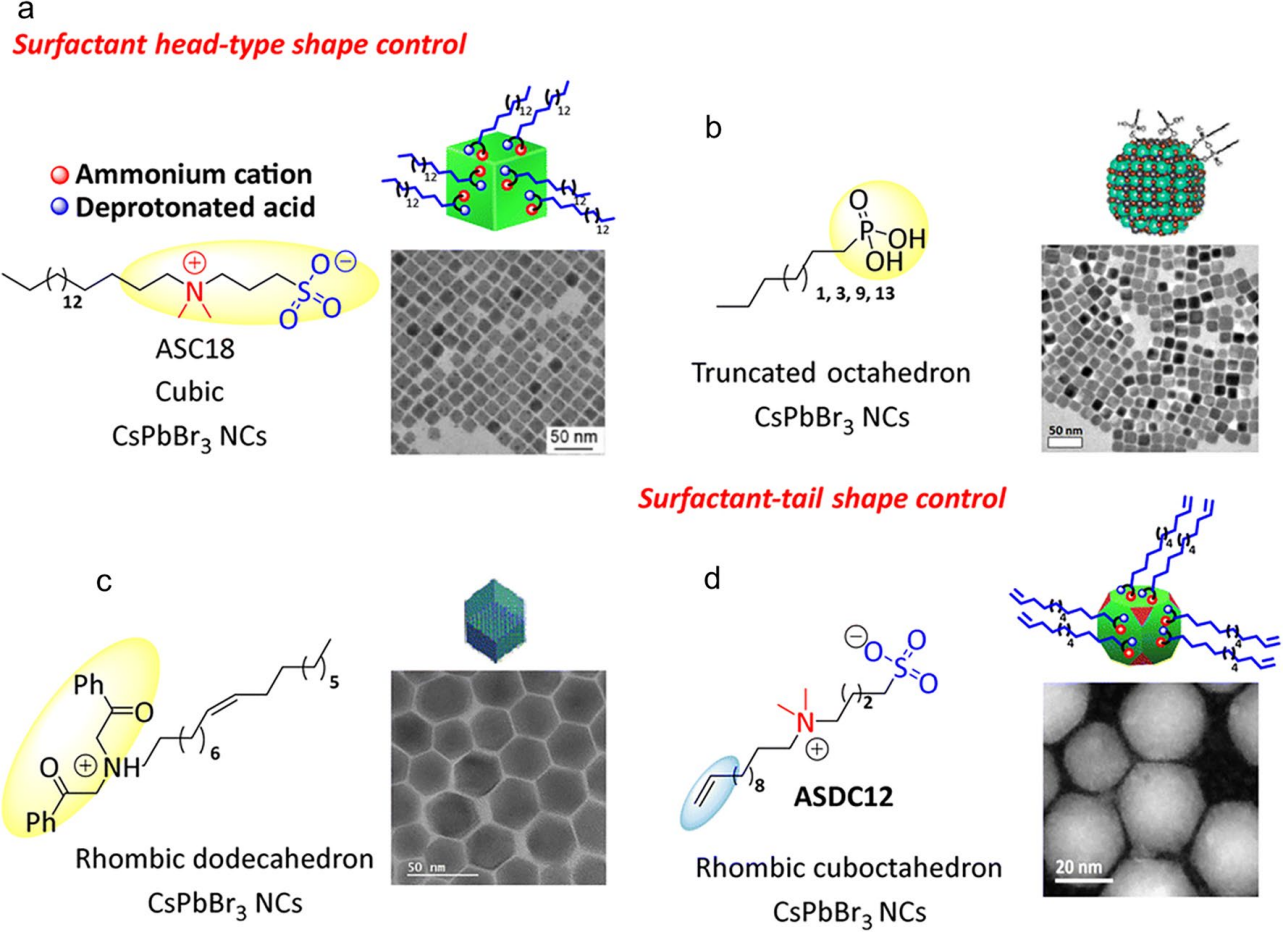
Visual representation of the ligands used to synthesize CsPbBr_3_ NCs and the associated NC morphologies with **a** surface head type shape control, **b** truncated octahedron, **c** rhombic dodecahedron-shaped, **d** rhombicuboctahedron shape. Adapted with permission [306]. Copyright 2024, RSC

Many strategies have been used, such as utilizing low-polarity antisolvents (like methyl acetate) to eliminate unreacted precursors and adjusting the ligand equilibrium with excess ligands (OA/OAm) to stop ligand desorption during isolation [306, 309]. The first use of colloidal CsPbBr_3_ NCs capped with zwitterionic ligands as surface stabilizing agents was recently reported by Franziska et al. [310]. The highest colloidal NC surface stability was demonstrated by cubic CsPbBr_3_ NCs capped with 3-(*N*,*N*-dimethyloctadecylammonio)-propanesulphonate (ASC18) sulphobetaine, which made material isolation simpler. The chelate effect between the corresponding ammonium cation and the deprotonated acid of the ligand on the NC surface is responsible for this stability (Fig. 4a). Moreover, the cubic shapes of CsPbBr_3_ NCs capped with the zwitterionic ASC18 ligand and OAm/OA remain intact. Although most perovskite NCs have a cubic morphology, recent research has demonstrated that lead bromide perovskite NCs’ intrinsic optical and catalytic properties can be precisely tuned by carefully regulating facet growth and shape [311]. Considerable synthetic efforts have been made in this context to alter the shape of CsPbBr_3_ NCs. These attempts include the addition of additional metals like Cd and Mn [312], lowering the injection temperature, modifying Cs_4_PbBr_6_ NCs post-synthesis [313], or using short-chain amines in place of widely used long-chain ligands [314]. A noteworthy instance is the work of Baowei et al. [315], which synthesized highly stabilized colloidal CsPbBr_3_ NCs with a truncated octahedron morphology by using capping ligands, alkyl-phosphonic acids to passivate the NC surface (Fig. 4b). Due to their strong affinity for the NC surface, the phosphonate ligands stabilized the {001} and {110} NC facets [315]. Furthermore, Bera et al. [316] reported rhombic dodecahedron-shaped CsPbBr_3_ NCs using α-bromoketone as a capping ligand. Tertiary ammonium ions, produced by sequential *S*_*N*_2 reactions of the corresponding bromoketone with OAm, stabilized the resulting quasi-spherical particles. These tertiary cations successfully passivated the {200}, {020}, and {112} facets of CsPbBr_3_ NCs (Fig. 4c). Surprisingly, these techniques use the ligand’s "head part" to modify how NC morphology evolves. As far as we know, this is the first time that the capping ligand’s terminal functional group can control the growth of facet NCs. This method is useful because it allows the functional characteristics that are unique to these "heads," like those in phosphonic acids or zwitterionic ligands, to be preserved. Furthermore, although there are a finite number of "heads" that can engage with the NC surface, there may be an infinite number of "tails" that can be investigated and employed. In another report, Yoarhy et al. [177] proposed the use of ligands with terminal functional groups as a remote regulator for the facet formation of CsPbBr_3_ NCs. In particular, the ligand—a zwitterionic ligand—integrates a terminal double bond that encourages consistent ligand ordering on the nanocrystal surface with a head group that can effectively stabilize the surface (sulphobetaine). Surprisingly, the rare rhombicuboctahedron (SRO)-shaped CsPbBr_3_ NCs were formed when the alkene moiety was added to the surfactant tail (Fig. 4d).

Nanoscale perovskites’ optical and electrical characteristics are significantly influenced by their morphology. For example, strongly confined perovskite QDs with diameters less than 7 nm exhibit notable quantum confinement effects, which lead to different optical properties from larger NCs [317]. NCs’ emission wavelength can be greatly influenced by their size and shape; for example, CsPbBr_3_ nanoplatelets with a 2.2 ± 0.3 nm thickness emit at 472 nm [317]. Furthermore, different morphologies can result in different photoluminescence quantum yields (PLQY); high-quality NCs of different sizes and shapes display excellent crystalline quality and high fluorescence quantum yield [308]. The optical and electrical properties of NCs with high surface-to-volume ratios are more vulnerable to ligand interactions and surface defects [318]. Perovskite NC morphology also affects charge carrier mobility, which is important for its functionality in optoelectronic devices [318]. Another important component is stability, which can vary widely among morphologies and is necessary for devices to function well over extended periods [307]. Researchers can engineer nanoscale perovskites with specific morphologies tailored for desired optical and electrical properties by carefully choosing ligands and managing experimental conditions [308]. This degree of control is essential to optimize these materials for various uses, including light-emitting diodes, photovoltaics, and other optoelectronic devices.

## Perovskite-Based Nanosheets

### Growth of Perovskite-Based Crystalline Nanosheets

Recent studies have outlined several difficulties in the development of crystalline perovskite NSs. Unlike the direct growth of perovskite single crystals, a major challenge is fabricating large-sized LHP NSs from single crystals [47]. Furthermore, the synthesis of organic–inorganic LHP single crystals can face growth and structural stability difficulties if thin single crystals at micro-/nanosizes can be achieved [49]. Perovskite materials, including NSs, have been identified as having challenges in their growth and application, including structural stability, device fabrication, longevity, cost-effectiveness, recombination, and optical properties [44, 319, 320]. These difficulties highlight how difficult it is to improve the synthesis and application of perovskite crystalline NSs in optoelectronic devices and other applications by addressing several factors. Recent research has demonstrated various methods that can be employed to synthesize crystalline NSs of perovskite. A study concentrated on synthesizing free-standing, centimetre-sized perovskite NSs for optoelectronic devices using single-crystal lead bromide; growth happened along two in-plane directions without appreciable thickness increase [47]. Furthermore, various applications have been investigated for the growth of organic–inorganic LHP single crystals, including thin single crystals at micro-/nanosizes [49]. These investigations aid in understanding the synthesis, characteristics, and uses of perovskite single crystals and NSs in optoelectronic devices [48, 319].

**Fig. 5:**
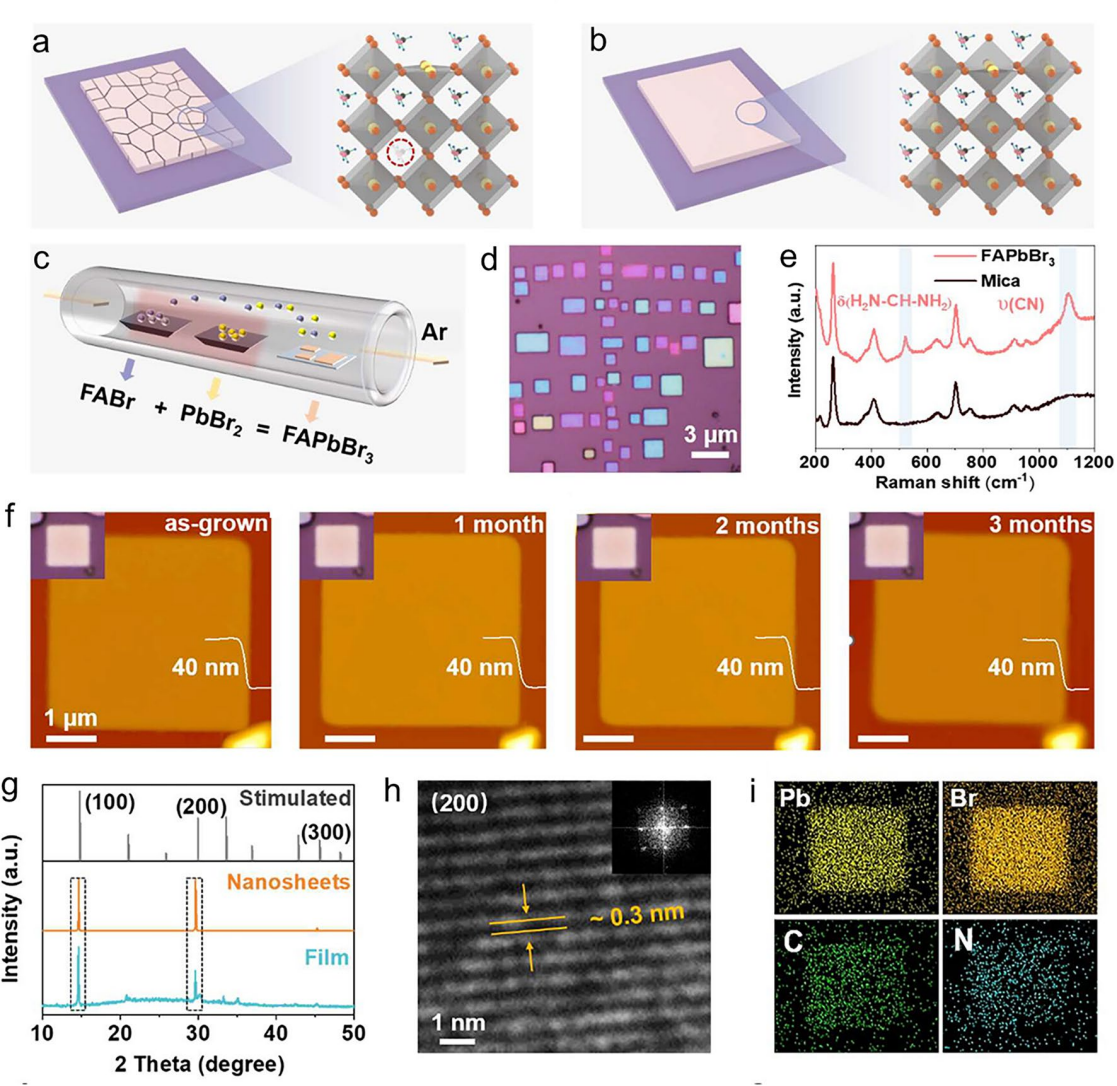
**a, b** Diagram showing FAPbBr_3_ polycrystalline films and single-crystalline NSs, respectively. **c** A diagram illustrating the FAPbBr_3_ NSs’ one-step CVD growth process. **d** A schematic of FAPbBr_3_ NSs using optical microscopy. **e** FAPbBr_3_ NSs’ Raman spectra are on a mica substrate and by themselves. **f** Images from optical microscopy (with insets) and matching AFM images of a FAPbBr_3_ NS exposed to different air exposure times. **g** XRD patterns of polycrystalline films (blue line) and FAPbBr_3_ NSs (orange line), respectively. The data are standardized. The simulated XRD pattern of FAPbBr_3_ (grey line) is offered for comparison. **h** FFT image in the inset and HRTEM image of FAPbBr_3_ NSs. **i** Using energy- EDS, Elements Pb, Br, C, and N elemental mappings of a FAPbBr_3_ NS. **a**–**i** Reproduce with permission [44]. Copyright 2024, ACS

The intriguing optoelectronic characteristics of organic–inorganic hybrid perovskites have attracted much attention. However, their poor air stability greatly hinders their usefulness, particularly as their thickness approaches the nanometre. Shi et al. [44] reported a single-step vapour-phase method for growing HC(NH_2_)_2_PbBr_3_ (FAPbBr_3_) single-crystalline NSs with tunable sizes up to 50 μm and thicknesses as low as 20 nm. Even after months of exposure to air, the FAPbBr_3_ NSs show remarkable stability, retaining their photoluminescence (PL) efficiency and surface roughness without any degradation. The structures of single crystals and polycrystalline films of FAPbBr_3_ are shown in Fig. 5a, b. The [PbBr_6_]^4–^octahedra in FAPbBr_3_ share corners to form a three-dimensional network, and as Fig. 5b illustrates, FA^+^ cations occupy the coordinated holes [321]. Compared to single crystals, polycrystalline films have many surface defects and grain boundaries (Fig. 5a), reducing perovskite stability in ambient environments [322, 323]. It is possible to efficiently grow FAPbBr_3_ single-crystalline NSs by a one-step (CVD) method. The FAPbBr_3_ NSs’ growth process is depicted in Fig. 5c. A mixture of FABr and PbBr_2_ (1:1 molar ratio) in the middle of the furnace, with a mica substrate piece downstream. The reactants are transported by high-purity argon to the mica substrates for nucleation and growth. The synthesis of FAPbBr_3_ polycrystalline films is carried out by spin coating. An optical microscopy (OM) image of FAPbBr_3_ NSs with square morphologies and regular edges is shown in Fig. 5d. X-ray photoelectron spectroscopy (XPS), high-resolution TEM (HRTEM), X-ray diffraction (XRD), energy-dispersive spectrometry (EDS), and Raman spectra are used to analyse the structure and composition of FAPbBr_3_ NSs that are synthesized. The Raman spectra of FAPbBr_3_ NSs grown on mica are shown in Fig. 5e. The bending modes of H_2_N–C–NH_2_ are responsible for the peak at 521 cm^–1^, whereas symmetric stretching C–N modes are responsible for the peak at 1120 cm^–1^ [324, 325]. Each other peak is indexed to the mica substrate. The synthesized NSs are exposed to air for varied periods at a temperature of 25 °C and a relative humidity (RH) of 55% to assess their stability. Over 3 months, the NSs’ morphology and surface roughness are essentially unchanged (Fig. 5f). The XRD patterns of the polycrystalline films and FAPbBr_3_ NSs are shown in Fig. 5g. In order to reduce the impact of the highly intense XRD mica substrate, the NSs have been moved to Si/SiO_2_ substrates [326]. The (100), (200), and (300) planes of cubic FAPbBr_3_ with space group Pm-3 m are responsible for three prominent diffraction peaks of the resultant NSs (Fig. 5g, orange line) [323]. The simulated FAPbBr_3_ diffraction peaks and those seen in the synthesized polycrystalline films agree (Fig. 5g, blue line). Lattice spacings of roughly 3 Å are found in HRTEM studies, which correspond to the (200) planes of cubic FAPbBr_3_ (Fig. 5h). Using a single set of diffraction spots, the fast Fourier transformation (FFT) of the grown NS (Fig. 5i) further confirms the nature of the single-crystal structure. Pb, Br, C, and N elements show a uniform spatial distribution, according to EDS mappings. With this method, the difficulties posed by hybrid perovskites in integrated optoelectronics can be overcome, and the requirements for exceptional performance, high stability, and nanoscale thickness can be satisfied.

### Perovskite Nanosheet-Based Photodetectors

**Fig. 6:**
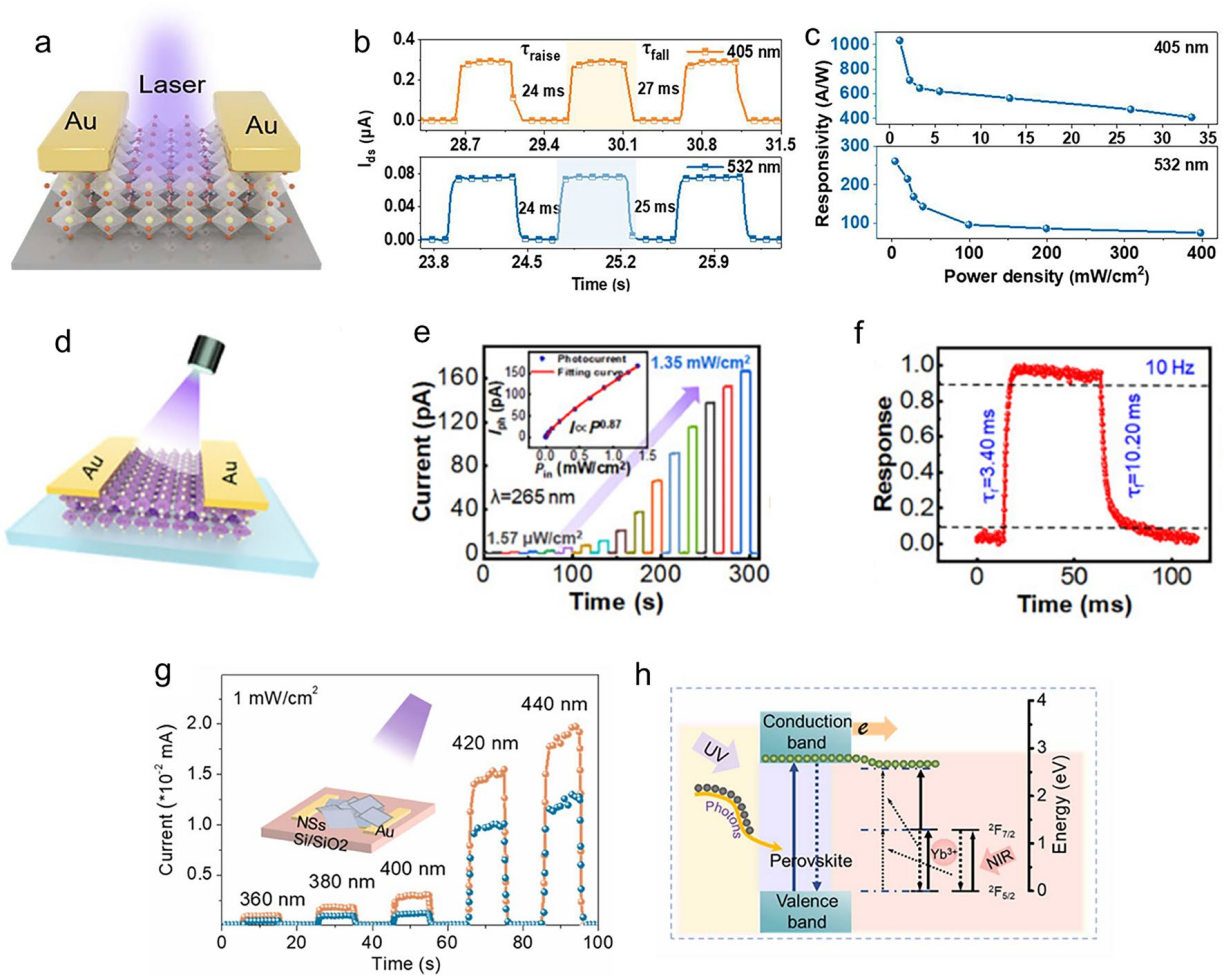
**a** Diagram illustrating a FAPbBr_3_ PD. **b** PD’s time-resolved photoresponse when excited by 405 and 532 nm lasers at *V*_ds_ = 3 V. **c** Responsivity values computed at *V*_ds_ = 3 V while 405 and 532 nm lasers are excited. **a**–**c** Reproduce with permission [44]. Copyright 2024, ACS. **d** Diagram showing an example of a DUV PD for CsPbBr_3_. **e** Device’s time-dependent photoresponse to varying incident light intensities at 265 nm. The photocurrent as a function of light intensity is shown in the inset. **f** To find the rise/fall time, a single magnified response cycle recorded at 10 Hz was used. **d**–**f** Reproduce with permission [207]. Copyright 2023, Elsevier. **g** Photocurrent of Yb^3+^-doped and undoped NSs devices under various UV light wavelengths, respectively. **h** Potential mechanism of Yb^3+^-doped CsPb(Cl/Br)_3_’s 980 nm response. **g**, **h** Reproduce with permission [208]. Copyright 2023, Elsevier

Perovskite NSs have shown great promise in PDs, as studies have demonstrated how well they can improve photodetection performance. CsPb_2_Br_5_ NSs, for example, have been used to create PDs with exceptional switching current ratios and quick response times [53]. Furthermore, studies have explored using SnS_2_ NSs to create stable planar PD with quick response times [107]. Additionally, developments in organic–inorganic hybrid perovskite-based PDs and their improved designs have been highlighted in perovskite-based PDs [52]. These results highlight how perovskite NSs can improve PDs’ performance. A vapour-phase growth of HC(NH_2_)_2_PbBr_3_ (FAPbBr_3_) single-crystalline NSs with tunable sizes up to 50 μm and thickness was reported [44]. They developed PDs to investigate the optoelectronic properties of FAPbBr_3_ NSs. The PD’s device structure, which depends on a single FAPbBr_3_ NS, is shown in Fig. 6a. The time-resolved photoresponses at *V*_ds_ = 3 V for 405 nm with 5.5 mW cm^–2^ and 532 nm with 20 mW cm^–2^ are shown in Fig. 6b. For the 405 and 532 nm cases at *V*_ds_ = 3 V, the PDs show high light current to dark current values (on/off ratio) of roughly 10^4^. The sensitivity values obtained with 405 and 532 nm lasers excited at *V*_ds_ = 3 V are displayed in Fig. 6c. The results indicate a high on/off ratio of 10^4^, an EQE greater than 3000%, an ultrahigh responsivity of 1033 A W^−1^, and a fast response time of roughly 25 ms. In another report, Chun-Yan et al. [207] reported using room-temperature, confined-space grown CsPbBr_3_ NSs to fabricate a highly sensitive deep ultraviolet (DUV) PD. A representative PD based on the 68 nm CsPbBr_3_ NS is schematically shown in Fig. 6d. The device’s photoresponse to illumination at 265 nm with varying light intensities is shown in Fig. 6e. It is evident that when the light intensity increases from 1.57 µW cm^−2^ to 1.35 mW cm^−2^, the photocurrent increases gradually from 0.53 to 167 pA. Stronger illumination intensities produce more carriers, which raises the photocurrent, so this observation makes sense [327]. *θ* is fitted to be about 0.87, as shown in the inset of Fig. 6e. The device may have recombination loss as indicated by the deviation from the integer. Nonetheless, the *θ* value is significantly greater than the CsPbBr_3_ thin film that was previously reported (*θ* = 0.67) [327], suggesting a respectable level of crystallinity. The rise/fall time *τ*_r_/*τ*_f_, which is the amount of time needed for the photocurrent to increase from 10% to 90% or decrease from 90% to 10% of the maximum, is estimated to be 3.40/10.20 ms based on a single magnified response cycle measured at 10 Hz, as shown in Fig. 6f. This implies that all-inorganic perovskites are potential candidates for future DUV PDs that are sensitive and reasonably priced. The exceptional optical and electrical properties of 2D all-inorganic halide perovskites have piqued current research interest. Doping with rare earth ions is a promising method for optimizing their optical and electrical properties and enabling a wide range of applications. Nevertheless, there are few reports on 2D RE ion-doped perovskite crystals in the literature. Novel 2D all-inorganic perovskite CsPbCl_*x*_Br_3−*x*_ NSs doped with rare earth ions were synthesized, as reported by Sun et al. [208]. As shown in the inset of Fig. 6g, the planar device structure based on the lateral assembly of 2D perovskite NSs was selected to create PDs. This choice was taken in light of planar structures’ superior electrical qualities. When operating in photovoltaic mode, the device doped with Yb^3+^ (Fig. 6g) shows enhanced photoresponses to light ranging from 360 to 440 nm when operating at a bias of 0.5 V, in comparison with the PD based on pure CsPbClBr_2_ NSs. The increased photocarrier density in the perovskite active layer of the Yb_3_^+^-doped device may be the cause of this appreciable rise in photocurrent values [328–330]. In Fig. 6h, the potential mechanism is shown. Yb^3+^ ions absorb near-infrared (NIR) light at approximately 980 nm, which excites the *4 f* electrons to a higher energy level (^2^F_7/2_). These electrons then undergo a two-step upconversion process to transition to the defect level at the bottom of the conduction band (as confirmed by DFT calculations). The photocurrent is eventually produced when they thermally transition to the conduction band of the CsPbClBr_2_ NSs. This process of direct photon electric upconversion is consistent with our previous observations [331]. The PD array-based visual photodetection system is a significant step towards the development of artificial "eye" devices.

Finally, there are difficulties in designing perovskite-based PDs, which scientists are working to resolve. Optimizing low-dimensional perovskite materials to improve PD performance is one of the main challenges [52]. Furthermore, to improve perovskite PDs’ functionality, researchers are investigating issues about potential future research directions and major roadblocks [64]. Furthermore, creating narrowband perovskite PDs involves difficulties that require creative solutions and a thorough comprehension of basic physics [332]. Moreover, creating novel materials with high responsivity and low detectivity levels is difficult in the pursuit of lead-free perovskite PDs [6]. These difficulties show how difficult it is to design perovskite-based PDs and draw attention to current initiatives to resolve them to advance this field.

## Perovskite-Based Nanorods

### Growth of Perovskite-Based Nanorod Arrays

The inherent instability of perovskite materials, stability problems, hysteresis in photocurrent density–voltage measurements, and toxicity concerns are challenges related to using perovskite NR arrays in photovoltaic devices. These difficulties hinder the large-scale use of perovskite solar cells and substantially impact their practical application. Several initiatives are being taken to address these problems, such as lowering toxicity, strengthening device fabrication methods to get around hysteresis and instability problems, and increasing the stability of perovskite materials in ambient environments [333, 334]. Stabilizing the materials, selecting the most effective encapsulation methods, and researching innovative perovskite architectures are crucial steps towards overcoming these challenges and advancing the actual application of perovskite NR arrays in photovoltaic devices. Perovskite NR arrays can be synthesized using innovative bottom-up techniques, enabling the accurate and scalable creation of nanocrystal arrays with control over size, number, and position. These arrays considerably facilitate the integration of perovskites into nanoscale optoelectronics. During the growing process, topographical templates enable localized growth and positioning with controlled surface wettability. Using this method, it is possible to create deterministic arrays of CsPbBr_3_ NCs with high positional accuracy and tunable dimensions as low as < 50 nm [335]. This approach opens up new possibilities for nanoscale PDs by providing flexible, scalable, and compatible procedures for perovskite integration into on-chip nanodevices [335]. Elements moving around, alterations in the chemical composition, heat and moisture in the environment, and crystal structural flaws are all causes of instability in perovskite NR arrays. Degradation can arise from elemental migration into the perovskite layer from layers such as ITO/FTO; subsequent chemical changes may affect stability. Perovskite materials may hydrolyse when exposed to moisture, which can cause irreversible degradation and disintegration [336]. Crystal structural flaws can speed up ion migration, cause phase separation or decomposition, and impact interfacial contact and carrier extraction [336, 337]. Additionally, local lattice strain, cage distortions, vacancy formation, and solar cell degradation can be caused by the mismatch of ionic sizes in perovskite materials [337]. Improving the stability of perovskite NR arrays in photovoltaic devices requires addressing these factors through defect passivation, encapsulation techniques, and compositional engineering.

**Fig. 7:**
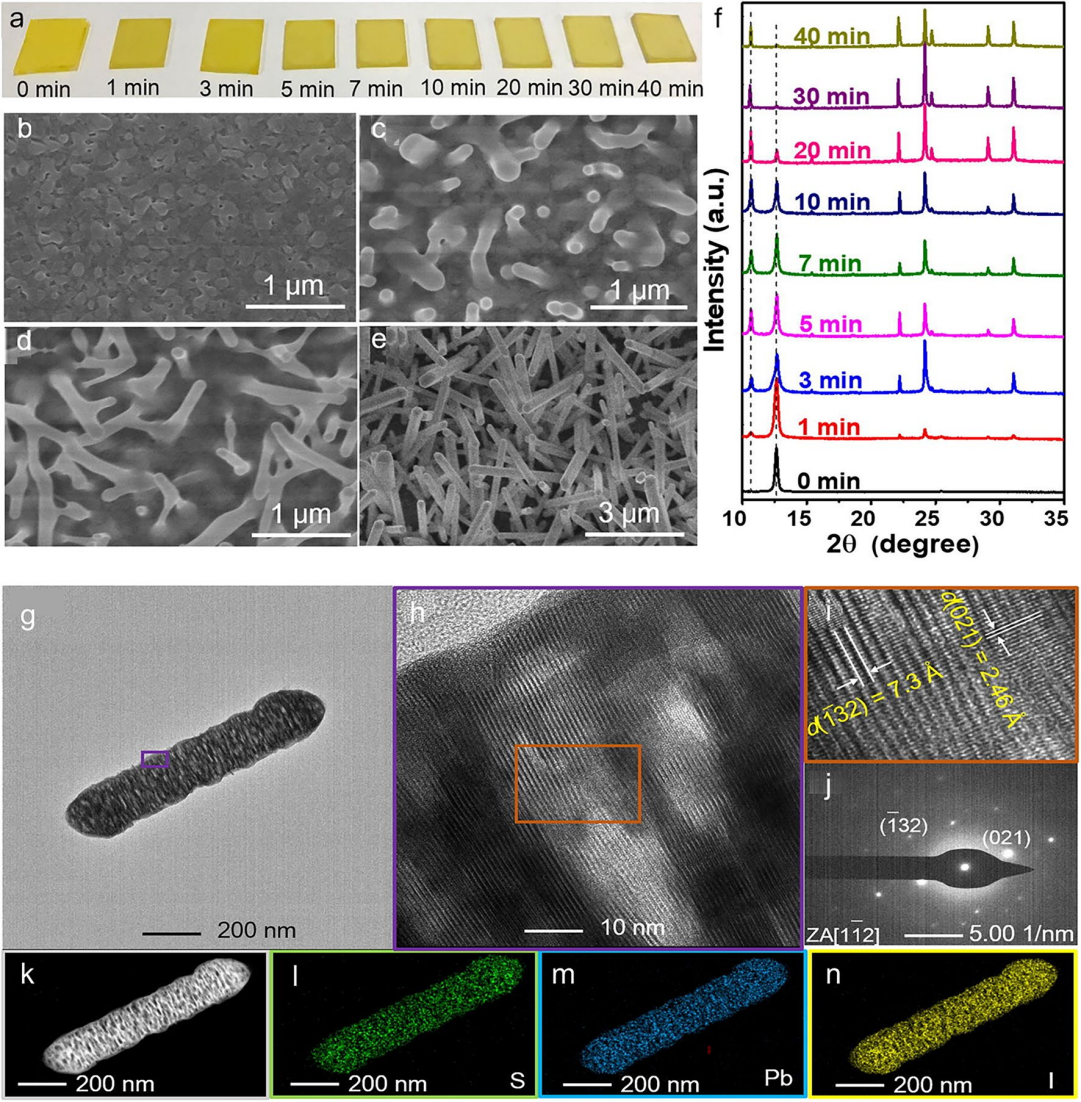
**a** PbI_2_ dipping in (CH_3_)_3_SI solution for varying times captured in optical images. PbI_2_ film dipping in (CH_3_)_3_SI solution for **b** 0 min, **c** 5 min, **d** 10 min, and **e** 30 min is shown in top-view SEM images. **f** PbI_2_ dipping in (CH_3_)_3_SI solution for varying times as seen in XRD patterns. **g**–**i** HRTEM and TEM pictures of a normal single (CH_3_)_3_SPbI_3_ NR. **j** Diffraction pattern of electrons along the axis of the [1–12] zone. **k–n** Elemental distribution of sulphur (S), lead (Pb), and iodine **i** in (CH_3_)_3_SPbI_3_ NR, measured by EDX. **a**–**n** Reproduce with permission [218]. Copyright 2021, Elsevier

Halide perovskite materials have shown notable progress in optoelectronic applications, especially in solar cells with amine components in organic cations. However, these amines’ N–H bonds make them vulnerable to hydrolysis when exposed to atmospheric moisture, jeopardizing the perovskite materials’ stability. Investigating substitute perovskite materials that do not require amines for stability is therefore necessary. Sulphur-based perovskite-like (CH_3_)_3_SPbI_3_ NR arrays were successfully created by Ruiyuan et al. [218] using a solution-processed method. The crystal structure of these arrays is hexagonal and can be indexed in the space group *P63mc*. The extraordinary stability of (CH_3_)_3_SPbI_3_ is due to the strong interaction between the non-amine (CH_3_)_3_S^+^ and [PbI_6_]^4−^ octahedral. These NR arrays remain morphologically and crystallographically intact in ambient conditions for more than 60 days. Moreover, they offer direct channels for charge transfer, displaying exceptional optoelectronic qualities. As shown in Fig. 7, SEM and XRD technologies analyse the dipping time of PbI_2_ films into (CH_3_)_3_SI ACN solution to investigate the reaction process between (CH_3_)_3_SI and PbI_2_. The optical images in Fig. 7a show how the PbI_2_ film’s bright yellow colour gradually changes to a paler shade after reacting with (CH_3_)_3_SI for various lengths. The SEM image (Fig. 7b) shows that the PbI_2_ film on the FTO substrate is uniformly dense and made up of particles about 100 nm in size. The morphology presented in Fig. 7c clearly illustrates the formation of (CH_3_)_3_SPbI_3_, which results from the reaction between (CH_3_)_3_SI and PbI_2_ after a 5-min immersion. As shown in Fig. 7d, the (CH_3_)_3_SPbI_3_ NRs are visible when the dipping time is increased to 10 min. The uniform (CH_3_)_3_SPbI_3_ NRs show well-crystallized structures with an average diameter of about 20 nm after extending the dipping time to 30 min (Fig. 7e). The (CH_3_)_3_SPbI_3_ NRs gradually expand during this process. These NRs have a hexagonal top shape similar to the c-axis direction growth pattern seen in ZnO NR arrays [338]. The XRD patterns of the samples dipped in the (CH_3_)_3_SI solution for different lengths of time are shown in Fig. 7f. Within the 2*θ* range of 10° to 35°, a single peak is seen at 12.6° (PDF: 80–1000, P3m1 (No.164)) in the PbI_2_ XRD pattern. This peak agrees with the PbI_2_ phase before the (CH_3_)_3_SI reaction [339]. As the dipping time increases, the distinctive PbI_2_ peak gradually becomes weaker and, after 30 min, vanishes, signifying the progression of the reaction between (CH_3_)_3_SI and PbI_2_. New peaks appear in the XRD patterns at 10.6°, 22.3°, 24.1°, 29.1°, and 31.1° after the reaction, indicating the formation of a novel phase. Moreover, there is evidence that the reaction can be finished in 30 min because the XRD patterns of the samples dipped for 30 and 40 min are almost the same. TEM was used to further investigate the crystal structure of (CH_3_)_3_SPbI_3_ NRs. A representative (CH_3_)_3_SPbI_3_ NR is shown in Fig. 7g. Lattice fringes are visible in the HRTEM image displayed in Fig. 7h and its magnified area in Fig. 7i. The interplanar spacings of 7.3 and 2.46 Å correspond to the (132) and (021) lattice planes, respectively. The (CH_3_)_3_SPbI_3_ NR’s selected area electron diffraction (SAED) pattern is shown in Fig. 7j along the [1–12] zone axis. The *d*-spacings for the (− 132) and (021) lattice planes are 3.6 and 2.46 Å, respectively. The EDX spectrum in Fig. 7k–n shows that S, Pb, and I are distributed uniformly throughout the NR and do not phase separately. The TEM and EDX data confirm the (CH_3_)_3_SPbI_3_ crystalline structure’s pure phase. Creating a brand-new stable array of perovskite NRs shows promising photoelectric properties and has potential uses in useful photoelectric fields.

### Perovskite Nanorod-Based Photodetectors

**Fig. 8:**
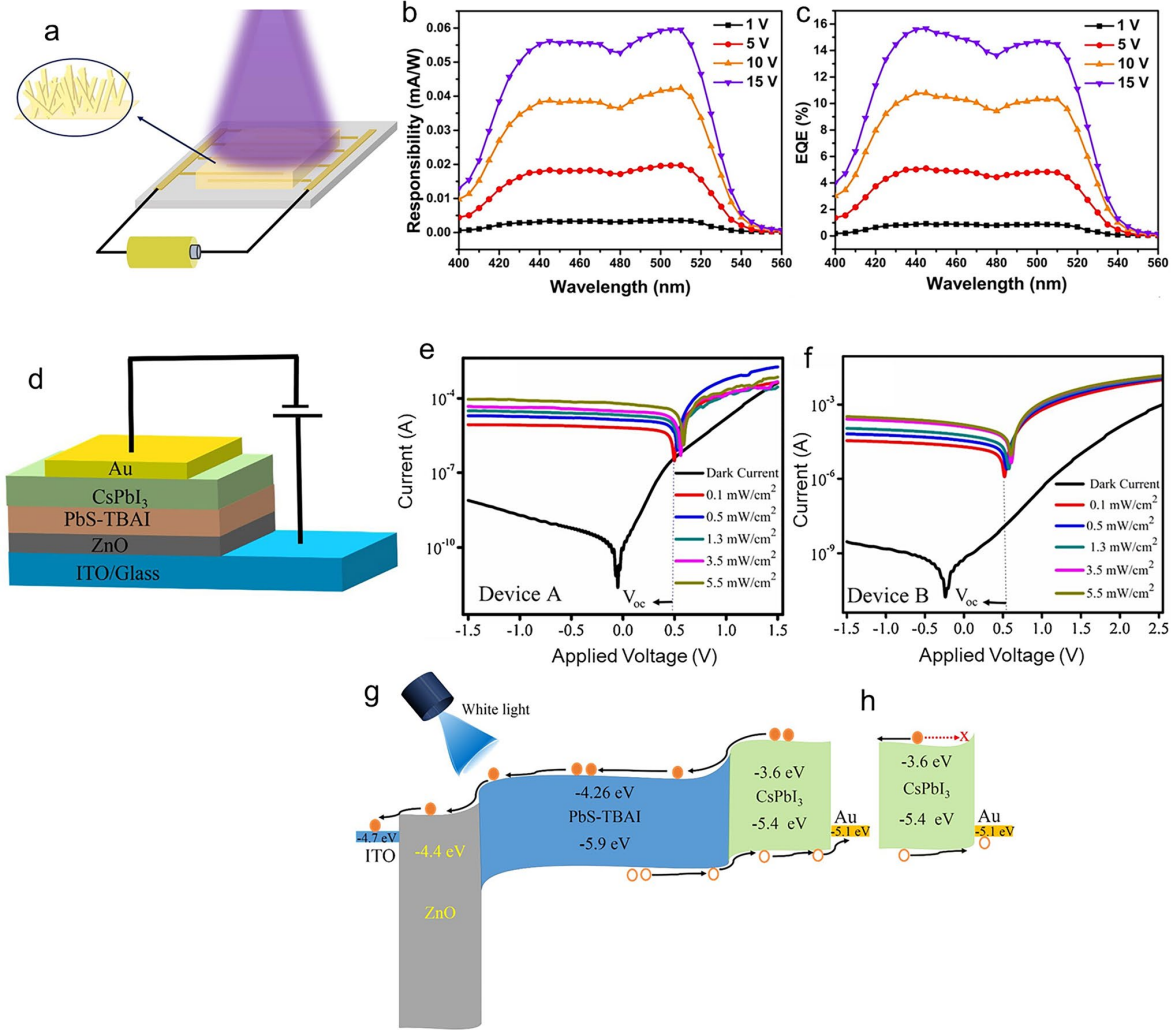
**a** Diagrammatic representation of the PD for (CH_3_)_3_SPbI_3_ NR arrays and its performance characteristics. **b** Response spectra. **c** EQE spectra of (CH_3_)_3_SPbI_3_ NR arrays PD under different forward bias scenarios. **a**–**c** Reproduce with permission [218]. Copyright 2021, Elsevier. **d** Cross-sectional representation. **e, f** *I*–*V* curves show the properties of devices A and B under different lighting conditions and in the dark, respectively. **g** A gradient energy band diagram showing the trilayer heterojunction of ZnO/PbS-TBAI/CsPbI_3_. **h** CsPbI_3_/Au interface forms an electron-blocking interface, which causes a band to tilt upward to improve electron-blocking and hole extraction. **d**–**h** Reproduce with permission [55]. Copyright 2021, Elsevier

Since perovskite NRs have remarkable photoelectric properties, they are being studied in great detail, especially for PD applications. These NRs are used in phototransistors, photodiodes, and photoconductors, among other perovskite PDs. Using perovskite NRs with organic semiconductors has demonstrated encouraging results, particularly in creating high-efficiency phototransistors that exhibit exceptional stability and photosensitivity [64, 221]. Furthermore, compared to other perovskite-based PDs, hybrid devices combining perovskite NRs and organic semiconductors have demonstrated improved photosensitivity, responsivity, and stability [221]. Furthermore, developing high-performance broadband PDs has been aided by incorporating triple-cation perovskite/ZnO NRs [340]. The long carrier lifetime, high carrier mobility, and quick response times of nanostructured perovskites—which come in 2D, 1D, and 0D structures—make them highly promising for photodetection applications [51]. Sulphur-based perovskite-like (CH_3_)_3_SPbI_3_ NR arrays were fabricated using a solution-processed method [218]. When assessing (CH_3_)_3_SPbI_3_ NR array PDs, responsiveness (R), measured under different bias conditions and compared to Si standard PD, is a crucial factor. A schematic diagram of the (CH_3_)_3_SPbI_3_ NR arrays PD is shown in Fig. 8a. The findings are shown in Fig. 8b, which shows that the responsivity rises as the voltage does. Furthermore, the spectral photoresponse exhibits broadband detection, with a peak platform detected at 15 V between 430 and 520 nm. 0.06 mA W^−1^ is the maximum responsivity value below 15 V. EQE is carried out at different biases, as Fig. 8c illustrates [341]. Through the external circuit, charges can be injected from the electrodes under applied voltage. Similar to responsivity, EQE shows a trend of increasing with increasing voltage at different voltages. Under a 15 V bias, the highest EQE value reaches 16%. The photovoltaic materials can produce electron–hole pairs when exposed to light. The electron–hole pairs are then quickly separated and gathered by electrodes in the presence of an applied electric field [342]. Promising photoelectric properties have been demonstrated by a newly developed stable array of NRs. There may be useful uses for this development in the field of photoelectric technology.

Achieving heterojunction formation is thought to be essential for producing high-performance devices. In particular, self-powered PDs benefit from forming a gradient energy band between heterojunctions. The synthesis of CsPbI_3_ NRs and their use as the interfacial layer in high-performance, all-solution-processed self-powered PDs were presented by Saleem et al. [55]. Following the successful growth of PbS QDs and CsPbI_3_ NRs (NRs), two different kinds of PDs were created. Device A represented the first type: ITO/ZnO (100 nm), PbS-TBAI (150 nm), and Au. Device B represented the second type, which contained ITO/ZnO (100 nm), PbS-TBAI (150 nm), CsPbI_3_ (250 nm), and Au. Figure 8d displays device B’s cross-sectional diagram. Figure 8e, f shows devices A and B’s current vs. voltage (*I–V*) curves. The *I–V* curves’ generally rectifying behaviour shows that heterojunctions have formed in devices A and B. It is evident that, compared to device A (∼ 10^–8^ A), device B displays a lower dark current (∼ 2.94 × 10^−9^ A). Furthermore, under 0.1 mW cm^−2^ white light illumination, device B’s obtained On/Off current ratio of 10^4^ is higher than that of device A (i.e. 10^3^). The band gap offset between the CsPbI_3_ NRs layer and the PbS-TBAI NRs layer explains device B’s reduced dark current. This makes sense in the following ways: under illumination, device A exhibits a comparatively higher likelihood of carrier recombination for photogenerated carriers. On the other hand, device B’s heterojunction between PbS-TBAI and CsPbI_3_ makes it easier for photogenerated excitons to separate. Furthermore, a greater photocurrent is produced due to the significant reduction in interfacial recombination caused by the spatial dissociation of photogenerated electrons and holes at the interface. Remarkably, the strong Lewis acid–base interaction is the main cause of device B’s low hysteresis nature compared to device A. With the assistance of two layers and the interfacial layer, this interaction makes it possible to establish electron pair donation effectively. As a result, surface traps are passivated, and charge carrier recombination is prevented [343]. In order to understand the heterostructure design and device B’s improved self-powered mode performance, Fig. 8g, h schematically illustrate the possible energy band alignments between PbS-TBAI and CsPbI_3_ NRs. The conduction band level (*E*_c_) and valence band level (*E*_v_) of PbS-TBAI, ZnO nanoparticles, and CsPbI_3_ NRs film were measured. They are − 4.26 and − 5.9 eV, − 4.4 and − 7.7 eV for ZnO, and − 3.6 and − 5.4 eV for CsPbI_3_ NRs. ITO and Au have work functions of − 4.7 and − 5.1 eV, respectively. When exposed to white light, the PbS-TBAI and CsPbI_3_ layers are the primary sources of charge carriers because the incident photon energy (3.1–1.77 eV) is lower than the band gap of ZnO nanoparticles (3.34 eV). At the CsPbI_3_/Au interface, electrons are blocked due to the Au electrode’s work function of − 5.1 eV. As a result, as shown in Fig. 8h, the energy band at this interface bends sharply upward, producing a depletion region. As a result, under the applied electric field, holes created in CsPbI_3_ transfer to Au, electrons transfer to PbS-TBAI, then to ZnO and the ITO electrode, respectively. These findings confirm that interfacial recombination can be successfully decreased, and device performance improved by appropriately inserting an interfacial layer in heterojunctions. As a result, it offers a solid basis for optoelectronic material and device configuration. As a subset of perovskite-based PDs with various device types and applications, perovskite NR-based PDs have attracted much attention. The research goal until now has been to use nanostructured perovskites to increase performance. Photovoltaic and photoconductive devices with their spatial configurations are included in perovskite-based PDs [51]. Advancements in recent times have highlighted multifunctional capabilities like angle-sensing, spectral, and polarization light detection [35]. Developments highlight the adaptable morphologies, compositions, and structures of perovskite materials, highlighting their potential for various uses [18]. These results highlight continued efforts to improve perovskite-based photodetection technology’s functionality, performance, and adaptability.

## Perovskite-Based Nanowires

### Growth of Perovskite-Based Nanowires

The diverse development methods of perovskite NWs make them a promising example of controlled growth techniques. Methods include printing perovskite NWs with inkjet technology and nanoporous anodic alumina templates [344], synthesizing perovskite NWs by solution processes like hot injection, self-assembly, solvothermal, and anion exchange [58], and directing growth induced by graphoepitaxial effects on annealed M-plane substrates [345]. Additionally, polar solvent-directed growth has been studied quickly, and this approach works well to increase the variety of morphologies that can be achieved in perovskite NWs [346]. Because of the precise alignment and patterning capabilities made possible by techniques like inkjet printing and nanopore-confined growth, these advancements in growth techniques have potential applications in fields such as lighting and lasing [58]. One of the current obstacles to the controlled growth of perovskite NWs for lighting technology is the creation of a 2D surface appropriate for solar cell applications. Further obstacles include patterning, aligning, and transferring perovskite NWs for lighting technology applications [58]. It is not easy to induce directional growth for particular morphologies, such as CsPbBr_3_ NWs, affecting how well they integrate into lighting technologies [56]. These drawbacks show that in order to integrate perovskite NWs into lighting technology successfully, specific growth techniques must be addressed.

**Fig. 9:**
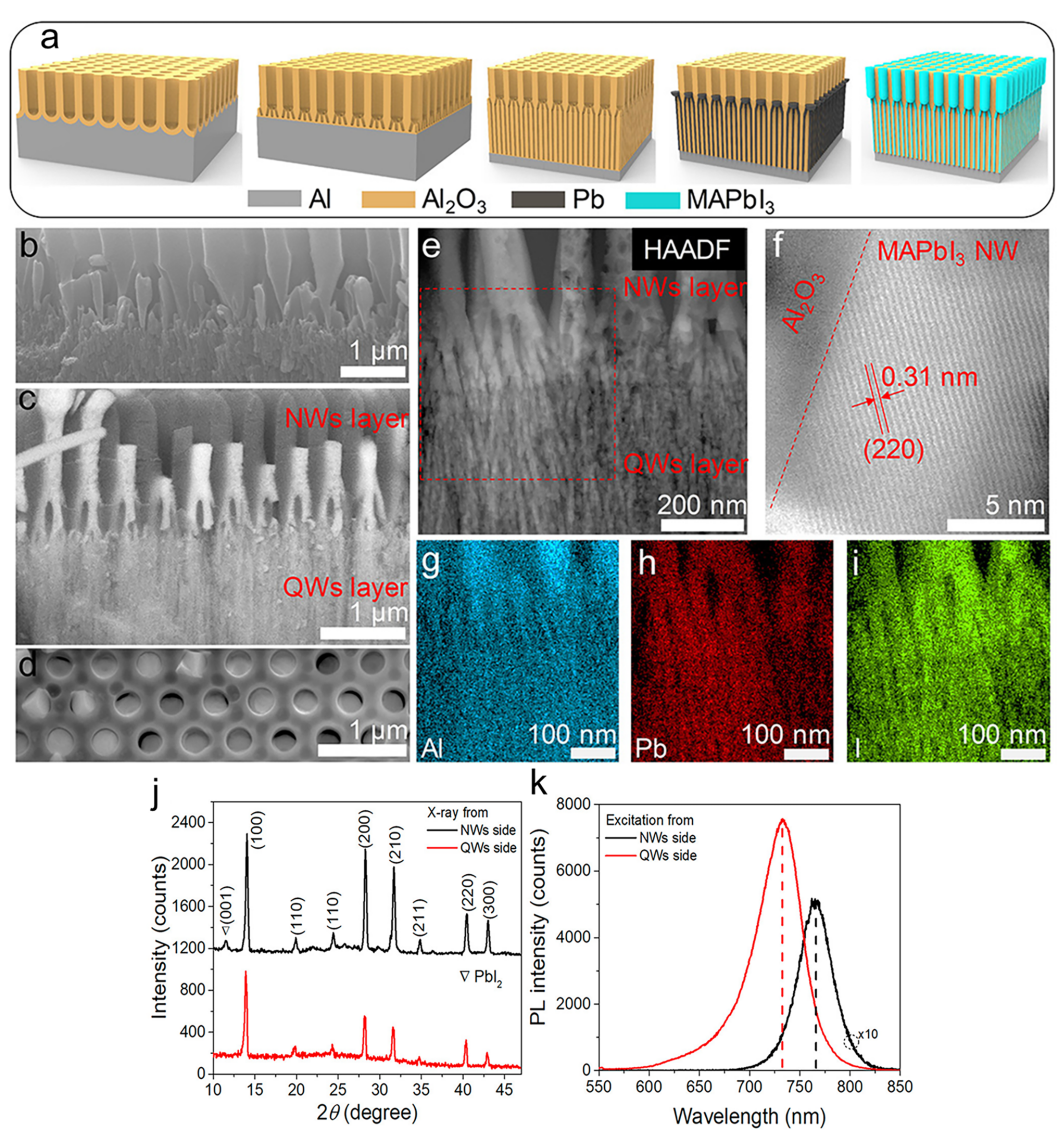
Following steps are involved in the fabrication process from left to right: 200 V anodization, barrier thinning, 5 V anodization, Pb electrochemical deposition, and VRS growth. The images displayed are cross-sectional SEM images of a PAM **b** with and **c** without MAPbI_3_ QWs/NWs growth. **d** A top-view SEM image shows the BSE measurement mode where MAPbI_3_ QWs/NWs are embedded in the perfectly ordered PAM. The MAPbI_3_ QW/NW junction is shown in HAADF-STEM images at **e** low and **f** high magnifications. **g**–**i** MAPbI_3_ QW/NW junction selective area EDX mapping; the area is indicated by the red dashed line in the image (**e**). **j** X-ray is shown separately, shining from the NW and QW sides in the XRD patterns. **k** PL spectra with excitation light (λex = 350 nm) shining separately from the NW and QW sides. **a**–**k** Reproduce with permission [231]. Copyright 2022, ACS

Metal halide perovskite (MHP) single crystals are an efficient means of achieving optical filter-free narrowband photodetection through charge collection narrowing (CCN). Nevertheless, the need for thick crystals in CCN limits their applicability to large-scale, flexible, self-driven, and high-performance optoelectronic applications. Vertically integrated MHP quantum wire/NW (QW/NW) array-based PDs within nanoengineered porous alumina membranes (PAMs) have been reported by Daquan et al. [231]. This invention exhibits simultaneous self-driven narrowband photodetection and broadband photodetection capabilities. A schematic representation of the QW/NW array’s growth process is shown in Fig. 9a. It consists of two main steps: the creation of dual-diameter PAMs and the growth of MAPbI_3_ assisted by vapour–solid reaction (VSSR). Because the PAM pore size is directly proportional to the anodization voltage, the dual-diameter PAM is produced by electrochemically anodizing aluminium (Al) foil in two steps at different voltages. A high voltage (200 V) anodization is first applied to create large-pore PAM. The corresponding SEM image (Fig. 9b) shows that the average pore size of the PAM is approximately 200 nm. The barrier layer (Al_2_O_3_) is then sufficiently thinned by another barrier thinning procedure to prepare it for the following low-voltage anodization. The brightness contrast in the cross-sectional back-scattered electron (BSE) SEM image (Fig. 9c), where the light area represents MAPbI_3_. and the dark area corresponds to anodic alumina, makes the QW/NW arrays easily distinguishable. SEM images captured from above of MAPbI_3_ NWs in perfectly ordered and semi-ordered PAMs are shown in Fig. 9d. It is important to note that the anodization and nanoimprint methods are used to fabricate the perfectly ordered PAM. Such fabrication is advantageous for accurately addressing individual NWs in ultrahigh-resolution imaging applications. A high-angle angular dark field–scanning transmission electron microscopy (HAADF-STEM) image of the QW/NW transition area, showing the ultrahigh-density quantum wires (QWs) clearly, is shown in Fig. 9e at low magnification. At the same time, the boundary between MAPbI_3_ and Al_2_O_3_ NWs is visible in the high-magnification image shown in Fig. 9f, which also exhibits excellent surface passivation and crystallinity. The high-temperature VSSR growth within the Al_2_O_3_ template is primarily responsible for this extraordinary quality [347, 348]. Energy-dispersive X-ray spectroscopy (EDX) mapping of the indicated region in Fig. 1e is shown in Fig. 9g–i. Excellent material quality is indicated by the uniform distribution of elements in both quantum wires (QWs) and NWs. The XRD patterns of the MAPbI_3_ quantum wire/NW (QW/NW) arrays, with light emanating from the NWs and QWs independently, are displayed in Fig. 9j. There is only one discernible set of crystalline peaks in the patterns, suggesting that the cubic crystal structure is identical for both NWs and QWs. The PbI_2_ (001) phase is responsible for a minor XRD peak at about 11.5° for the NWs, as shown in Fig. 9j. Since the perovskite decomposition process usually starts from the top surface of the QWs, this peak is not visible. The PL spectra of QWs and NWs under each side-illuminated excitation are shown in Fig. 9k. Two PL peaks are detected at 731 and 766 nm, with 1.70 and 1.61 eV optical band gap values for MAPbI_3_ QWs and NWs, respectively. The effects of quantum confinement and surface passivation, which are in charge of the band gap increase and the noticeable improvement in the PL signal for QWs have already been covered in detail [347].

Perovskite NWs’ special qualities and possible uses have attracted much research interest. Perovskite NWs have grown and are being used due to several studies. Controlled growth of in-plane directional perovskite CsPbBr_3_ NWs was demonstrated using a graphoepitaxial effect [344]. Researchers used inkjet printing and nanoporous anodic techniques to demonstrate printed perovskite NWs for lasing and phosphor applications [58]. The methods and procedures used to create halide perovskite NWs include self-assembly, vapour-phase growth, and hot injection [345]. The synthesis of long ferroelectric NWs with perovskite structures using different solution techniques [4]. Researchers presented a straightforward low-temperature growth technique to demonstrate the potential applications of vertically aligned CsPbBr_3_ NW arrays [349]. Together, these studies expand our knowledge of perovskite NWs and their potential uses in various technological fields, including optoelectronics, lasing, and PDs.

### Perovskite Nanowire-Based Photodetectors

The development of high-performance PDs appears to be greatly promising for perovskite NWs. The research has focused on using single-crystalline, solution-processable perovskite NWs to create inexpensive PDs with high detectivity [237]. Furthermore, advancements in PDs’ sensitivity have been made by employing conductive materials, self-assembled quantum wells, and strongly interacting layered metal–halide perovskites [234]. Additionally, lead-free perovskite alloy NW PDs have demonstrated outstanding performance, providing opportunities for improved devices in this field [350]. Furthermore, creating long-lasting, flexible perovskite NW PDs highlights their potential as next-generation photodetection technologies [230]. Various options are among the most promising materials for perovskite NW PDs. The capacity to integrate the benefits of both organic and inorganic components leads to enhanced performance and versatility in photodetection applications, which makes organic–inorganic hybrid perovskites unique [223, 237]. Furthermore, solution-processable single-crystalline perovskite NWs present a strong chance to create highly detective, reasonably priced PDs, making them leading candidates for next-generation photodetection technologies [33]. Lead-free perovskite alloy NWs, especially those with a one-dimensional structure, are another noteworthy contender. These single-crystal NWs exhibit good performance in PDs, which makes them highly desirable for advanced photodetection applications [350, 351].

**Fig. 10:**
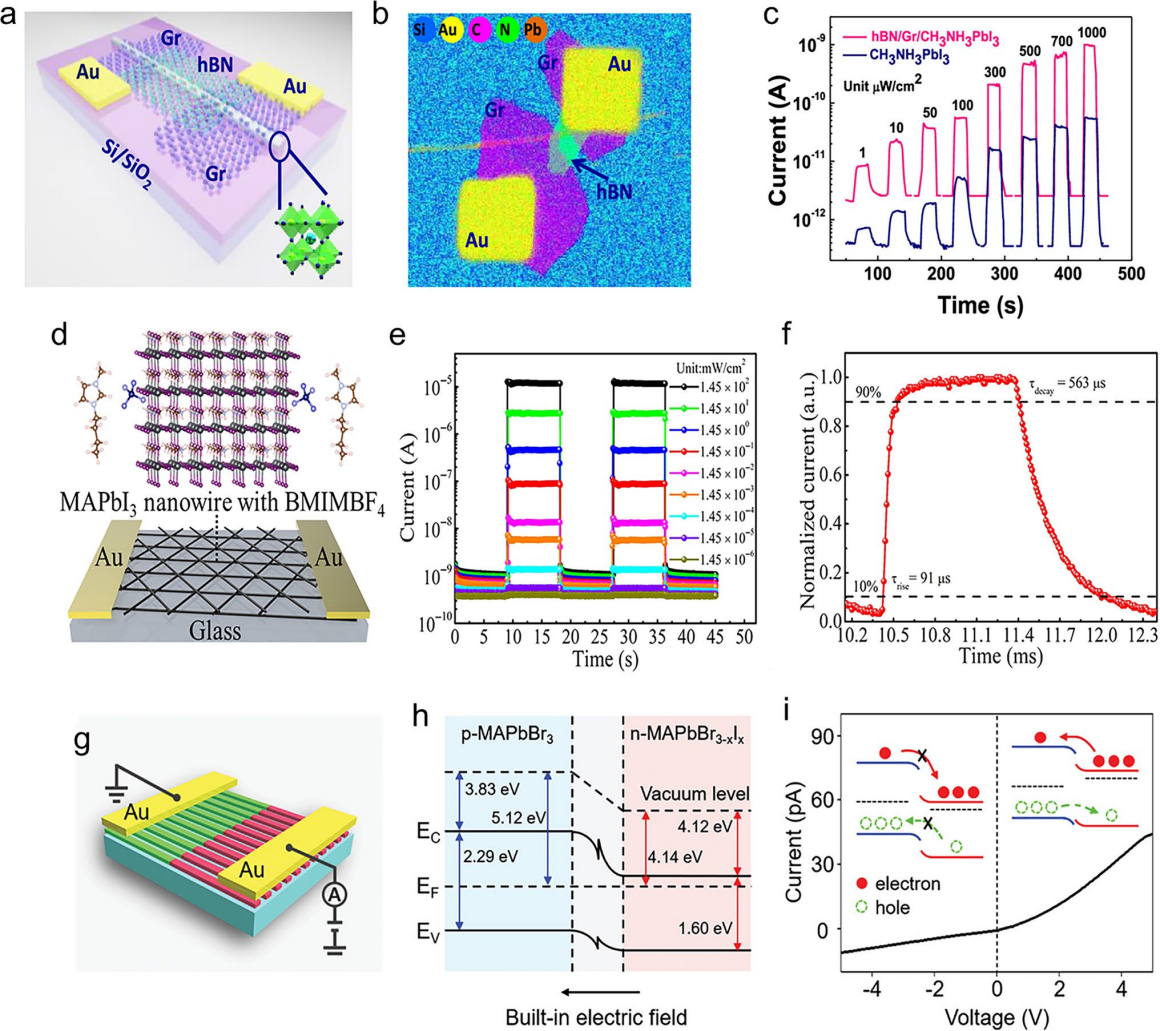
**a** Device’s schematic diagram. **b** An EDS image. **c** Photoswitching characteristics of both the CH_3_NH_3_PbI_3_ device and the h-BN/Gr/CH_3_NH_3_PbI_3_ device were assessed at 2 V under a 655 nm unfocused laser. The light intensity ranged from 1 to 1000 mW cm^−2^. **a**–**c** Reproduce with permission [233]. Copyright 2022, ACS. **d** Au/MAPbI_3_ NW/Au structure device with BMIMBF_4_ (0.6 mmol) is shown in the schematic diagram. At a bias of 5 V, the Au/MAPbI_3_ NW/Au structure device was tested for performance using BMIMBF_4_ (0.6 mmol). **e** Different light intensities were used to observe the *I*–*t* curves. **f** Response time was recorded at 14.5 mW cm^−2^ of light intensity. **d–f** Reproduce with permission [230]. Copyright 2022, Wiley. **g** MAPbBr_3_/MAPbBr_3−*x*_I_*x*_ p–n–junction device is depicted schematically in the diagram. **h** MAPbBr_3_/MAPbBr_3−*x*_I_*x*_ p–n junction’s energy band alignment under thermal equilibrium. According to the band structures, MAPbBr_3_ is a p-type conductor, and MAPbBr_3−*x*_I_*x*_ is an n-type semiconductor. At the p–n junction’s interface, an inherent electric field points from the *n*-type MAPbBr_3−*x*_I_*x*_ to the *p*-type MAPbBr_3_. **i** MAPbBr_3_/MAPbBr_3−*x*_I_*x*_ p–n–junction device’s *I*–*V* curve at a 5 V bias is shown in the dark. The device exhibits a clear current rectifying behaviour, confirming the creation of a p–n junction. **g**–**i** Reproduce with permission [229]. Copyright 2022, Wiley

The organic–inorganic hybrid perovskite NW, CH_3_NH_3_PbI_3_, becomes a promising candidate for high-performance PDs due to its remarkable photoresponse. However, difficulties remain concerning ineffective carrier collection between the metallic electrodes and one-dimensional (1D) NWs. Furthermore, the perovskite’s degradation makes CH_3_NH_3_PbI_3_ NWs less viable for commercial production. These obstacles highlight the necessity for creative approaches to maximize carrier collection efficiency and improve perovskite stability in order to fully realize the potential of CH_3_NH_3_PbI_3_ NWs in useful photodetection applications. Guanghui et al. [233] presented a PD with a hybrid van der Waals (vdW) heterostructure made of graphene (Gr)/1D CH_3_NH_3_PbI_3_ and hexagonal boron nitride (h-BN). Gr and the NW’s electrical contact facilitated enhanced carrier extraction, which allowed for remarkable responsivity and specific detectivity, reaching 558 A W^−1^ and 2.3 × 10^12^ Jones, respectively, in this configuration. The EDS and schematic images of the h-BN/Gr/CH_3_NH_3_PbI_3_ mixed-dimensional vdW heterostructure device are shown in Fig. 10a, b. The photoswitching properties of the h-BN/Gr/1D CH_3_NH_3_PbI_3_ mixed-dimensional vdW heterostructure device (h-BN/Gr/CH_3_NH_3_PbI_3_ device) and the CH_3_NH_3_PbI_3_ NW device (CH_3_NH_3_PbI_3_ device) are shown in Fig. 10c for a bias voltage of 2 V, covering a light intensity range of 1–1000 μW cm^−2^. As the light intensity increases, the photocurrent also increases, which is consistent with the theory that the incident photon flux and the number of photogenerated carriers are equal. Interestingly, the h-BN/Gr/CH_3_NH_3_PbI_3_ device shows a significantly amplified photocurrent with an on/off ratio of up to 10^3^, significantly higher than that of the CH_3_NH_3_PbI_3_ device. The h-BN/Gr/CH_3_NH_3_PbI_3_ device can remarkably show detectable photocurrent even in extremely low illumination (1 μW cm^−2^). The mixed-dimensional vdW heterostructure h-BN/Gr/1D CH_3_NH_3_PbI_3_ provides a novel concept and manufacturing process for high-performance, air-stable photoelectronic devices using organic–inorganic hybrid perovskite NWs.

Perovskite NW-based PDs are among the most promising next-generation photodetection technologies. However, their lack of long-term stability is a major obstacle to their commercial viability. Dingjun et al. [230] combined methylammonium lead triiodide (MAPbI_3_) NWs with 1-butyl-3-methylimidazolium tetrafluoroborate (BMIMBF_4_), an ionic liquid. This method effectively creates nanochannels to speed up charge transfer while also passivating defects to prevent perovskite degradation. A specific concentration of BMIMBF_4_ (0.6 mmol) was used to improve device performance. Then, on a glass substrate, an Au/MAPbI_3_ NW/Au structure device was created (Fig. 10d), and its functionality was carefully investigated. Then, under various light intensities, this device’s *I*–*t* curves were recorded (Fig. 10e). It exhibits a consistent and noteworthy transient current response across all light levels, with a particularly robust response at 1.45 × 10^−6^ mW cm^−2^, a low light level, underscoring its remarkable photoresponse capability. The device is suitable for real-world applications and has rise (*τ*_rise_) and decay (*τ*_decay_) times of 91 and 563 μs, respectively, as shown in Fig. 10f. This method provides a fresh approach to creating flexible, stable, and sensitive perovskite PDs, which could hasten their eventual commercial adoption.

Today’s optoelectronic integrated circuits require highly sensitive PDs as essential parts. Because of their effective carrier separation, p–n junction construction has become a potent method for obtaining sensitive photodetection. Lately, there has been much interest in the practical applications of p–n junction PDs based on organic–inorganic hybrid perovskites. These PDs offer easy processability and favourable optoelectronic performance. Unfortunately, most currently used devices are made of polycrystalline films, which have a low carrier transport efficiency and cannot be made to enhance their photoresponsivities. A particular kind of ultrasensitive PD based on single-crystalline perovskite p–n junction NW arrays has been demonstrated by Guan et al. [229]. A lateral configuration device was built to investigate the optoelectronic and electronic properties of the MAPbBr_3_/MAPbBr_3−*x*_I_*x*_ p–n junction arrays (Fig. 10g). In lateral devices, the heterojunction’s small contact area can reduce the abundance of defects at the interface, while direct light interaction with the active layer can significantly minimize light reflection and loss [352]. The Fermi level of MAPbBr_3_ is near the top of the valence band, as shown in Fig. 10h, which is similar to the properties of a p-type semiconductor [353]. The calculations show that the Fermi energy (EF) and valence band maximum energy (EVBM) of MAPbBr_3−*x*_I_*x*_ are 4.14 and 5.72 eV, respectively. MAPbBr_3−*x*_I_*x*_ exhibits a prominent n-type characteristic, as evidenced by its Fermi level near the bottom of the conduction band [354]. A built-in electric field directs from the n-type MAPbBr_3−*x*_I_*x*_ to the p-type MAPbBr_3_ at the p–n junction interface when the Fermi levels of MAPbBr_3_ and MAPbBr_3−*x*_I_*x*_ are aligned under thermal equilibrium. Effective carrier separation depends on this inherent electric field. Further investigation into the p–n junction device’s rectifying performance revealed typical p–n diode characteristics. The current–voltage (*I*−*V*) curve of the p–n junction device is shown in Fig. 10i under dark conditions, clearly demonstrating current rectifying behaviour. In conclusion, the carrier transport barrier is overcome under forward bias (positive potential on MAPbBr_3_), while the barrier is further enlarged under reverse bias, leading to electrical rectifying characteristics. The perovskite p–n junction’s rectifying ratio is calculated to be roughly 4, which is similar to perovskite heterojunctions that have been previously reported [355]. Effective carrier separation is facilitated by the p–n junction’s inherent electric field, which is experimentally demonstrated by the observed current rectifying behaviour. These findings open up new avenues for designing and constructing high-performance PDs, which will greatly expand the uses of these devices in optoelectronic integrated circuits.

Perovskite NWs have drawbacks that prevent further improvements in sensitivity and performance in PDs despite their high detectivity [237]. For perovskite NW-based PDs to function as well as possible, careful control over the growth and assembly of the NWs is required during the fabrication process [230]. Environmental influences and material deterioration pose a long-term stability challenge for flexible perovskite NW PDs, potentially compromising device reliability. Achieving high-performance PDs is challenging because controlling defect density in perovskite NWs enhances charge carrier mobility and overall device efficiency [230]. The material composition and structure of lead-free perovskite alloy NWs present challenges that call for creative solutions to maximize their functionality and performance in photodetection applications [332]. The performance of lead-free perovskite alloy NWs is being optimized through innovative material compositions and structural designs. These initiatives aim to address issues related to material functionality and stability in photodetection applications [350].

## Perovskite-Based Quantum Dots

### Growth of Perovskite-Based Quantum Dots

Perovskite-based QDs present several challenges in developing PDs, which scientists are working to address. A notable challenge is realizing peak performance in QD-based PDs even though they can absorb light and release excited electrons or fluorescence [356]. QD synthesis is a challenging process that frequently calls for high temperatures and inert atmospheres, which makes mass production and scalability for PD applications difficult [357]. Furthermore, the stability and reliability of QD-based PDs must be guaranteed for their practical implementation, which calls for continuous performance under changing circumstances over an extended period [357]. Researchers are committed to improving QD-based PDs’ scalability, performance, and dependability for various uses in light detection and other fields. The most effective methods for cultivating QDs for use in PDs include a variety of approaches. First, enhanced PD construction incorporates QDs and atomic structures into device architectures to improve overall performance [356]. Second, scalability and mass production of QDs depends on optimizing the synthesis process, which calls for research into growth conditions that maximize yield and efficiency for PD applications [357]. Thirdly, the efficiency and reliability of CsPbBr_3_-based PDs may be increased by surface passivation methods like congeneric QDs, potentially improving performance and stability [357]. Last, using superior growth techniques to create CsPbBr_3_ single-crystal PDs shows promise for improving dependability and performance. In order to address QD growth obstacles for PDs, researchers are focusing on these strategies with the ultimate goal of improving light detection applications’ scalability, performance, and dependability [357].

**Fig. 11:**
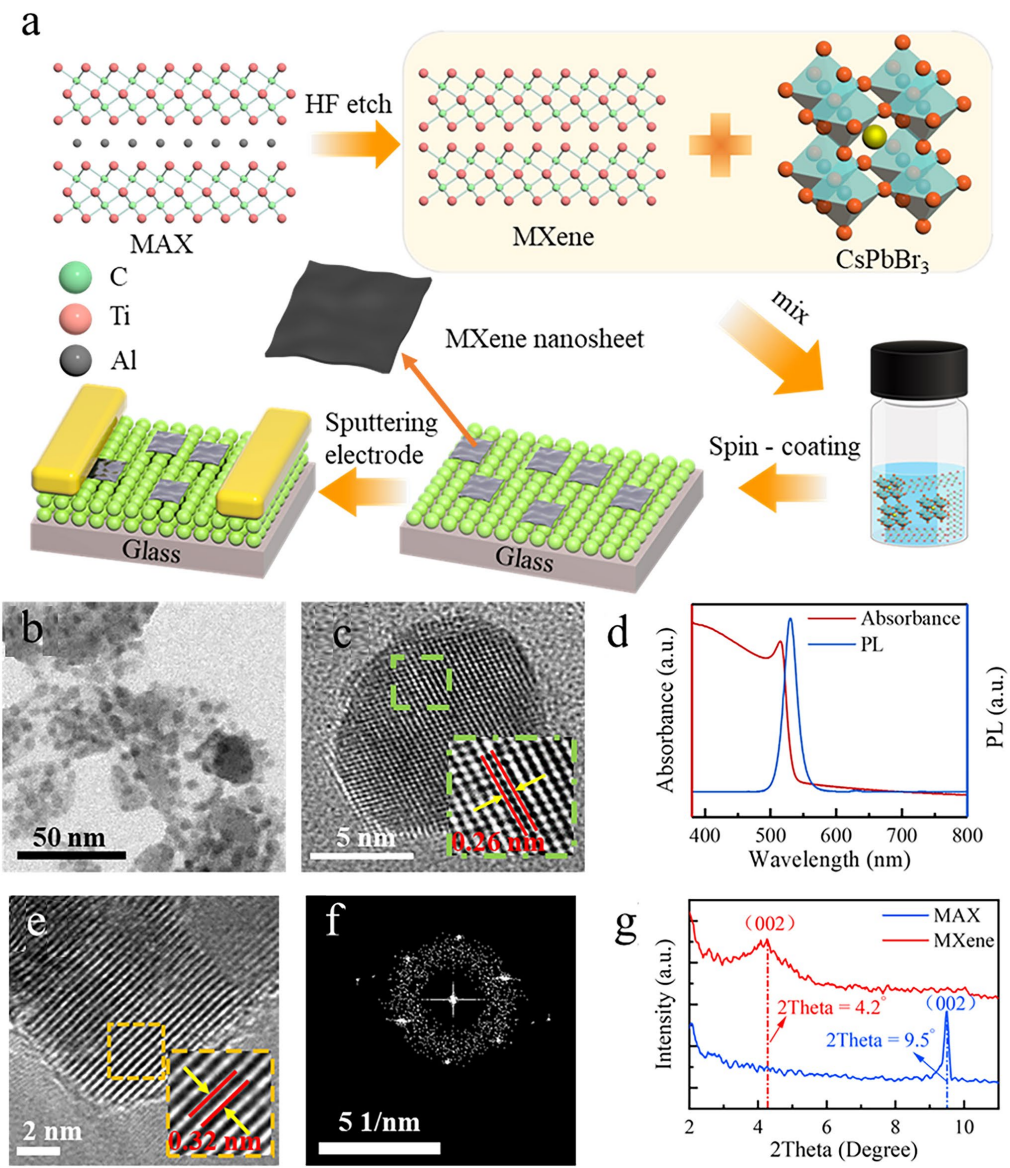
**a** Schematic construction of CsPbBr_3_ QD/MXene NS PDs. **b** A TEM image, and **c** an image of CsPbBr_3_ QDs obtained using an HRTEM. **d** Room-temperature absorbance spectrum (red) and PL emission spectrum (blue) of CsPbBr_3_ QD solution. The resulting MXene NSs’ **e** HRTEM pictures and **f** selected area electron diffraction (SAED) pattern. **g** Red and blue XRD spectra of MXene and MAX **a-g**. Reproduce with permission [258]. Copyright 2022, Elsevier

The exceptional optoelectronic characteristics and increased stability of all-inorganic CsPbBr_3_ QDs have made them a novel photoelectric material suitable for photodetection applications. Nevertheless, creating high-performance PDs is severely hampered by multiple trap states and ineffective carrier transport. Hao et al. [258] methodically showed how to blend 2D Ti_3_C_2_T_X_ NSs uniformly in order to increase the performance of CsPbBr_3_ QD PDs significantly. The process of creating CsPbBr_3_ QD/MXene NS PDs is illustrated in Fig. 11a by mixing different MXene concentrations with CsPbBr_3_ QD solution. The resulting spherical CsPbBr_3_ QDs, which have an average size of about 9 nm, show uniformity in Fig. 11b. The HRTEM image in Fig. 11c shows the interplanar spacings of 0.263 nm, which correspond to the (210) plane of the cubic crystal phase CsPbBr_3_ [358]. The absorption edge appears sharply below about 514 nm, as shown in Fig. 11d and a narrow PL emission peak is seen at about 530 nm. This observation suggests that there should be a radical recombination at the conduction band edge [359]. The exposure of the hexagonal structure’s (008) plane is indicated in Fig. 11e [360]. The SAED pattern in Fig. 11f shows that the 2D Ti_3_C_2_T_X_ NSs’ inherent hexagonal lattice structure did not change during synthesis. Three distinct peaks can be seen in the absorption spectra of the 2D Ti_3_C_2_T_X_ NSs at roughly 260, 325, and 780 nm. These peaks are ascribed to plasmonic absorption in the near-infrared (NIR) and the interband transition of MXene in the ultraviolet (UV) regions [361]. The corresponding XRD patterns are shown in Fig. 11g, which covers a small range between 2° and 8°. As shown, the 2D Ti_3_C_2_T_X_ NSs exhibit a slightly different (002) peak at 4.2° from the Ti_3_C_2_T_X_ powders. The significant increase in the interlayer distance is what causes this shift [362]. The optimized performance was consistently observed over a period of 4 months, even under atmospheric conditions, suggesting a viable strategy to address the challenges in fabricating perovskite optoelectronic devices for industrial applications.

The growth of perovskite-based QDs for PDs presents several challenges that researchers are actively addressing to achieve high performance, scalability, and stability [356]. In this endeavour, several strategies are being pursued. First, efforts are being made to improve overall device performance and PD construction by integrating QDs and atomic structures. Additionally, synthesis process optimization is receiving top priority in order to improve QD production’s scalability and suitability for mass manufacturing. Investigating growth environments that can boost QD yield and efficiency for PD applications [363]. Congeneric QD surface passivation approaches are also being researched to improve the stability and performance of CsPbBr_3_-based PDs, hence raising their dependability and efficiency. Additionally, researchers are investigating high-quality growth techniques to create CsPbBr_3_ single-crystal PDs, which have shown promise in achieving high performance and reliability [363]. These strategies aim to improve performance, scalability, and reliability in light detection applications by helping researchers overcome the difficulties involved in growing QDs for PDs [357].

### Perovskite Quantum-Dot-Based Photodetectors

Perovskite-based QDs have become essential elements in developing advanced PDs with distinct features. These PDs demonstrate broad-spectrum photodetection capabilities covering UV to visible light wavelengths. QD integration with materials such as MoS_2_ has resulted in robust photocurrents and efficient PDs operating at various wavelengths [255]. Research findings demonstrate the adaptability of QDs in a range of PD architectures, including field-effect transistors, photodiodes, and PDs. QDs, especially those based on caesium bromide and iodide, are suitable for visible light detection due to their remarkable light absorption and emission characteristics. Other types of QDs, however, are designed for ultraviolet or infrared light detection [364]. Moreover, recent developments have integrated QDs with vertically aligned graphene arrays to create ambipolar multifunctional PDs. With improved light absorption, electron transport, and photoinduced electron–hole pair separation, this integration produces remarkable photocurrent responses with higher detectivity and responsivity at particular wavelengths [4]. A new era of high-performance devices with tailored light absorption characteristics across multiple spectral ranges and efficient broadband photodetection has been brought about by incorporating QDs in PDs [365].

**Fig. 12:**
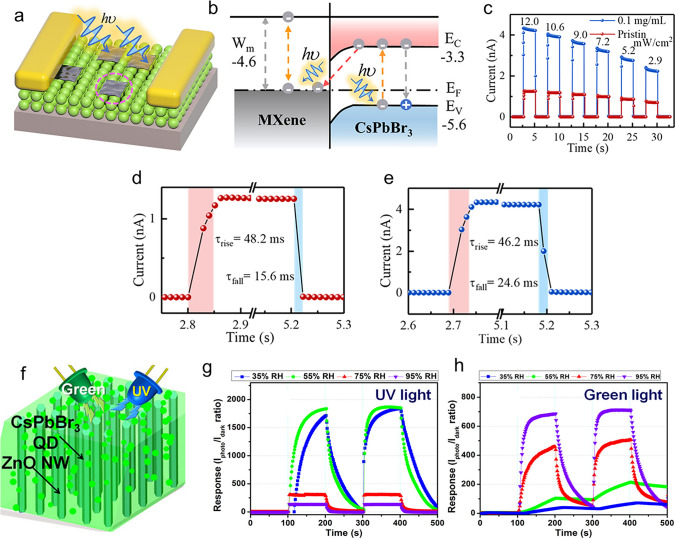
**a** Schematic illustration of CsPbBr_3_ QD/MXene NS PDs. **b** CsPbBr_3_/MXene thin-film energy band diagram when illuminated by light. **c** Plotting the devices’ photoresponse against different light intensities. The PDs’ responses for varying concentrations of MXene are shown as **d** 0 mg mL^−1^ and **e** 0.1 mg mL^−1^ for a single period. **a**–**e** Reproduce with permission [258]. Copyright 2022, Elsevier. **f** Schematic illustration. **g** PQD/ZnO NWs exposed to UV light; and **h** PQD/ZnO NWs’ reactions to RH detection in green light. **f**–**h** Reproduce with permission [257]. Copyright 2022, ACS

Exceptionally stable hybrid MXene NS/CsPbBr_3_ QD PDs were demonstrated (Fig. 12a) [258]. Figure 12b shows that the electrons at the conduction band (*E*_c_ ≈ 3.3 eV) tend to inject spontaneously into the 2D Ti_3_C_2_T_X_ NSs after excitation. Through intensifying carrier transportation, this phenomenon partially enhances the Ion. However, as the concentration of MXene NSs increases, the accumulation of 2D Ti_3_C_2_T_X_ NSs can cause the consumption of incident light to become dominant progressively, which will deteriorate the ion. Furthermore, 2D Ti_3_C_2_T_X_ NSs with high metallic conductivity have many free electrons, which efficiently concentrate incident light due to increase near-surface electromagnetic fields caused by collective electron oscillation [366]. As shown in Fig. 12c, the performance of the CsPbBr_3_ QD/MXene PDs was examined at various light intensities ranging from 2.9 to 12.0 mW cm^−2^ in order to better understand the enhancement effect. Compared to the pristine CsPbBr_3_ QD device, the PD containing 0.1 mg mL^−1^ 2D Ti_3_C_2_T_X_ NSs showed a much more noticeable response at every light power. As shown in Fig. 12c, the Ion of the detector containing 0.1 mg mL^−1^ 2D Ti_3_C_2_T_X_ NSs increased correspondingly from 2.25 to 4.27 nA with the increased light intensities. The devices’ τ_rise_, and τ_fall_ as shown in Fig. 12d, e, remained similar, within 50 and 20 ms, respectively, suggesting a reasonably quick response. In another study, Jian-Fu et al. [257] created a PD by integrating CsPbBr_3_ QDs with zinc oxide NWs (ZnO NWs). Schematic illustrations of the PQD/ZnO NWs’ structure are presented in Fig. 12f. The response of PQD/ZnO NWs/mica to UV and green light illumination as a function of RH is shown in Fig. 12g, h. The PQD/ZnO NW device exhibited greater responsivity under UV illumination than ZnO NWs. This is explained by the energy-level matching between ZnO and the CsPbBr_3_ film, which facilitates the transfer of photogenerated electrons into ZnO while strongly inhibiting carrier recombination. The sensing response of the PQD/ZnO NWs increased as RH increased, as shown in Fig. 12g. On the other hand, as Fig. 12h illustrates, the PQD/ZnO NW device’s responsivity was lower under green illumination than it was under UV illumination. The band gap of ZnO NWs (3.37 eV), which is higher than the energy of green light (2.38 eV), could be the cause of this discrepancy. Notably, at higher humidity levels, the sensors’ sensitivity to green light was more noticeable.

Due to its unique advantages over traditional materials, perovskite-based QDs are becoming highly preferred for advanced photodetection applications. QDs have outstanding broadband photodetection properties that allow them to detect light in a broad range of wavelengths, from ultraviolet to visible and infrared [364]. This offers various applications in various light spectrums and outperforms several standard PD materials. QDs also offer improved flexibility and performance in light detection processes because they can be precisely synthesized to customize their light absorption properties [364]. Moreover, QDs absorb visible light with the rare ability to release excited electrons or fluorescence, improving light-to-signal conversion efficiency and photodetection sensitivity. Their ability to work with various PD configurations shows how versatile they are, as they allow the creation of specially designed QD-based devices to meet the demands of certain applications. Because of its exceptional qualities—such as fluorescence emission, customized light absorption characteristics, broadband photodetection capabilities, and flexible device structures—QDs are a better material choice for PDs than traditional alternatives.

## Perovskite-Based Nanocrystals

### Growth of Perovskite-Based Nanocrystals

Perovskite NC growth mechanisms are complex, involving processes that are still poorly understood. Recent studies have shown that the growth of LHP NCs remains somewhat mysterious despite significant research efforts [367]. According to one study, self-assembly-driven dimensional growth is achieved through a novel growth mechanism that combines surface energetics and oriented attachment. Several variables, including acidity, organic ligands, and the precursors’ solubility, influence these NCs’ growth [368]. The most promising methods for producing perovskite NCs entail carefully considering elements like the growth environment’s acidity, the choice of organic ligands, and the solubility of the perovskite precursors [369]. Scholars are investigating novel techniques, like polymer-mediated in situ growth, to improve control over these NCs’ growth mechanisms [369]. Even though creating epitaxially grown all-inorganic perovskite–chalcogenide NC heterostructures presents inherent difficulties due to these materials’ complexity, this approach shows promise [370]. Notwithstanding these challenges, efforts are being made to improve these methods to realize the full potential of perovskite NCs for various uses.

**Fig. 13:**
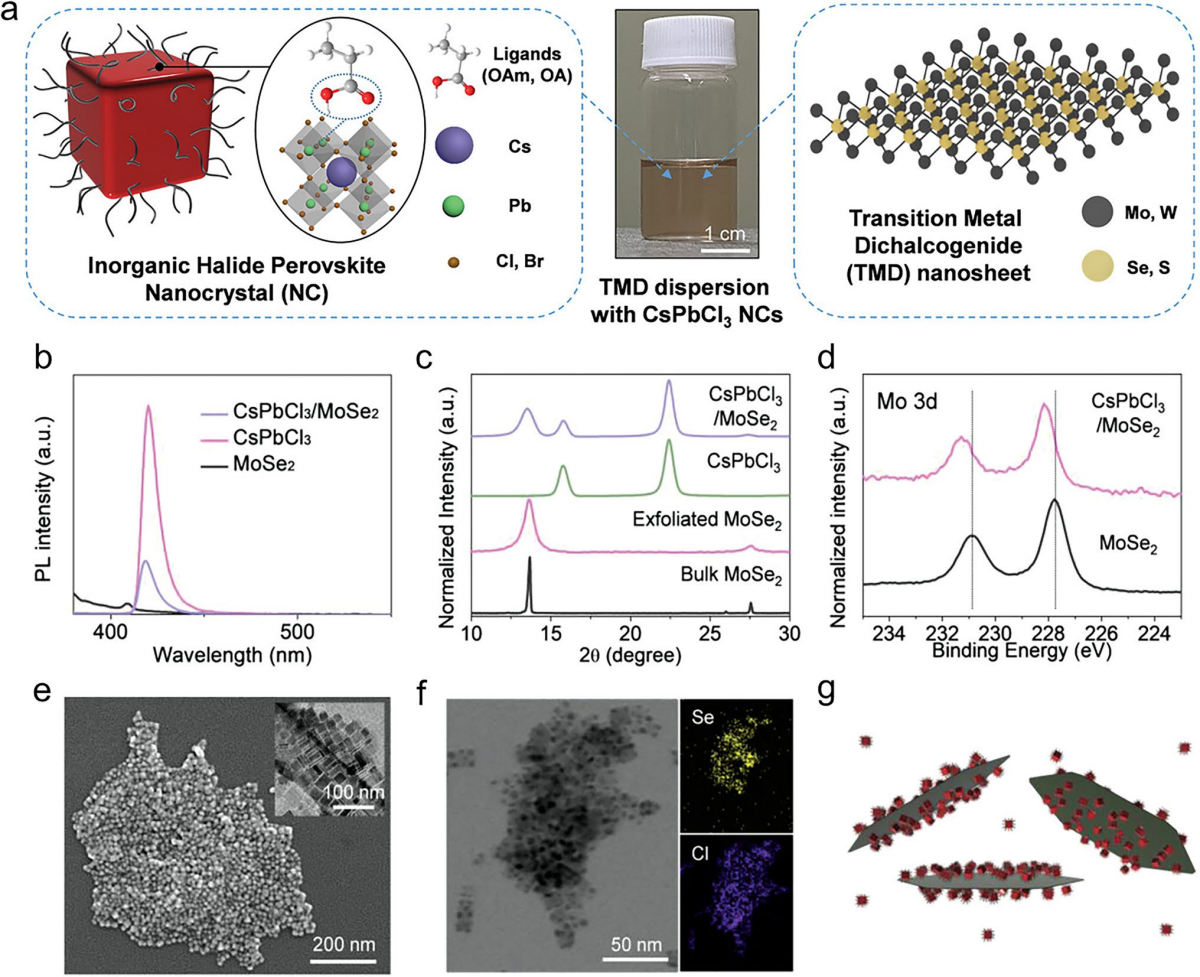
**a** Schematic illustration of an inorganic halide perovskite NC and a TMD nanosheet. A typical suspension of TMD (MoSe_2_) NSs stabilized with inorganic halide perovskite NCs (CsPbCl_3_) is shown in the picture. **b** CsPbCl_3_/MoSe_2_, CsPbCl_3_ NCs, and MoSe_2_ PL spectra. **c** CsPbCl_3_/MoSe_2_, CsPbCl_3_ NCs, exfoliated MoSe_2_, and bulk MoSe_2_ XRD patterns. **d** Mo 3*d* XPS profiles of the CsPbCl_3_ NCs and the CsPbCl_3_/MoSe_2_ nanocomposite. **e** A picture of the CsPbCl_3_/MoSe_2_ nanocomposite taken with a SEM. An image of CsPbCl_3_ NCs taken with a TEM is shown in the inset. **f** TEM picture of the CsPbCl_3_/MoSe_2_ nanocomposite; image of selenium and chlorine from EDX is shown on the right. **g** Schematic representation of CsPbCl_3_NCs (red dots) decorating and stabilizing MoS_2_ NSs (grey plates). **a**–**g** Reproduce with permission [277]. Copyright 2022, Wiley

The liquid-phase exfoliated transition metal dichalcogenide NSs are highly desirable for flexible and scalable photoelectronic applications. Even though dispersants like polymers, oligomers, and surfactants are used to exfoliate TMD NSs thoroughly, many of these substances are electrically insulating and need to be removed in order to keep the photoelectric qualities of the TMD NSs from deteriorating. Inorganic halide perovskite NCs of CsPbX_3_ (X = Cl, Br, or I) were introduced by Hyeokjung et al. [277] as a non-destructive dispersant that could disperse TMD NSs in the liquid phase. This method eliminates the need to remove the dispersant by improving the NSs’ photodetection capabilities. Hydrophobic oleic acids in hexane were used to passivate the surface of CsPbCl_3_ NCs during their synthesis, which involved a hot-injection technique. As shown in Fig. 13a, these obtained NCs were then used to disperse the MoSe_2_ NSs in a non-polar liquid phase [371]. After bulk MoSe_2_ was first sonicated in methyl ethyl ketone (MEK), the suspension’s supernatant was centrifuged. The MoSe_2_ NS precipitate produced by centrifugation was then distributed in a hexane solution containing CsPbCl_3_ NCs. After that, more centrifugation was used to produce highly dispersed MoSe_2_ NSs along with CsPbCl_3_ NCs, as shown in Fig. 13a. Raman spectroscopy and PL were used further to investigate the interaction between MoSe_2_ NSs and CsPbCl_3_ NCs, as shown in Fig. 13b. When excited with a laser (wavelength = 365 nm), the CsPbCl_3_ NCs in hexane showed a clear PL peak at about 420 nm, consistent with earlier findings. When the NCs were mixed with the MoSe_2_ NSs for exfoliation, their PL intensity significantly decreased, as shown in Fig. 13b. A photoinduced charge transfer may be transferred from the adsorbed CsPbCl_3_ NCs on the NS surface to the MoSe_2_ NS. This process could result in the production of excitons and the breakdown of positive trions, which would lower the intensity of the PL. The results show that the physical adsorption of CsPbCl_3_ NCs onto the NSs was used to disperse the MoSe_2_ NSs. The XRD data shown in Fig. 13c supports the idea that the presence of CsPbCl_3_ NCs promotes the exfoliation of MoSe_2_ NSs. When combined with CsPbCl_3_ NCs, the unique reflection seen at 13.52°—which corresponds to the (002) plane of 2H trigonal prismatic crystals of bulk MoSe_2_—became more expansive. This broadening is explained by the adsorption of NCs onto the MoSe_2_ NS in the CsPbCl_3_/MoSe_2_ nanocomposite, which disrupts the highly stacked layered structure [372]. It is important to note that after mixing with the MoSe_2_ NSs for exfoliation, the reflections at 15.78° and 22.42°, which are attributed to the (100) and (101) planes, respectively, of the cubic structure of the CsPbCl_3_ NCs, stayed mostly unchanged [371]. XPS was used to investigate further the interaction between the MoSe_2_ NS and CsPbCl_3_ NC. Specifically, the Mo 3*d* and Cs 3*d* peaks were analysed, as shown in Fig. 13d. Bright-field TEM and SEM were used to analyse the morphologies of the CsPbCl_3_/MoSe_2_ hybrid structures. Figure 13e, f shows the corresponding results, respectively. With a diameter of about 10 nm, the CsPbCl_3_ NCs successfully acted as a barrier between the NSs to lessen van der Waals interactions between neighbouring MoSe_2_ NSs. As shown schematically in Fig. 13g, this intervention produced a stable suspension of the CsPbCl_3_/MoSe_2_ hybrid. As explained in more detail in the section that follows, the inorganic halide perovskite NCs that adsorbed on the surface of the TMD NSs not only made the NSs easier to disperse but also improved their photodetection capabilities in a complementary way.

Researchers are actively tackling a number of the challenges associated with the synthesis of perovskite NCs for PDs to improve the devices’ efficiency and dependability. One of the main issues with perovskite NCs is their susceptibility to irreversible photodegradation, which can seriously affect their long-term stability and productivity [373]. Moreover, fluctuations in PL intensity are a reason for worry since they could lead to uneven performance from the apparatus [373]. Another major barrier is the chemical instability of perovskite NCs, which can break down under certain situations and jeopardize the longevity and functionality of devices [373]. Researchers are looking at novel production methods, material changes, and device engineering strategies to overcome these challenges and improve the stability, sensitivity, and overall performance of PDs based on perovskite NCs. These activities are critical to developing more robust and efficient PD technology for various applications.

### Perovskite Nanocrystal-Based Photodetectors

The remarkable photoelectric properties of perovskite NCs have attracted much interest in them as PDs. These detectors have a tunable band gap, excellent carrier migration behaviour, and effective light harvesting [64]. This study focuses on creating new materials, creating device architectures, and dissecting physical processes to enhance stability, sensitivity, and response time. Perovskite PDs come in different varieties, such as PDs, photodiodes, and PDs, depending on how they are made and work. Perovskite PDs, for example, use a metal–semiconductor–metal (MSM) coplanar structure to produce electron–hole pairs in response to incident photons. Studies have looked into using various materials, such as perovskite NCs made of CsPbBr_3_, to improve the efficiency of these PDs. Perovskite-based optoelectronics is a field that goes beyond photovoltaics, transistors, and light-emitting diodes. Perovskites are ideally suited for sensitive and quick PDs in optical communication, chemical/biological detection, image sensing, and environmental monitoring due to their high charge carrier mobility, efficient light absorption over a broad spectrum, and excellent photogeneration yield [18]. Scholars are investigating a range of material compositions, structures, morphologies, and device architectures to maximize the efficiency of perovskite-based PDs [18].

**Fig. 14:**
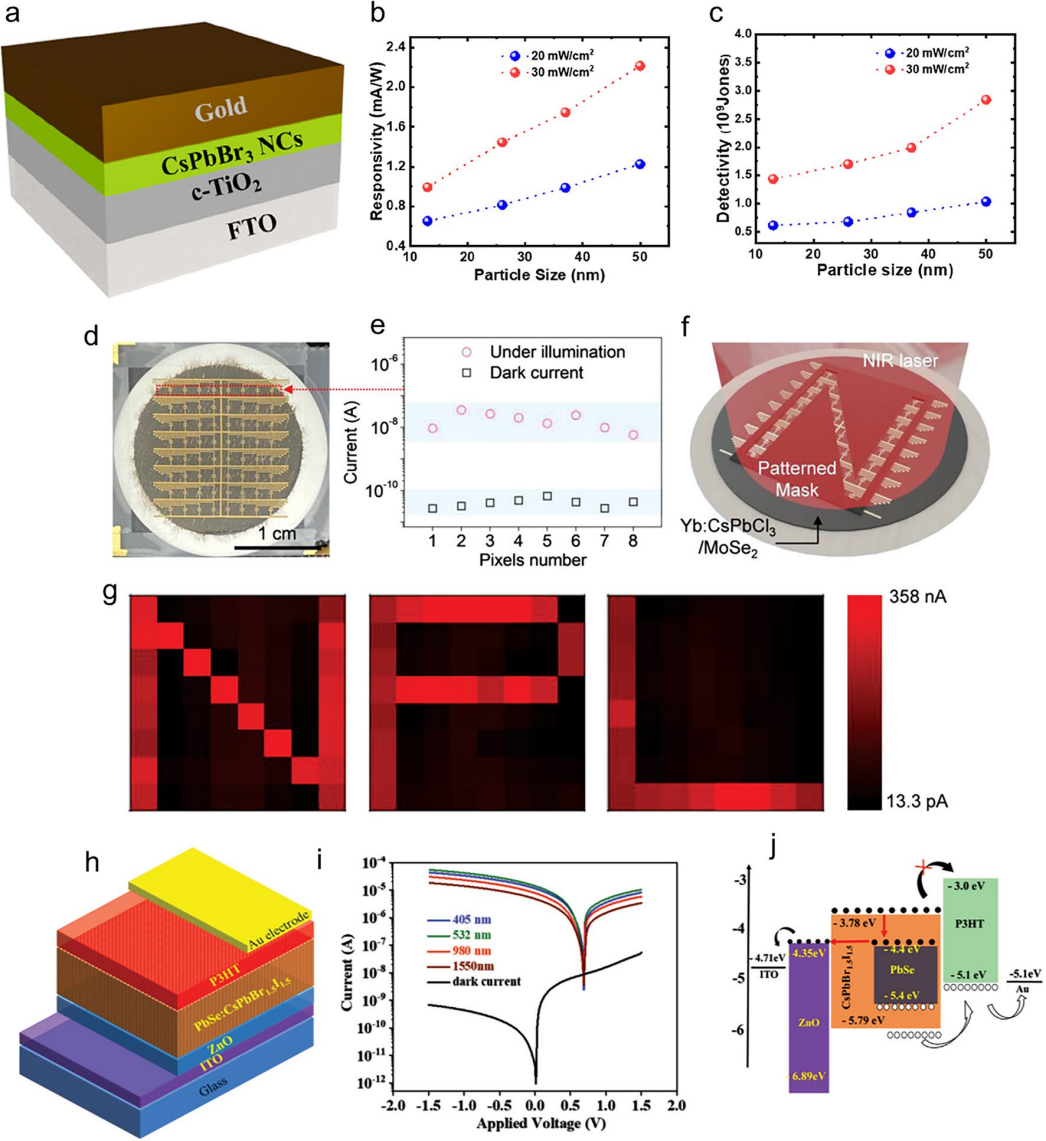
**a** A PD schematic illustration. **b, c** Each sample’s responsiveness and detectability **a-c**. Reproduce with permission [276]. Copyright 2023, ACS. **d** Yb^3+^:CsPbCl_3_/MoSe_2_ image sensor optical image. **e** Photocurrents in the first row of eight pixels in the dark and the light. **f** A schematic representation of the image sensor exposed to near-infrared light while wearing a patterned shadow mask. **g** Photocurrent mapping results of the image sensor exposed to a laser at 1064 nm with a power of 131.3 mW cm^−2^ using different shadow masks. **d**–**g** Reproduce with permission [277]. Copyright 2022, Wiley. **h** PD schematic diagram based on ITO/ZnO/PbSe: CsPbBr_1.5_I_1.5_/P3HT/Au. **i** TCAD simulations of dark current and photocurrent under various lasers with a 1 mW cm^−2^ power output. **j** Schematic energy band diagram showing the PD’s internal electron transport mechanism. **h**–**j** Reproduce with permission [81]. Copyright 2022, Wiley

Inorganic halide perovskite NCs have garnered significant attention in recent years owing to their enhanced stability, superior photophysical properties, and defect-tolerant nature, enabling high carrier mobility and efficient charge transport in diverse optoelectronic devices. The band gap and size of halide perovskite NCs can be adjusted through various methods, such as cation/anion exchange, ligand modification, and precursor concentration adjustment. The effects of precursor concentration on the structural, optical, and electronic characteristics of CsPbBr_3_ NCs were investigated by Atif et al. [276]. The schematic of the prepared device is shown in Fig. 13a. When the particle size increases, the PD’s responsivity to UV light increases from about 0.1 to 2.21 mA W^−1^ under 30 mW cm^−2^ light intensity, as shown in Fig. 14b, without any bias applied. This phenomenon can be explained by larger NCs having fewer surface defects and available trap states due to their lower surface-to-volume ratio. On the other hand, when the NC’s size decreases, the surface-to-volume ratio rises, resulting in a greater amount of surface imperfections available for charge carriers. In this case, trap conditions are established on the surface of the NCs that effectively promote charge recombination [374]. Figure 14c shows the highest detectivity for UV light at 2.84 × 10^9^ Jones (1 Jones = 1 cm Hz^0.5^ W^–1^) under 30 mW cm^−2^ light intensity. This value is in line with PDs based on perovskite NCs that have been previously reported [342, 375]. On the other hand, another sample exhibits the lowest detectivity for green light at 2.75 × 10^7^ jones under 5 mW cm^−2^ light intensity. The findings from earlier research on perovskite PDs are consistent with the observed increase in response and detection with increasing incident light power [142]. In another study, creating thin, homogeneous Yb^3+^:CsPbCl_3_/MoSe_2_ films with excellent photodetection capabilities made building arrays of PDs appropriate for wide-area image recognition possible [277]. Figure 14d shows the development of a wafer-scale image sensor with 8 × 8-pixel arrays of Yb^3+^:CsPbCl_3_/MoSe_2_ PDs. The photocurrent and dark currents of the top eight pixels (first single row) were first statistically analysed. Figure 14e shows consistent photocurrent and dark current across all devices, with only minor variations. Furthermore, a notably increased *I*_ON_/*I*_OFF_ ratio of greater than 100 was verified. In order to showcase the sensor’s imaging ability, Fig. 14f shows how an NIR laser was used to light up the sensor’s active regions, which were delineated by a shadow mask. The characters "N," "P," and "L," each defined by the shadow masks, can be easily recognized in the photocurrent map, as shown by the 2D photocurrent maps shown in Fig. 14g. These findings imply that the arrays of Yb^3+^:CsPbCl_3_/MoSe_2_ PDs are a good choice for creating near-infrared image sensors suitable for outdoor use. It is important to note that different pattern fabrication techniques could improve the resolution of our image sensor, which has 64 arrays of devices. Using different combinations of perovskite NCs and TMD NSs allows for the easy design of emerging photoelectronic materials that can be tailored to specific properties through solution processing. Also, Muhammad et al. [81] created bulk-heterojunction-based high-performance PDs by combining PbSe colloidal QDs (CQDs) with all-inorganic mixed halide perovskite NCs in a hybrid nanocomposite. Figure 14h shows a schematic representation of the self-powered, broadband PD, in which P3HT acts as the hole-transporting layer and ZnO as the electron-transporting layer. The device’s photocurrent and simulated dark, as obtained from *J*–*V* maps, are shown in Fig. 14i. The simulated curves also show the apparatus’s self-powered photoresponse, supporting the validity of the experimental findings. A schematic energy band diagram, as shown in Fig. 14j, can clarify the photocurrent generation and carrier transport mechanism through the PD ITO/ZnO/PbSe: CsPbBr_1.5_I_1.5_/Au. Excitons are created when light is absorbed and moves to the interface of the electron-transporting layer, where holes stay in the HOMO level and electrons reside at the LUMO level due to thermodynamic reasons. Intermolecular charge transfer states maintain the coulombic bound state of these electrons and holes at the interfaces. Because the LUMO levels of the two materials differ, energy band bending takes place at the interface between PbSe QDs and CsPbBr_1.5_I_1.5_ NCs to reach an equilibrium state. The separation of photogenerated electrons and holes is then facilitated by the establishment of a strong built-in electric field inside the BHJ. The electron-extracting layer (ZnO) and the hole-extracting layer (P3HT) subsequently move these separated carriers in the direction of the corresponding electrodes. Using TCAD software, numerical simulation was used to further analyse the optoelectronic properties, such as the device’s intrinsic electrostatic potential, absorbed photon density, exciton generation, electric current density, and electron density. It is discovered that these simulation results agree with the experimental data.

The research is ongoing to find solutions for the issues related to the application of perovskite NCs in PDs. Researchers are investigating innovative approaches to improve the overall performance, sensitivity, and stability of perovskite-based PDs. Narrowband detection techniques, creative device architectures, and applications targeted at resolving current Perovskite PDs’ limitations are examples of recent advancements in this field [15, 376]. To increase stability and functionality in sensing applications, methods like using low-volatility polar solvents, looking into lead-free substitutes, and researching metal halides are being investigated [376]. Moreover, researchers are focusing on the creation and use of halide perovskite NCs in optoelectronics to improve PDs’ performance [373]. These endeavours aim to enhance the overall performance, stability, and sensitivity of perovskite NCs for photodetection applications. To put it briefly, current research efforts are concentrated on finding new ways to improve the stability and performance of Perovskite NCs in PDs through cutting-edge materials, creative device designs, and novel strategies.

## Perovskite Nanostructure-Based Polarization-Sensitive Photodetectors

PDs that utilize self-assembled perovskite NSs or aligned perovskite NWs can efficiently yield data regarding light polarization. These materials’ anisotropic structure, which makes them sensitive to the direction of incoming light waves, gives rise to this functionality. Recent developments with aligned perovskite NWs have demonstrated substantial promise for polarization-sensitive PDs. A noteworthy study showed how to align NWs using a brush coating technique to create a flexible, polarization-sensitive PD. These NWs’ alignment creates an anisotropic structure that responds differently to light polarized in different directions [226]. Since anisotropic structures are typically necessary for PDs to have polarization sensitivity, the anisotropic nature of these materials is essential for detecting polarized light. The necessary anisotropy is provided by the 2D arrangement of nanosheets or the one-dimensional structure of NWs. For example, one study used one-dimensional NWs to fabricate a polarization-sensitive ultraviolet (UV) PD. These NWs had a high photocurrent anisotropy ratio of about 3.16 due to their external morphology anisotropy and asymmetric structure’s electric and optical anisotropy [377]. This suggests that the apparatus reacted differently to light polarized in various directions, successfully obtaining polarization information. Polarization-sensitive PDs based on perovskites have many benefits, such as high detectivity and responsivity, quick response times, adaptability, stability, and potential uses in various spectral ranges, including UV. These PDs’ advancement opens new avenues for use in polarization information-critical optoelectronic devices such as imaging systems and optical communications.

**Fig. 15:**
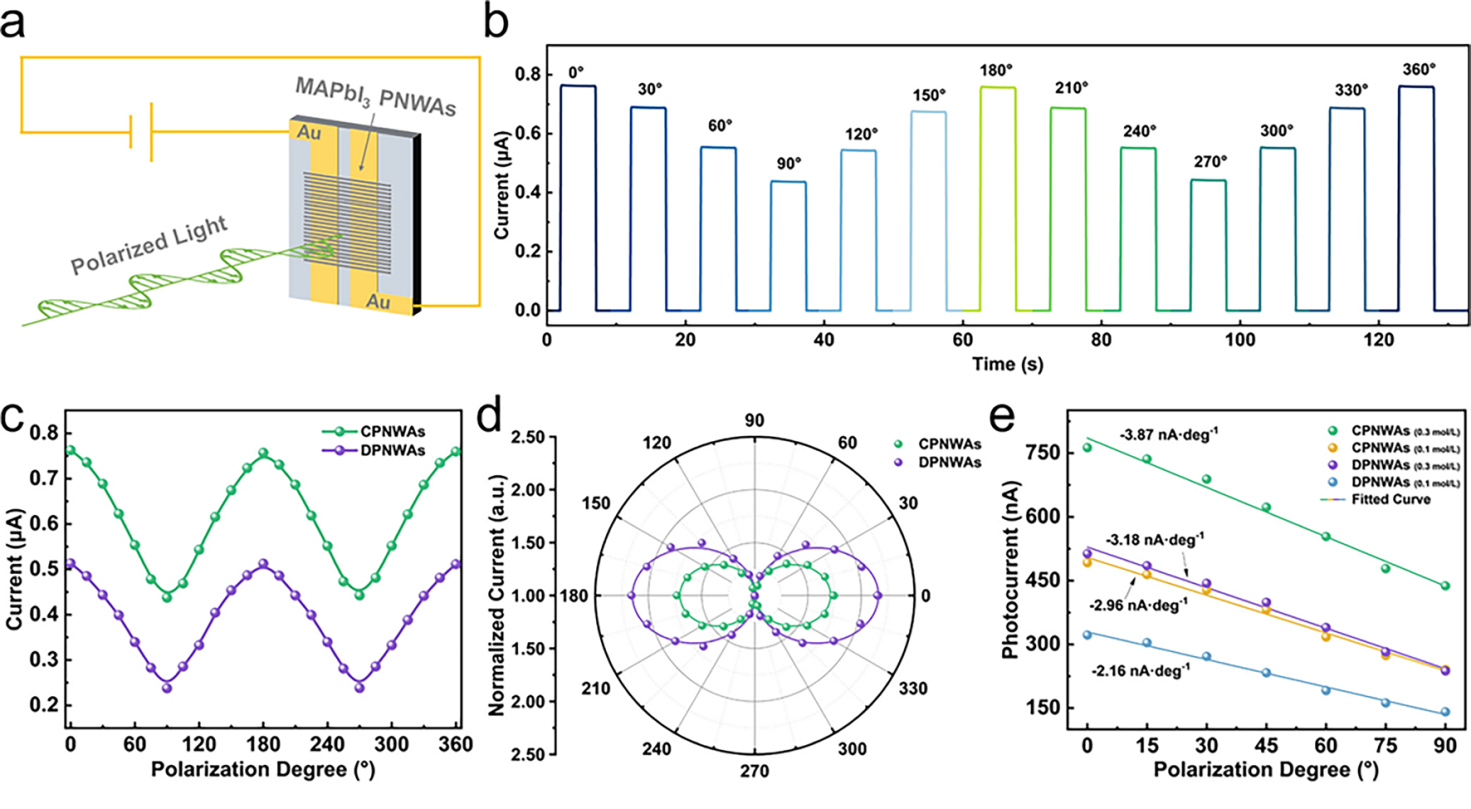
**a** Diagram demonstrating the orientation of the polarized photoelectric vector parallel to the direction of perovskite NW growth at a polarization angle of 0°, illustrating the detection of polarization sensitivity in perovskite NWs. **b** CD-ROM patterns NWs’ photocurrent response over time to different polarization angles of incident light. **c** Curves show how the photocurrent of perovskite NWs at different polarization angles changes. **d** A normalized polar plot with values corresponding to the photocurrent at a 90° polarization angle, demonstrating the polarization-dependent photocurrent of perovskite NWs. **e** Photocurrent response of perovskite NWs varies in morphology from 0° to 90° polarization angles. **a**–**e** Reproduce with permission [226]. Copyright 2024, ACS

Lu et al. [226] recently used template-confined growth (TCG) techniques based on CD-ROM and DVD-ROM grating patterns to create perovskite NWs with crystallographic alignment, different line widths, and alignment densities. Perovskite NWs have excellent optoelectronic characteristics and anisotropic optical absorption properties, which make them perfect for polarization detection. In order to investigate this, polarized light was produced by passing natural light from a xenon lamp through a linear polarizer in a conventional sunlight simulator. The first test configuration with a polarization angle of 0° is shown in Fig. 15a, where the axial direction of the perovskite NWs and the plane of electric vector vibration of the linearly polarized light are parallel. The quick reaction of the CD-ROM patterns NWs device to polarized light is shown in Fig. 15b, which also shows a clear photocurrent dependence on polarization angle. At a polarization angle of 0°, the maximum values of polarized photocurrent were observed; at 90°, the photocurrent reached its minimum. The polarization-dependent photocurrent of the device then showed periodic oscillations as the polarization angle increased further, peaking at 180° and decreasing to a minimum at 270°. The polarization-dependent photocurrents of DVD-ROM patterns NWs and CD-ROM patterns NWs at a bias voltage of 1 V are shown in Fig. 15c. Photocurrents that vary with the polarization angle and follow cosine waveform patterns are seen in both devices. The cosine waveform patterns in the photocurrents of both devices varied with the polarization angle. In contrast to DVD-ROM patterns NWs, CD-ROM patterns NWs displayed larger polarized photocurrents. This difference can be explained by the wider and thicker single NWs found in CD-ROM patterns, which make carrier transport easier. The polarization ratios of CD-ROM patterns NWs and DVD-ROM patterns NWs are 1.81 and 2.16, respectively, as shown by the symmetrical "∞" structure of the polar coordinate-normalized polarization photocurrent in Fig. 15d (where *I*_max_/*I*_min_ is the ratio of maximum to minimum polarization photocurrent values at 0° and 90° angles). This ratio roughly corresponds to the anisotropy ratio of polarized light absorption, suggesting that the polarization sensitivity brought about by the 1D nanograting patterns of perovskite NWs devices is the source of their polarization-sensitive photoelectric detection properties.

In practical device applications, the analysis of the photocurrent dependence on polarization angle was further investigated. The TCG method was used to create CD-ROM patterns NWs with finer dimensions by varying the concentration of the precursor to 0.1 mol L^−1^. The photocurrent of every perovskite NW device dropped linearly as the polarization angle progressively changed from 0 to 90°, as shown in Fig. 15e. Polarization sensitivities, which are measured as the linear change in photocurrent per degree change in polarization angle or the slope of the polarized photocurrent, were shown by the CD-ROM patterns NWs and DVD-ROM patterns NWs prepared with a precursor concentration of 0.3 mol L^−1^ to be − 3.87 and − 3.18 nA deg^−1^, respectively. Similarly, polarization sensitivities of − 2.96 and − 2.16 nA deg^−1^ were observed in CD-ROM patterns NWs and DVD-ROM patterns NWs prepared with a precursor concentration of 0.1 mol L^−1^. Devices of finer-sized CD-ROM patterns NWs had higher polarization ratios (2.04 and 2.28 for CD-ROM patterns NWs and DVD-ROM patterns NWs prepared with 0.1 mol L^−1^ precursor concentration, respectively). This is likely because the finer-sized NWs improved aspect ratios on a spatial scale. The optoelectronic performance of the devices is somewhat compromised by the strategy of obtaining finer-sized perovskite NWs by reducing precursor concentration in order to improve polarization ratios. To enable polarization identification across different wavelengths in future practical applications of polarization detection, templates with different spacing dimensions must be customized. Furthermore, resolving the homogeneity problems in large-area nanowire arrays is essential to guaranteeing detection accuracy and reproducibility. These efforts are crucial to maximize the effectiveness and adaptability of perovskite NWs devices in a variety of optical sensing applications.

Nanoscale perovskites with chiral ligands represent a developing area of materials science; these materials have unique chiroptical characteristics and may be used in optoelectronics and spintronics. Chiral perovskite nanostructures have been successfully synthesized by researchers using a variety of techniques. For example, chiral phosphate molecules have been used to demonstrate anionic ligand-induced chirality with nanoscale perovskite CsPbBr_3_, retaining their chiroptical characteristics after purification [378]. The introduction of Cotton effects in CsPbBr_3_ nanoparticles, indicating effective electronic coupling with the nanoparticles, has been made possible by post-synthetic modification involving chiral amines such as 1-phenylethylamine [379]. Furthermore, a ligand exchange strategy has achieved chiral perovskite NCs with controlled chiroptical properties and a high PLQY [380]. Chiral-induced spin selectivity (CISS) effect has been utilized in engineering dual-ligand quasi-2D perovskite structures for room-temperature spin LEDs [381]. These developments demonstrate how the special chiroptical characteristics of chiral perovskite nanomaterials can be used to explore CISS and advance optoelectronic applications. The combination of the optical characteristics of perovskites and the chirality-induced effects of ligands presents a promising material for the efficient and direct detection of circularly polarized light (CPL) in nanoscale perovskites with chiral ligands. The chirality-induced absorption of chiral ligands in 2D Ruddlesden–Popper perovskites makes them promise for CPL detection [382]. Flexible CPL detectors on substrates such as PET are made possible by these solution-processable materials. Studies on chiral ligands in perovskite QDs indicate that they may be able to induce electron exchange or surface lattice distortions that lead to circularly polarized luminescence [383]. Chiral ligand nanoscale perovskites offer a promising platform for CPL detectors, and further study is expected to improve device performance and broaden the range of applications in opto-spintronics, quantum communication, and polarization-sensitive imaging.

## Challenges and Prospects

**Fig. 16:**
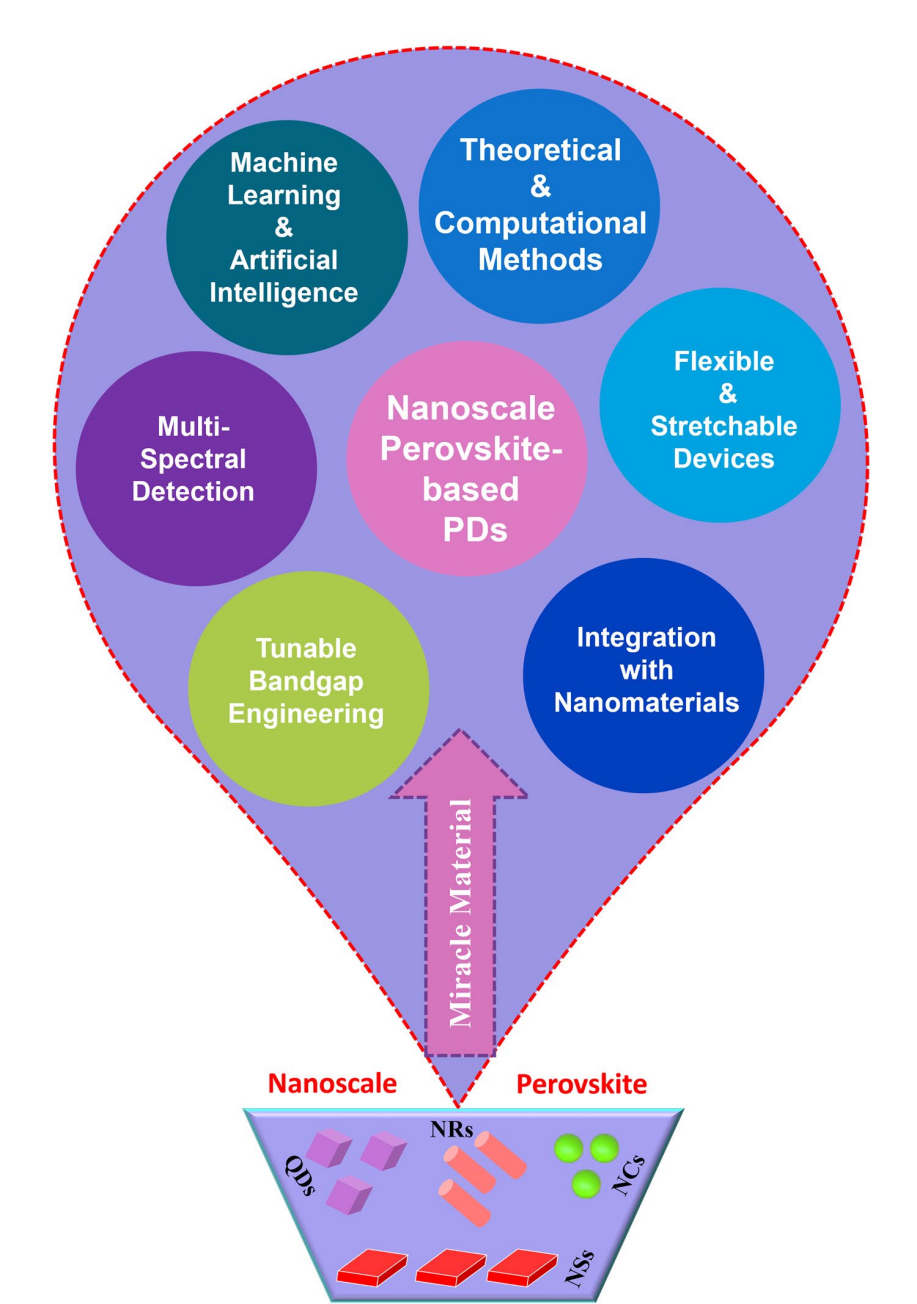
Future aspect of nanoscale perovskites

1. When exposed to oxygen and moisture, perovskites are prone to degradation, compromising their stability and hindering commercial applications until new protective strategies, like heterojunction structures, are implemented [51]. Because lead-based perovskite materials such as MAPbI_3_ and CsPbBr_3_ are toxic, safe usage requires the development of environmentally friendly substitutes with non-toxic components [51]. In order to achieve optimal device performance, it cannot be easy to optimize photodetection parameters in both PDs and phototransistors, such as high responsivity, large detectivity, and rapid speed. Some studies only focus on a device’s peak performance, ignoring average performance, which could mislead industrialization efforts and suggest a lack of confidence in the stability of the device [51].
2. The difficulties in producing crystalline perovskite NSs may be overcome by using novel strategies and methods that have been discovered recently. An alternative to directly growing perovskite single crystals is fabricating large-sized LHP NSs from single crystals, which allows for the production of centimetre-sized free-standing NSs [47, 319]. Furthermore, it has been suggested that producing thin single crystals at micro- and nanoscales could improve structural stability and solve problems encountered when synthesizing organic–inorganic LHP single crystals [44]. Furthermore, it has been proposed that improvements in the kinetics and pathways of crystallization could enhance the quality and reproducibility of solution-processing techniques for MHPs, such as NSs [384]. These solutions serve as a reminder of the continuous efforts to get past obstacles and maximize the development of perovskite crystalline NSs for a range of optoelectronic and other applications. Perovskite-based PDs have advanced significantly in recent times, with a focus on many aspects of their architecture and functionality. Studies have shown how narrowband perovskite PDs have advanced, showcasing creative strategies, new methods, and fundamental physics [332]. Additionally, research has examined advancements in perovskite-based organic–inorganic hybrid PDs, presenting promising strategies to increase their efficiency [52]. Furthermore, it has been discovered that nanostructured perovskites are ideally suited for photodetection applications because of their high carrier mobility, extended carrier lifespan, and simplicity of device integration [51]. Collectively, these advancements demonstrate how perovskite-based PDs are always changing and can potentially broaden their use and detection range.
3. A proposed method to address the challenges of hybrid perovskites for integrated optoelectronics focuses on many key features. The most important thing is to look for new fabrication techniques to produce hybrid perovskite materials with nanoscale thicknesses while maintaining their structural and optical integrity. This can mean adjusting solution processing methods like spray coating or inkjet printing that offer precise control over film thickness. Second, the stability of hybrid perovskite materials used in optoelectronic devices must be increased. Research efforts should focus on developing novel encapsulation methodologies, interface engineering techniques, and material compositions that fight degradation factors such as moisture and heat instability. Stable organic or inorganic passivation layers can also increase the long-term stability of hybrid perovskite devices. Moreover, achieving outstanding optoelectronic performance requires a deep understanding of the physics of the device and the material. Therefore, comprehensive investigations are needed to optimize hybrid perovskite-based device designs, interfaces, and charge transport processes. This may include creating interfaces between layers, changing the composition of hybrid perovskite materials, and fine-tuning device geometries to enhance charge extraction and carrier mobility. Moreover, advancement in this subject depends on collaboration between theoretical physicists, device engineers, and materials scientists. By combining computational modelling and simulation methods with experimental observations, scientists can better understand the basic principles that control hybrid perovskite device performance. This will help create more reliable and effective optoelectronic devices. A multidisciplinary approach involving materials synthesis, device fabrication, and theoretical modelling is required to overcome the challenges with hybrid perovskites and realize their potential for integrated optoelectronics with nanoscale thickness, high stability, and exceptional performance.
4. Perovskite NR arrays in solar systems exhibit instability due to their susceptibility to various environmental conditions such as heat, moisture, and light [385, 386]. These elements may contribute to deterioration and affect the device’s long-term functionality. A range of strategies are employed to improve stability, including investigating novel perovskite structures, perfecting encapsulation methods, and understanding degradation mechanisms [385, 386]. The primary source of known harmful element (Pb) in perovskite materials is a cause for concern regarding toxicity. Pb^2+^ ions that are dissolved in water have the bioavailability to become hazardous, which poses a risk to both human health and the environment. Some solutions to address toxicity include using lead-free perovskite materials, implementing Pb recycling technologies, encapsulating devices to stop Pb leakage, and immobilizing lead inside the devices [387]. These programmes aim to reduce the hazards that lead-containing perovskite NR arrays used in solar cells cause to the environment and public health. Stability issues in perovskite NR arrays are often resolved via ligand engineering, encapsulation, metal cation dopants, and modification of the production process. By shielding the perovskite NCs from external factors like oxygen and moisture, encapsulation approaches aim to improve their stability [388]. Through ligand engineering, the surface chemistry of the NCs is altered to improve stability and halt deterioration [388]. Adding metal cation dopants can increase stability by altering the crystal structure and properties of the perovskite NCs [388]. Fabrication process optimization aims to improve manufacturing methods to create more stable and dependable perovskite NR arrays for solar applications [388]. These methods all assist in resolving stability concerns and enhancing the efficiency of perovskite NR arrays in solar energy systems.
5. One issue in the growth of perovskite NWs is controlling it for specific uses. The challenges include the requirement for inorganic perovskite NWs to passivate defects at grain boundaries, which can be challenging. Other challenges include guiding growth to create a 2D surface appropriate for solar cell applications, which can be challenging [344] and patterning, aligning, and transferring perovskite NWs for use in nanophotonics and lighting [58]. These difficulties demonstrate how crucial it is to advance growing techniques to utilize perovskite NWs’ potential in various technological applications fully. The intricate processes required for patterning, aligning, and transferring perovskite NWs for usage in lighting and nanophotonics are one of the challenges in integrating them into lighting technologies [58]. Further complicating matters, achieving regulated growth to provide an acceptable 2D surface for solar cell applications poses a challenge to the efficient application of perovskite NWs [144]. Furthermore, certain topologies, like CsPbBr_3_ NWs, may be challenging to induce directional development, which has an impact on how effectively they integrate into lighting systems [57]. These challenges underscore the importance of enhancing growth tactics to surmount obstacles to the assimilation of perovskite NWs into lighting technology.
6. PQD synthesis is extremely challenging because of its sensitivity to environmental factors and the need for precise control over the synthesis process. Among these challenges is structural instability brought on by PQDs’ low formation energy, which can result in structural changes when the particles interact with polar solvents, halogens, water, light, and oxygen [389]. PQDs without flaws and homogeneity can only be made by a rigorous synthesis procedure involving meticulous ligand, solvent, and reaction condition selection [390]. The fact that PQDs are sensitive to environmental changes further emphasizes the necessity of controlled synthesis conditions to prevent mistakes and ensure high-quality QDs [391]. Even though these materials are less harmful than conventional materials like cadmium-based QDs, eliminating toxic impurities during synthesis remains difficult. It also takes precise control over the reaction parameters to maintain size, shape, and composition homogeneity and provide high yields [392]. It is essential to solve these problems with advanced synthesis methods and ongoing stability strategy research if PQDs are to reach their full potential in various applications.
7. Although perovskite NCs with exact sizes and shapes can now be manufactured, research into the fundamental ideas behind their production is still ongoing [369]. Further research is needed to manage the development process and better understand the underlying mechanisms [367]. The growth of perovskite NCs presents several challenges that scientists are attempting to overcome. These challenges include the growing environment’s acidity, selecting appropriate organic ligands, and the solubility of perovskite precursors, all of which significantly affect the method by which these NCs grow [369]. Moreover, the complexity of these materials presents special challenges when developing epitaxially grown all-inorganic perovskite–chalcogenide NC heterostructures [370]. To overcome these challenges, scientists are exploring new approaches, such as growing perovskite NCs inside bifunctional metal–organic framework (MOF) matrices to gain more control over their growth and properties [393]. Despite these challenges, ongoing research endeavours seek to surmount them and unlock the complete potential of perovskite NCs for various applications.
8. Using perovskite NCs in PDs has several disadvantages, including issues with stability, moisture sensitivity, and device tuning challenges. Although perovskite NCs possess unique optical and electrical features, stability remains problematic for practical applications. Environmental factors like dampness may impact these NCs’ lifetime and performance. Perovskite NC-based PDs also face high photosensitivity and detectivity challenges, necessitating further improvement of their device designs and operating processes [65]. In order to enhance the overall performance of perovskite-based PDs, scientists are presently striving to enhance their stability, sensitivity, response time, and noise levels. In conclusion, the primary disadvantages of using perovskite NCs in PDs include stability issues, moisture sensitivity, and the ongoing requirement for modification to achieve high photosensitivity and detectivity. These limitations must be removed to exploit perovskite NCs for photodetection applications properly.
9. The study of nanoscale perovskites is developing quickly, and one important method for manipulating morphology and improving material characteristics is ligand engineering. In order to achieve precise control over the shape, size, and surface properties of nanoscale perovskites, future research could probably concentrate on designing novel ligands. It is anticipated that multifunctional ligands will simplify synthesis procedures by concurrently controlling morphology, enhancing stability, and improving optoelectronic properties. Additionally, efforts will be made to improve long-term stability through stronger ligand binding, refining particular crystal facets, and use greener synthesis techniques. With advanced techniques for in situ ligand manipulation and computational modelling, new experimental design guidance for hybrid structures and morphologies of nanoscale perovskites may be possible. As the field advances, optimization of the ligand choice and the morphology of nanoscale perovskites tailored to specific applications will likely enhance the stability, performance, and versatility of these materials in a range of applications, such as solar cells, LEDs, and quantum computing devices.
10. Stemming from optimized nanostructure designs and fabrication techniques, perovskite nanostructure-based polarization-sensitive PDs are positioned for major progress in improving performance metrics like responsivity and polarization sensitivity. They could find new uses in medical imaging and environmental monitoring if their spectral range is extended to include the ultraviolet and mid-infrared. Multifunctionality will be improved by integration with flexible electronics, wearable technology, and Internet of Things sensors; long-term reliability will be addressed by efforts to improve stability and durability through improved encapsulation and strong material development. Superior performance and large-scale production will be made possible by innovative nanostructure designs, such as hybrid and hierarchical architectures, and scalable fabrication techniques. Artificial intelligence could make intelligent, real-time data processing possible, and quantum-enhanced sensing could provide previously unheard-of levels of sensitivity. In order to ensure sustainable and effective PDs, future research could also examine multifunctional devices and environmentally friendly materials. Further research is required to overcome these obstacles before these innovative devices can be widely adopted and commercialized.
11. Perovskite photodetection may develop due to stability, responsiveness, and dimensional engineering improvements. Functionality may be improved by integrating nanomaterials and mixed-dimensional methods, and flexible fabrication techniques may enable scalable systems. Compatibility with silicon technology may help to facilitate integration further. Concentrating on environmental sustainability, optimization, and the investigation of new derivatives may be essential to expand the applications. Machine learning and theoretical methods could become increasingly useful in material design. The remarkable characteristics of perovskites have the potential to stimulate advancements in optics, electronics, and thermal management, thereby influencing numerous other fields in the future. These important future considerations are emphasized in Fig. 16.

## Conclusions

In conclusion, there is much promise for revolutionizing optoelectronic applications in the developing field of nanoscale perovskite PDs. Researchers can now remarkably control perovskite nanomaterials’ size, shape, and composition, resulting in previously unheard-of performance gains. These improvements provide improved stability, photoresponse, sensitivity, and spectral tunability. Even so, major issues must be resolved before widespread commercial use, including ambient stability, fabrication scalability, and toxicity concerns. In order to overcome these challenges and advance nanoscale perovskite PDs, collaboration between materials scientists, chemists, and engineers is imperative. As the field develops, perovskite PDs will move closer to commercial viability and useful applications through additional research into innovative synthesis techniques, interface engineering, and device architectures. These detectors have the potential to transform optoelectronic technology completely through continued innovation and interdisciplinary cooperation. They offer unparalleled performance and adaptability for various uses, including energy harvesting, imaging, sensing, and communication.
